# Home-based exercise program in the indeterminate form of Chagas disease (PEDI-CHAGAS study): A study protocol for a randomized clinical trial

**DOI:** 10.3389/fmed.2022.1087188

**Published:** 2023-01-06

**Authors:** Mauro F. F. Mediano, Leonardo G. Ribeiro, Rudson S. Silva, Isis G. G. Xavier, Marcelo C. Vieira, Tatiana R. Gonçalves, Vitor B. Paravidino, Juliana P. Borges, Luiz Fernando Rodrigues Junior, Henrique S. Costa, Michel S. Reis, Livia C. Liporagi-Lopes, Pablo Martinez-Amezcua, Paula S. Silva, Gilberto M. Sperandio Da Silva, Andrea S. Sousa, Marcelo T. Holanda, Henrique H. Veloso, Fernanda M. Carneiro, Flavia Mazzoli-Rocha, Andrea R. Costa, Roberto M. Saraiva, Fernanda S. N. S. Mendes, Luiz Henrique C. Sangenis, Alejandro M. Hasslocher-Moreno

**Affiliations:** ^1^Evandro Chagas National Institute of Infectious Diseases, Oswaldo Cruz Foundation, Rio de Janeiro, RJ, Brazil; ^2^Department of Research and Education, National Institute of Cardiology, Rio de Janeiro, RJ, Brazil; ^3^Center for Cardiology and Exercise, Aloysio de Castro State Institute of Cardiology, Rio de Janeiro, RJ, Brazil; ^4^Institute of Collective Health, Federal University of Rio de Janeiro, Rio de Janeiro, RJ, Brazil; ^5^Institute of Social Medicine, State University of Rio de Janeiro, Rio de Janeiro, RJ, Brazil; ^6^Department of Physical Education and Sports, Naval Academy – Brazilian Navy, Rio de Janeiro, RJ, Brazil; ^7^Institute of Physical Education and Sports, State University of Rio de Janeiro, Rio de Janeiro, RJ, Brazil; ^8^Physical Therapy Department, Federal University of Jequitinhonha and Mucuri Valleys, Diamantina, MG, Brazil; ^9^Faculty of Physical Therapy, School of Medicine, Federal University of Rio de Janeiro, Rio de Janeiro, RJ, Brazil; ^10^Faculty of Pharmacy, Federal University of Rio de Janeiro, Rio de Janeiro, RJ, Brazil; ^11^Department of Epidemiology, Johns Hopkins Bloomberg School of Public Health, Baltimore, MD, United States

**Keywords:** physical activity, exercise training, mental health, fitness, Chagas disease, quality of life

## Abstract

**Background:**

Chagas disease (CD) is a neglected endemic disease with worldwide impact due to migration. Approximately 50–70% of individuals in the chronic phase of CD present the indeterminate form, characterized by parasitological and/or serological evidence of *Trypanosoma cruzi* infection, but without clinical signs and symptoms. Subclinical abnormalities have been reported in indeterminate form of CD, including pro-inflammatory states and alterations in cardiac function, biomarkers and autonomic modulation. Moreover, individuals with CD are usually impacted on their personal and professional life, making social insertion difficult and impacting their mental health and quality of life (QoL). Physical exercise has been acknowledged as an important strategy to prevent and control numerous chronic-degenerative diseases, but unexplored in individuals with the indeterminate form of CD. The PEDI-CHAGAS study (which stands for “Home-Based Exercise Program in the Indeterminate Form of Chagas Disease” in Portuguese) aims to evaluate the effects of a home-based exercise program on physical and mental health outcomes in individuals with indeterminate form of CD.

**Methods and design:**

The PEDI-CHAGAS is a two-arm (exercise and control) phase 3 superiority randomized clinical trial including patients with indeterminate form of CD. The exclusion criteria are <18 years old, evidence of non-Chagasic cardiomyopathy, musculoskeletal or cognitive limitations that preclude the realization of exercise protocol, clinical contraindication for regular exercise, and regular physical exercise (≥1 × per week). Participants will be assessed at baseline, and after three and 6 months of follow-up. The primary outcome will be QoL. Secondary outcomes will include blood pressure, physical fitness components, nutritional status, fatigability, autonomic modulation, cardiac morphology and function, low back pain, depression and anxiety, stress, sleep quality, medication use and adherence, and biochemical, inflammatory and cardiac biomarkers. Participants in the intervention group will undergo a home-based exercise program whilst those in the control group will receive only general information regarding the benefits of physical activity. Both groups will receive the same general nutritional counseling consisting of general orientations about healthy diets.

**Conclusion:**

The findings from the present study may support public health intervention strategies to improve physical and mental health parameters to be implemented more effectively in this population.

**Clinical trial registration:**

[https://ensaiosclinicos.gov.br/rg/RBR-10yxgcr9/], identifier [U1111-1263-0153].

## Introduction

Chagas disease (CD)—a parasitic infection caused by the flagellate protozoan *Trypanosoma cruzi* firstly described by the Brazilian physician Carlos Chagas in Chagas (1) and also known as American trypanosomiasis—is a neglected disease considered a major public health problem mainly for vulnerable populations, affecting endemic regions such as Latin American countries, and also regions such as Europe and the United States in which the prevalence of CD has increased over the last years due migration movements (2, 3).

The clinical course of CD occurs through two distinct phases: acute and chronic (4, 5). The acute phase is characterized mainly by the absence of symptoms or by the presence of mild or non-specific symptoms such as prolonged fever, malaise, headache, and enlarged lymph nodes. Specifically, when vector transmission occurs, entrance signals such as inoculation chagoma (in the skin) and Romaña signal (in the membrane of the ocular mucosa) may be present, but are rare. Mortality during this phase is ∼5% and occurs mainly due to acute myocarditis or meningoencephalitis. After the acute phase, which lasts around 4 to 8 weeks, the chronic phase ensues unless patients are diagnosed and effectively treated during the acute phase. Patients with chronic CD present clinical manifestations that vary from the absence of specific signs and/or symptoms related to the disease to different degrees of impairments in the heart, esophagus, and/or intestine, that may happen isolated or combined (6). Thus, the chronic phase of CD can be categorized into four different forms according to the clinical characteristics as follows: indeterminate, cardiac, digestive, and mixed (cardio-digestive) forms (4, 5).

Approximately 50–70% of individuals with chronic CD present the indeterminate form of the disease, characterized by parasitological and/or serological evidence of *T. cruzi* infection, but absence of major clinical signs and symptoms, normal electrocardiogram, and normal chest, esophagus and colon X-rays (7, 8). However, the indeterminate form can progress to the determinate forms of CD, with an estimated annual progression rate of 1.9% (9, 10). Most infected individuals will remain in the indeterminate form for the rest of their life, with a benign prognosis (4, 11, 12). However, subclinical abnormalities have also been observed in patients with indeterminate form of CD in comparison to those without CD, including early cardiac abnormalities (e.g., greater fibrosis mass) (13, 14), changes in biomarkers of the inflammatory profile and cardiac function (15–17), and alterations of autonomic modulation (18–21). The prognostic value of most of these early subclinical abnormalities is still not determined, although a recent study that evaluated parameters derived from two-dimensional strain provided independent predictors progression to Chagas cardiomyopathy in patients with the indeterminate form of CD (10).

Considering that CD is a neglected disease, more prevalent among people with low socioeconomic status, individuals with CD are usually impacted on their personal and professional life since they are frequently labeled as vulnerable and limited in several aspects, making social insertion difficult, and impacting their mental health and physical and psychological domains of quality of life (QoL) (22–25). The decrease in QoL is associated with an increase in the symptoms of depression and worsening of the perception of health (26, 27). Few studies have evaluated impact of CD on mental health, mostly focusing on cardiac and digestive forms, usually excluding participants with indeterminate form of CD. Thus, comprehensive interventions to improve different parameters of the physical and mental health of individuals with indeterminate form of CD are necessary.

Physical exercise has been used as an important well-established tool for prevention and control of numerous chronic-degenerative diseases in different populations (28). Compelling evidence has shown that increasing physical activity levels is associated with lower mortality in individuals with and without diseases (29, 30). Other benefits from physical activity include increase in functional capacity, weight control, improvement in pain, better mental health (including depression and anxiety) and QoL (31, 32), anti-inflammatory profile, oxidative capacity, endothelial function, autonomic modulation, and cardiopulmonary and musculoskeletal capacity (33–35).

However, despite the well-recognized benefits, the adherence to conventional supervised exercise programs is low, with approximately 50% of the individuals interrupting such programs in the first 6 months (36, 37). Numerous strategies have been investigated to improve adherence to regular physical activity and home-based exercise has emerged as a good alternative. Besides being a low-cost strategy, which is a great advantage for individuals with CD due to its low socioeconomic level and reduced access to supervised exercise programs, home-based exercise allows more flexibility in deciding when exercise, facilitating adherence (38, 39). Moreover, the effects of physical exercise in individuals with CD have not been explored consistently in the literature. Some previous studies presented promising results for the improvement of cardiac function, functional capacity and QoL in patients with cardiac form of CD, but studies evaluating the effect of physical exercise on health parameters in individuals with indeterminate form of CD are still scarce in the literature (40–44). The PEDI-CHAGAS study (which stands for “Home-Based Exercise Program in the Indeterminate Form of Chagas Disease” in Portuguese) aims to evaluate the effects of a home-based exercise program on several physical and mental health parameters in individuals with indeterminate form of CD.

## Methods and analysis

### Study design

The PEDI-CHAGAS study is a two-arm (exercise and control) phase 3 superiority randomized clinical trial (trial registration REBEC UTN code U1111-1263-0153) that will be conducted at the Evandro Chagas National Institute of Infectious Diseases (INI- Fiocruz, Rio de Janeiro, Brazil). The INI-Fiocruz is a national reference center for treatment and research of infectious diseases and tropical medicine in Brazil that offers comprehensive care for individuals with CD through diagnosis, treatment, and rehabilitation services provided by a multidisciplinary team including physicians, nurses, nutritionists, social assistants, psychologists, pharmacists, physical therapists, physical education professionals, and health technicians. Approximately 900 patients with CD are currently under follow-up at the INI-Fiocruz clinic, of those about 300 have the indeterminate form of CD (45).

### Participants and recruitment

Patients with indeterminate form of CD, both sexes, will be consecutively invited by their assistant physicians to participate in the study. If they agree to participate, informed consent will be obtained, and visits will be scheduled in order to explain the procedures related to the research, and perform the evaluations of the variables that will be investigated in the study.

The inclusion criteria are: (1) serological positivity for CD by at least two different methods (ELISA, indirect immunofluorescence, immunochromatography, or chemiluminescence); and (2) having indeterminate CD defined by the absence of symptoms and/or signs of cardiac CD (normal or presence of non-specific CD abnormalities in ECG) and/or digestive CD. Clinical signs of cardiac CD will be assessed by ECG and echocardiographic (ECHO) evaluation. The ECG abnormalities compatible with cardiac CD are 2nd- and 3rd-degree right bundle-branch block, associated or not to left anterior fascicular block, frequent ventricular premature beats (VPBs > 1 by ECG), polymorphous or repetitive non-sustained ventricular tachycardia, 2nd- and 3rd-degree atrioventricular block, sinus bradycardia with heart rate less than 50 bpm, sinus node dysfunction, 2nd- and 3rd-degree left bundle-branch block, atrial fibrillation, electrical inactive area, or primary ST-T wave changes. ECHO abnormalities related to cardiac CD depends on the stage of the disease and may present as segmental left ventricular (LV) wall motion abnormalities (hypokinesia, dyskinesia, akinesia, small, or large aneurysms), LV dilatation, or global LV systolic dysfunction with diffuse hypokinesia (8). In terms of digestive presentation, patients without symptoms compatible with megacolon or megaesophagus will be considered without digestive CD and will not undergo contrasted examinations of the esophagus and colon.

The exclusion criteria are: (1) <18 years old; (2) evidence of non-CD cardiomyopathy; (3) musculoskeletal or cognitive limitations precluding physical exercise; (4) clinical contraindication for regular exercise; (5) regular physical exercise (1 × per week) in the previous 2 months; and (6) pregnancy.

### Outcomes

Participants will be assessed at baseline, and after three and 6 months of follow-up for all variables, except for echocardiogram exam and fasting blood tests that will be performed at baseline and after 6 months of follow-up. Two clinic visits within a 4-week period for each time point will be performed. The first clinic visit will consist of a clinical interview and physical examination, echocardiogram exam, fatigability evaluation, and cardiopulmonary exercise test. The second clinic visit will include fasting blood tests, nutritional interview, physical fitness evaluation, autonomic modulation assessment, and questionnaires to evaluate back pain, QoL, depression and anxiety, and sleep quality.

The primary outcome will be QoL. Secondary outcomes will include blood pressure, physical fitness components, nutritional status, fatigability, autonomic function, cardiac morphology and function, low back pain, depression and anxiety, stress, sleep quality, medication use and adherence, and biochemical, inflammatory, and cardiac biomarkers.

#### Quality of life

Quality of life will be evaluated using the WHOQOL-Bref questionnaire, a generic instrument developed by the World Health Organization and validated for use in the Brazilian population (46). The questionnaire consisted of 26 questions, two items relate to general QoL (overall domain) and the other 24 encompassed in four domains (physical, psychological, social relationships and environment). Each domain is evaluated by scores ranging from 1 to 5, in which mean scores indicates the individual’s perception of each domain, where a higher score indicates a more positive perception.

#### Blood pressure

Blood pressure measurements will be taken on the left arm with participants seated in a quiet room after 5 min rest using an Omron digital sphygmomanometer. The mean value between three measurements taken at 1-min interval will be considered (47).

#### Physical fitness components

##### Cardiorespiratory capacity

Cardiorespiratory capacity will be assessed by cardiopulmonary exercise test (CPET). The test will be performed in a temperature-controlled environment (18–2°C) using the VO2000 gas analyzer (MedGraphics^®^, St. Paul, MS, USA) with a computerized system Ergo PC Elite (Micromed^®^, Brasília, Brazil) and a treadmill (Inbramed^®^, Porto Alegre, Brazil). The 12-lead electrocardiogram will be recorded in addition to the sampling of the inhaled and expired gases breath by breath and averaged every 10 s. A blinded evaluator will perform an incremental exercise test using a ramp protocol, tailored to achieve a fatigue limited exercise duration of approximately 8–12 min (48). The exercise test workloads will be settled based on age, gender, height, and weight and adapted according to each individual physical condition and effort tolerance. The exam will be limited by symptoms using the Borg perceived effort scale in a range from 6 (no exertion at all) to 20 (maximum effort tolerated), seeking to achieve a respiratory quotient (R) reflecting the relationship between VCO_2_/VO_2_ greater than 1.10 (theoretical maximum effort). The examiner may interrupt the test in case of identification of any harmful clinical or hemodynamic response during the test. The recovery phase will be active with walking in a pre-determined velocity of 2 km.h^–1^ and 2% of inclination. The peak oxygen consumption (VO_2_ peak), which corresponds to the highest volume of oxygen extracted from the air inspired by pulmonary ventilation in a given period of time, calculated as the difference between the inspired and expired O_2_ volume (mL/kg/min) (48), will be evaluated. Other CPET variables including maximum heart rate (HR max), maximum blood pressure (BP max), oxygen consumption at anaerobic threshold (VO_2_ AT), oxygen pulse (O_2_ pulse), ventilation slope equivalent to carbon dioxide production (slope VE/VCO_2_), functional aerobic impairment (FAI), and oxygen uptake efficiency slope (OUES) values will also be evaluated.

##### Flexibility

Flexibility will be assessed using the seat and reach test. The measurement will be obtained with the participant seated on the floor with legs extended. A yardstick is placed on the floor and a tape is placed across it at a right angle to the 15 cm in mark. The participant sits with the yardstick between the legs, with legs extended at right angles to the taped line on the floor. Heels of the feet should touch the edge of the taped line and be about 10–12 cm apart. With the heels positioned on the mark, the participant should bend the trunk to reach as far as possible, holding this position approximately 2 s. The result will be the greatest distance reached with the fingertips in 3 attempts (49).

##### Peripheral muscle strength

Peripheral muscle strength will be assessed using manual handgrip dynamometry with analog hydraulic dynamometer JAMAR^®^, Asimow Engineering^®^, USA. The individual should remain in a seated position, and comfortably holding the dynamometer in the dominant hand, with shoulder in neutral rotation, the elbow flexed at 90°, and the forearm in neutral position. The individual should tighten the equipment with the greatest possible strength, being allowed to move only wrist and finger joints. The highest value of three consecutive attempts interspaced by 1 min will be considered (49).

##### Nutritional status

Nutritional status will be assessed by means of anthropometric measurements (body mass, height and waist circumference) and body composition. The body mass will be measured using a digital scale of the brand Filizola^®^, with a variation of 0.1 kg and a maximum capacity of 150 kg. Participants will be asked to wear light clothes and, at the time of measurement, to remove all belongings that can influence the measurement. Height will be double measured using a stadiometer coupled to the balance, allowing a maximum variation of 0.5 cm between measurements. Participants will be measured in an upright position, without shoes and hair accessories, with parallel feet and ankles joined. The body mass index (BMI) will be calculated by the following formula: BMI = body mass (in kg) divided by squared height (in meters). The waist circumference will be measured by the smallest circumference between the last costal arch and the iliac crest using an inextensible anthropometric tape. To take the measurements, the participant will remain standing, erect, with arms extended along the body and feet together being shirtless or with clothes apart, leaving free the area of the waist. Double measurement will be performed and a difference of up to 1 cm will be accepted, using the mean value between the two measures (50).

Body fat percentage will be measured using a bioelectrical impedance analyzer (Biodynamics^®^ 450, São Paulo, Brazil). The electrodes will be positioned at the prominences of the radius and ulna on the posterior surface of the right wrist and between the tibial malleolus and fibula of the right ankle anterior surface. Participants will be advised to wear light clothes without shoes or socks, to maintain at least 4 h fasting before the exam, to not consume caffeine and alcohol during the previous 24 h before the exam, to not exercise within 8 h before the test, and to empty their bladder 30 min before the exam. Patients will lie in a supine position, with arms relaxed along the body and legs straight and apart. Fat-free mass (FFM) and body fat (BF) will be calculated from impedance (resistance and reactance) and anthropometry (body weight and height) data (51, 52).

#### Fatigability

Fatigability is a relatively new concept that assesses perceived effort after the execution of a standardized task (53, 54). Fatigability will be assessed immediately after a 5-min standardized treadmill walk at a speed of 2.4 km/h (0.67 m/s), without inclination. Participants will rate their perceived effort using the Borg perceived effort scale, which ranges from 6 (no exertion at all) to 20 (maximal exertion), with Borg >10 being considered high fatigability (53).

#### Autonomic modulation

Autonomic modulation will be assessed through 30:15 ratio test and heart rate variability analysis using an electrocardiogram and heart rate monitor (Polar V800^®^, PolarTM Electro, Kempele, Finland). The protocol consists of staying 15 min lying in a supine position, then change this posture to a standing position. After standing, the relationship between heart rates (RR intervals) corresponding to maximum tachycardia (around the 15th beat) and maximum bradycardia (around the 30th beat) will be evaluated (55). The RR intervals obtained during the recording of the electrocardiogram and obtained by the heart rate monitor will be used for the analysis of heart rate variability using specific software (Kubios HRV v. 2.2, University of Kuopio, Kuopio, Finland). The heart rate variability indices that will be analyzed in the frequency domain (FFT method) are very low frequency component (VLF), low frequency component (LF), high frequency component (HF) and sympathovagal index (LF/HF). In the time domain, the indices will be mean normal RR intervals (RRi), standard deviation of normal NN intervals (SDNN), square root of the mean squared successive differences from adjacent RRi (rMSSD), and percentage number of pairs of adjacent RRi differing by more than 50 ms from previous RRi (pNN50). SDNN, rMSSD, and pNN50 are acknowledged to reflect the parasympathetic modulation within short periods. Spectral analysis will be performed by the Fast Fourier transform method, with frequency bands corresponding to low-frequency (LF: 0.04–0.15 Hz), high-frequency (HF: 0.15–0.40 Hz), and total power (TP; meaning LF plus HF). The LF and HF reflect a predominance of combined sympathetic-parasympathetic and isolated parasympathetic modulation, respectively, while LF: HF ratio will be adopted as a marker of sympathovagal balance (56).

#### Cardiac morphology and function

The two-dimensional Doppler echocardiogram will be performed to evaluate cardiac function and morphology. The images will be acquired with Epic Cvx equipment (Philips, Andover, MA, USA). The images will be obtained in the LV standard sections: left parasternal sections in the long and short axes (basal, middle and apical), and apical sections in 3-, 4-, and 2- chambers. Cardiac dimensions will be measured according to international recommendations (57). The ejection fraction (EF) and the final diastolic and systolic volumes of the LV will be determined by the Simpson method. Mitral flow will be obtained in the 4-chamber window and its maximum values will be determined in the rapid filling phase (E) and atrial contraction (A), the E/A ratio, and the deceleration time of the E wave. The pulmonary artery systolic pressure will be evaluated by the systolic pressure gradient between the right ventricle (RV) and the right atrium (AD), obtained through the analysis of the tricuspid insufficiency, added to the estimate of the AD pressure (58). The function of the RV will be evaluated at the apical 4-chamber cut. The maximum velocity of myocardial displacement during systole (S’VD) and displacement of the tricuspid ring by the M-mode to the 4-chamber apical cut will be measured. Tissue Doppler will be obtained in the mitral ring in its septal and lateral segments. The maximum velocity of myocardial displacement during systole, at the beginning and end of diastole will be determined. The value of each of these components will be the mean of the respective septal and lateral values.

#### Low back pain

The presence of low back pain in the previous year will be evaluated by the question: “In the past year, did you have pain in your low back?” with the answer options “Yes,” “No,” and “I don’t know.” Among those who answer “Yes” for the previous question, the intensity (0–10 scale, 0 = no pain, 10 = extremely intense pain) and duration [longest consecutive time period (weeks)] of low back pain will be evaluated (59). The Brazilian version of the Roland Morris functionality questionnaire, which includes 24 items (yes or no) on physical disability caused by back pain, will also be applied. The sum of the answers generates a score ranging from 0 (no disability) to 24 (severe disability) (60).

#### Depression and anxiety

Evaluation of depressive symptoms will be performed using Beck Depression Inventory, which consists of an original scale of 21 items, including symptoms and attitudes, whose intensity varies from 0 to 3. Items refer to sadness, pessimism, feeling of failure, lack of satisfaction, sense of guilt, sense of punishment, self-blame, self-accusation, suicidal ideas, bouts of crying, irritability, social withdrawal, indecision, distortion of body image, inhibition to work, sleep disorder, fatigue, loss of appetite, weight loss, somatic concern, decreased libido. The adequacy of the Brazilian version for clinical use was evidenced in the discriminant validity study, in which significant differences of score were obtained in different samples of anxious and depressed patients clinically diagnosed (61). Higher values indicate greater severity of depressive symptoms.

Anxiety will be assessed through the Brazilian version of Beck Anxiety Inventory, which is one of the most widely used instruments to assess anxiety symptoms in clinical and research contexts. Similarly, to the depression inventory, it consists of 21 items that cover the most frequent symptoms of anxiety, with each item scoring from 0 to 3 and the higher the score, the more severe the symptoms (62).

#### Stress

Stress will be evaluated using the Brazilian validated perception stress scale with 10 items (63). The PSS-10 is a general scale in which individuals rate the frequency of their feelings and thoughts related to events and situations that occurred in the last month. Six items are negative (items 1, 2, 3, 6, 9, 10) and the remaining four are positive (items 4, 5, 7, 8). There are five options of answers that ranges from “never” (0) to “often” (4). The four positive items are scored inversely. The final score is obtained summing up the scores from all items, with higher scores corresponding to greater stress.

#### Sleep quality

Sleep quality will be assessed using the Brazilian version of Pittsburgh Sleep Quality Index (64). This questionnaire is composed of 19 questions divided into 7 components (subjective quality of sleep, sleep latency, duration of sleep, habitual sleep efficiency, sleep changes, use of sleeping drugs, and diurnal sleep dysfunction). Each component has a score that ranges from 0 to 3 and an overall score that ranges from 0 to 21 according to the sum of each component. Higher scores indicate worse sleep quality. A cutoff point greater than 5 has been used to indicate poor sleep quality.

#### Medication use, adherence, and adverse drug events

Information on quantity and dosage of the medications regularly used will be obtained from patient’s medical records using the closest medical appointment to the study assessment. Medication adherence will be assessed using the Brazilian version of Medication Adherence Rating Scale (MARS). This questionnaire comprised 10 dichotomous questions (yes/no) to assess intentional (“I avoid using it if I can”) and non-intentional medication adherence (“I forget to use it”). The answers are scored as zero (non-adherence) or one (adherence) and a global score is obtained by summing the items values, so that the result ranges from 0 (low probability of adherence) to 10 (high probability of adherence) (65, 66). The incidence, cause, and intensity of adverse drug events (ADEs) will be determined during the study follow-up based on the information contained in medical records. The Naranjo algorithm and intensity classification will be applied to determine the cause and intensity of ADEs (67).

#### Biochemical, inflammatory, and cardiac biomarkers

Blood drawn will be performed at the INI-Fiocruz laboratory, after at least 10-h overnight fast. Complete blood counts, lipid profile, glucose, insulin, urea, creatinine, lactate, uric acid, glycated hemoglobin, sodium, potassium, chlorine, calcium and magnesium will be evaluated. The inflammatory markers that will be analyzed are C-reactive protein, tumor necrosis factor, interleukin β-1, interleukin-6, interleukin-10, interleukin-17, MCP-1, MMP-2, MMP-8, MMP-9, PIIINP, CTx, sST2. Biomarkers of cardiac function and fibrosis such as BNP, troponin and galactin-3 will also be evaluated.

The plasma lipids will be measured using Kit Gold analyzes, the exception of LDL and VLDL cholesterol that will be estimated by the Friedwald formula (68). Plasma glucose will be determined by enzymatic-colorimetric assay (Kit Gold analyzes), using Konelab 6.0.1, with automated reading at a wavelength of 500 nm. Plasma insulin will be determined by radioimmunoassay (Immuchem Kit 125/RIA). The following formula will be used to calculate HOMA-IR: HOMA-IR = fasting glucose (mmol/l) X insulin (μU/ml)/22.5. Inflammatory and cardiac biomarkers will be dosed using specific kits using the Elisa method. For analysis of biomarkers that are not included in the INI/FIOCRUZ exam routine (inflammatory and cardiac biomarkers), the blood samples will be centrifuged and then stored at −70°C until the time of laboratory analysis, remaining stored under the responsibility of the principal investigator of this research project (MFFM).

### Participant timeline

**FIGURE 1:**
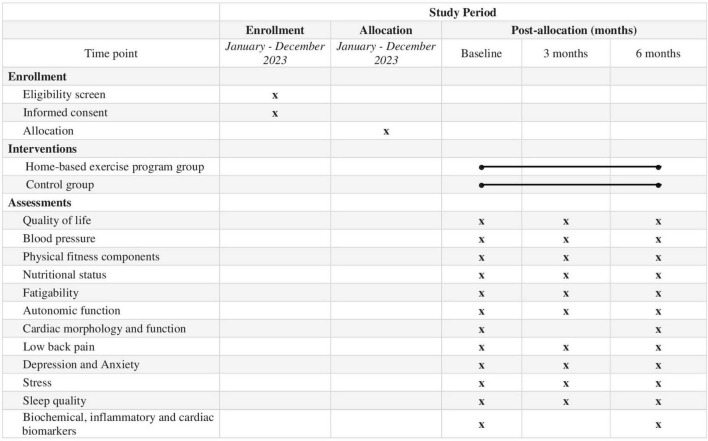
SPIRIT schedule of enrolment, interventions, and assessments of the study.

Time schedule of enrollment, interventions, assessments and visits for participants is presented in Figure 1.

### Masking

Since home-based exercise implies a behavioral intervention, it is not feasible to mask the patients. However, the evaluators and data analyst will be masked. A blinded researcher will perform all data analysis.

### Randomization

A sequence will be generated in a specific program (Winpepi version 11.61) to randomly allocate participants between the two groups at a ratio of 1:1. The sequence will be generated in blocks and strata of sex and age (<60 years) by a single researcher who will not be involved in recruitment. Individual, sealed, opaque and translucent envelopes will be used during the randomization procedure for sequential allocation to intervention or control groups. Block size will be masked from the investigators involved in recruitment.

### Intervention

Participants in the intervention group will undergo a home-based exercise program whilst those in the control group will receive only general information regarding the benefits of physical activity. The intervention group will receive a booklet with routines of physical exercises (Supplementary Appendix 1) to be performed at home three times a week, 60 min per session. The physical exercise program will be divided into three parts: (1) warm-up (5 min): stretching exercises and joint mobilization; (2) aerobic activities (20 to 40 min): circuit exercises involving large arm and legs muscle groups, as well as exercises using ball, calisthenics exercises and standing up from a chair (balls will be provided); (3) cool-down (5 min): the same stretching exercises from the warming-up period. The participants will be encouraged to perform aerobic activities with moderate intensity, characterized by a mild breathing discomfort that allows talking during the exercise (69). In the first week, a duration of approximately 20 min for aerobic activities will be recommended, with a 10 min weekly increments, until the duration of 60 min for the overall training session is reached. Patients will be advised to reduce training intensity if they experience any discomfort during exercise such as shortness of breath and/or muscle pain. The exercise routine will be modified after 3 months of follow-up, when a booklet with new exercises will be delivered to patients (Supplementary Appendix 2). For participants that have a mobile phone, messages with animated GIFs will also be send to facilitate the understanding of the exercises to be performed.^1^

Adherence to the exercise protocol will be verified by recording the activity at the end of exercise booklet and during the periodic visits to INI-Fiocruz. Additionally, the level of physical activity will be assessed using triaxial accelerometers (Actical, Phillips, Respironics, OR, USA) at baseline and after 6 months of follow-up (70). The accelerometer will be positioned on the non-dominant wrist using elastic wristbands, which must be used for 7 days without interruption, except for water activities and while bathing. Participants will be instructed to maintain their routine (71).

Both groups (intervention and control) will receive the same general nutritional counseling consisting of general orientations about healthy eating habits, such as reducing saturated fat and increasing poly- and monounsaturated fatty acids consumption, increasing water, vitamins and high-fiber carbohydrates consumption, reducing the sodium intake. Nutritional counseling will take place during the assessment visits at baseline, after three and 6 months of follow-up. Compliance to nutritional counseling will be checked using a 24-h recall at baseline and after 6 months of follow-up, consisting on the identification and quantification of all food and beverages consumed in the day before the interview (72). The consumption of nutrients will be calculated using DietWin Professional Version 2008 software.

### Sample size

The sample size was calculated based on the findings by Carta et al. (73), which evaluated the effect of a physical exercise program on the QoL. In this study, there was a difference of 2.2 (± 2.1) points for the intervention group and 0.0 (± 3.2) point for the control group in the physical domain of the WHOQOL-Bref questionnaire (46). Assuming α = 0.05 and β = 0.10 and inflating the sample by 20% for eventual losses to follow-up, a total of 80 individuals will be necessary to perform the present study (40 in each group).

### Interim analysis and stopping rules

Two interim analyses are planned. The first will be conducted when the 40th participant completes 3 months of follow-up and the second when the last recruited participant completes 3 months of follow-up. Trial interruption for ethical reasons due to either positive or negative results exceeding expectations may be recommended by an independent committee. The prespecified stopping rules are >100% difference between group in any outcome, serious adverse events twice as frequent in one of the groups including death, Chagas cardiomyopathy progression, acute myocardial infarction, unstable angina, cardiac arrest, incident HF, and stroke. All these estimates should have a significance level <0.01 in any of the interim analyses.

### Statistical analysis

A dataset will be prepared including the information from all patients using the Redcap platform. Statistical analysis will be performed using Stata statistical software (version 13.0). The data analysis will comprise the estimation of the mean (standard deviations) for continuous and absolute frequency (percentages) for categorical variables. The effect of the home-based exercise program on the continuous variables over the intervention period will be determined using a linear mixed model. For categorical variables, generalized estimated equations with log link and binomial distribution will be performed to evaluate changes in the proportion of individuals between groups over the time. All measures performed in the intervention and control group will be used, regardless of losses to follow-up or compliance to the protocol during the intervention period, characterizing an intention-to-treat analysis. The variable of interest will be the interaction between time × treatment, which estimates the change rate of the outcome over time. Protocol analysis including only completers will also be performed. Adjustments for baseline prognostic variables will be made in case of unbalance distributions between the groups. A *p*-value < 0.05 will be considered significant for all statistical tests.

### Ethical aspects

The present research was approved by the Institutional Review Board of the Evandro Chagas National Institute of Infectious Disease (approved on March 19, 2021 under number 4.629.748). The researchers involved in the present work are committed to maintain the confidentiality and privacy of the data obtained. The results obtained in this work will be published in scientific communications, maintaining the anonymity of the participants. There are no conflicts of interest involved in this work on the part of the researcher or collaborators, nor any restriction regarding the public disclosure of the results, which will occur whether they are favorable or not to the researcher’s hypothesis.

To be included, all patients must sign an informed consent form that will follow the good clinical practice standards. Patients will be instructed to read (or listen, in cases where the patient has difficulty reading) the consent form, without time restrictions, and will receive explanations for any doubts regarding the objectives and procedures to be adopted during the procedure. All participants will be informed that participation is voluntary, and that refusal to participate will not result in any harm to their treatment in the institution. They will be also advised that they can withdraw from participating in the study at any time.

At the end of the study, participation in the exercise program will be offered to individuals allocated to the control group if the results indicate an important effect of exercise in improving the investigated parameters.

## Results

**FIGURE 2:**
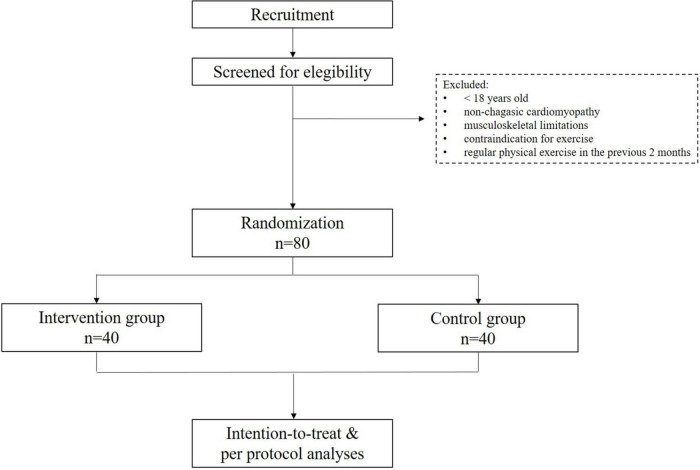
Planned flow diagram of the study.

The planned flow diagram is presented in Figure 2. The trial recruitment is planned to start in January 2023, and data collection is expected to be concluded in December 2024. The trial findings will be presented in accordance with the CONSORT (Consolidated Standards of Reporting Trials) reporting guidelines. No results are available as of manuscript preparation.

## Discussion

Previous studies demonstrated that individuals with the indeterminate form of CD, despite its good prognosis, already have some subclinical abnormalities in comparison to non-infected individuals. CD is a neglected disease associated with low socioeconomic levels that may negatively impact on several aspects of mental health and QoL. In addition, due to the change in the epidemiological profile of CD population, many individuals have changed their lifestyles, with greater intake of inappropriate foods, decreased physical activity levels, greater exposure to stress and worsening of sleep quality, which in turn are associated with a higher incidence of numerous chronic diseases. Taken together, such changes can negatively impact the prognosis of these patients and decrease their QoL (74–76).

In this sense, regular physical exercise has been proposed as an important intervention strategy in the treatment of numerous chronic diseases, in addition to contributing to the improvement of several parameters of the physical and mental health (77, 78). However, studies evaluating the effects of a physical exercise program in individuals with the indeterminate form of CD were not found in the literature. Thus, it is urgently needed to evaluate the effects of a physical exercise program in individuals with the indeterminate form of CD. From the knowledge generated by the present study, public health intervention strategies aiming to improve physical and mental health parameters may be implemented more effectively in this population.

## Ethics statement

The studies involving human participants were reviewed and approved by the Evandro Chagas National Institute of Infectious Disease Ethics Commitee. The patients/participants provided their written informed consent to participate in this study.

## Author contributions

LGR and MM contributed to the study design and drafted the manuscript. All authors revised and approved the final version and agreed with all aspects of the work in ensuring that questions related to the accuracy and integrity of any part of the work are appropriately investigated and resolved.

## Footnotes

1
https://osf.io/frqh4/

## References

[1] Chagas C. Nova tripanozomiaze humana: estudos sobre a morfolojia e o ciclo evolutivo do *Schizotrypanum cruzi* n. gen., n. sp., ajente etiolojico de nova entidade morbida do homem. *Mem Inst Oswaldo Cruz*. (1909) 1:159–218.

[2] Pinto Dias J. Human chagas disease and migration in the context of globalization: some particular aspects. *J Trop Med.* (2013) 2013:789758. 10.1155/2013/789758 23606862 PMC3625591

[3] Lidani K Andrade F Bavia L Damasceno F Beltrame M Messias-Reason I Chagas disease: from discovery to a worldwide health problem. *Front Public Health.* (2019) 7:166. 10.3389/fpubh.2019.00166 31312626 PMC6614205

[4] Perez-Molina J Molina I. Chagas disease. *Lancet.* (2018) 391:82–94. 10.1016/S0140-6736(17)31612-4 28673423

[5] Echeverria L Morillo C. American trypanosomiasis (Chagas Disease). *Infect Dis Clin North Am.* (2019) 33:119–34. 10.1016/j.idc.2018.10.015 30712757

[6] Nunes M Beaton A Acquatella H Bern C Bolger A Echeverria L Chagas cardiomyopathy: an update of current clinical knowledge and management: a scientific statement from the American heart association. *Circulation.* (2018) 138:e169–209. 10.1161/CIR.0000000000000599 30354432

[7] Dias J Ramos A Jr. Gontijo ED Luquetti A Shikanai-Yasuda M Coura J 2. Nd Brazilian consensus on chagas disease, 2015. *Rev Soc Bras Med Trop.* (2016) 49 (Suppl. 1):3–60. 10.1590/0037-8682-0505-2016 27982292

[8] Saraiva R Mediano M Mendes F Sperandio da Silva G Veloso H Sangenis L Chagas heart disease: an overview of diagnosis, manifestations, treatment, and care. *World J Cardiol.* (2021) 13:654–75. 10.4330/wjc.v13.i12.654 35070110 PMC8716970

[9] Chadalawada S Sillau S Archuleta S Mundo W Bandali M Parra-Henao G Risk of chronic cardiomyopathy among patients with the acute phase or indeterminate form of chagas disease: a systematic review and meta-analysis. *JAMA Netw Open.* (2020) 3:e2015072. 10.1001/jamanetworkopen.2020.15072 32865573 PMC7489816

[10] Saraiva R Mediano M Quintana M Sperandio da Silva G Costa A Sousa A Two-dimensional strain derived parameters provide independent predictors of progression to chagas cardiomyopathy and mortality in patients with chagas disease. *Int J Cardiol Heart Vasc.* (2022) 38:100955. 10.1016/j.ijcha.2022.100955 35169612 PMC8826593

[11] Maguire J Hoff R Sherlock I Guimaraes A Sleigh A Ramos N Cardiac morbidity and mortality due to chagas’ disease: prospective electrocardiographic study of a Brazilian community. *Circulation.* (1987) 75:1140–5. 10.1161/01.cir.75.6.1140 3552307

[12] Dias J Ramos A Jr. Gontijo E Luquetti A Shikanai-Yasuda M Coura J [Brazilian consensus on chagas disease, 2015]. *Epidemiol Serv Saude.* (2016) 25:7–86. 10.5123/S1679-49742016000500002 27869914

[13] Noya-Rabelo M Macedo C Larocca T Machado A Pacheco T Torreao J The presence and extension of myocardial fibrosis in the undetermined form of chagas’ disease: a study using magnetic resonance. *Arq Bras Cardiol.* (2018) 110:124–31. 10.5935/abc.20180016 29466491 PMC5855905

[14] Pinheiro M Moll-Bernardes R Camargo G Siqueira F Azevedo C Holanda M Associations between cardiac magnetic resonance T1 mapping parameters and ventricular arrhythmia in patients with chagas disease. *Am J Trop Med Hyg.* (2020) 103:745–51. 10.4269/ajtmh.20-0122 32431281 PMC7410430

[15] Lopez L Arai K Gimenez E Jimenez M Pascuzo C Rodriguez-Bonfante C [C-Reactive protein and interleukin-6 serum levels increase as chagas disease progresses towards cardiac failure]. *Rev Esp Cardiol.* (2006) 59:50–6. 16434004

[16] Sousa G Gomes J Fares R Damasio M Chaves A Ferreira K Plasma cytokine expression is associated with cardiac morbidity in chagas disease. *PLoS One.* (2014) 9:e87082. 10.1371/journal.pone.0087082 24603474 PMC3945957

[17] Medeiros N Gomes J Fiuza J Sousa G Almeida E Novaes R Mmp-2 and Mmp-9 plasma levels are potential biomarkers for indeterminate and cardiac clinical forms progression in chronic chagas disease. *Sci Rep.* (2019) 9:14170. 10.1038/s41598-019-50791-z 31578449 PMC6775161

[18] Rocha A Lombardi F da Costa Rocha M Barros M Val Barros Vda C Reis A Chronotropic incompetence and abnormal autonomic modulation in ambulatory chagas disease patients. *Ann Noninvasive Electrocardiol.* (2006) 11:3–11. 10.1111/j.1542-474X.2006.00054.x 16472276 PMC6932442

[19] Llaguno M Pertili L da Silva M Bunazar P Reges A Faleiros A The relationship between heart rate variability and serum cytokines in chronic chagasic patients with persistent parasitemia. *Pacing Clin Electrophysiol.* (2011) 34:724–35. 10.1111/j.1540-8159.2010.03025.x 21276024

[20] Barbosa-Ferreira J Mady C Ianni B Lopes H Ramires F Salemi V Dysregulation of autonomic nervous system in chagas’ heart disease is associated with altered adipocytokines levels. *PLoS One.* (2015) 10:e0131447. 10.1371/journal.pone.0131447 26147101 PMC4493107

[21] Costa H Lima M Costa F Chaves A Nunes M Figueiredo P Reduced functional capacity in patients with chagas disease: a systematic review with meta-analysis. *Rev Soc Bras Med Trop.* (2018) 51:421–6. 10.1590/0037-8682-0158-2018 30133623

[22] Guariento M Camilo M Camargo A. Working conditions of chagas’ disease patients in a large Brazilian city. *Cad Saude Publica.* (1999) 15:381–6. 10.1590/s0102-311x1999000200022 10409790

[23] Ballester-Gil L Stotz E Hasslocher-Moreno A de Azevedo B de Araujo-Jorge T. [The knowledge of chagasic patients about their disease: collective construction of a research instrument and test of its applicability]. *Cien Saude Colet.* (2008) 13(Suppl. 2):2199–214. 10.1590/s1413-81232008000900025 19039404

[24] Ventura-Garcia L Roura M Pell C Posada E Gascon J Aldasoro E Socio-cultural aspects of chagas disease: a systematic review of qualitative research. *PLoS Negl Trop Dis.* (2013) 7:e2410. 10.1371/journal.pntd.0002410 24069473 PMC3772024

[25] Sousa G Costa H Souza A Nunes M Lima M Rocha M. Health-related quality of life in patients with chagas disease: a review of the evidence. *Rev Soc Bras Med Trop.* (2015) 48:121–8. 10.1590/0037-8682-0244-2014 25992924

[26] Ozaki Y Guariento M de Almeida E. Quality of life and depressive symptoms in chagas disease patients. *Qual Life Res.* (2011) 20:133–8. 10.1007/s11136-010-9726-1 21046258

[27] Cavalcanti M Nascimento E Alchieri J Andrade C. Manifestations and strategies of coping with Chagas disease that interfere in the quality of life of the individual: a systematic review. *Cien Saude Colet.* (2019) 24:1405–16. 10.1590/1413-81232018243.11842017 31066842

[28] Zhao M Veeranki S Magnussen C Xi B. Recommended physical activity and all cause and cause specific mortality in us adults: prospective cohort study. *BMJ.* (2020) 370:m2031. 10.1136/bmj.m2031 32611588 PMC7328465

[29] Pedersen B Saltin B. Exercise as medicine - evidence for prescribing exercise as therapy in 26 different chronic diseases. *Scand J Med Sci Sports.* (2015) 25(Suppl. 3):1–72. 10.1111/sms.12581 26606383

[30] Jeong S Kim S Kang S Kim H Yoon C Youn T Mortality reduction with physical activity in patients with and without cardiovascular disease. *Eur Heart J.* (2019) 40:3547–55. 10.1093/eurheartj/ehz564 31504416 PMC6855138

[31] Garber C Blissmer B Deschenes M Franklin B Lamonte M Lee I American college of sports medicine position stand. Quantity and quality of exercise for developing and maintaining cardiorespiratory, musculoskeletal, and neuromotor fitness in apparently healthy adults: guidance for prescribing exercise. *Med Sci Sports Exerc.* (2011) 43:1334–59. 10.1249/MSS.0b013e318213fefb 21694556

[32] Piercy K Troiano R Ballard R Carlson S Fulton J Galuska D The physical activity guidelines for Americans. *JAMA.* (2018) 320:2020–8. 10.1001/jama.2018.14854 30418471 PMC9582631

[33] Matta Mello Portugal E Cevada T Sobral Monteiro-Junior R Teixeira Guimaraes T da Cruz Rubini E Lattari E Neuroscience of exercise: from neurobiology mechanisms to mental health. *Neuropsychobiology.* (2013) 68:1–14. 10.1159/000350946 23774826

[34] Herdy A Lopez-Jimenez F Terzic C Milani M Stein R Carvalho T South American guidelines for cardiovascular disease prevention and rehabilitation. *Arq Bras Cardiol.* (2014) 103(2 Suppl. 1):1–31. 10.5935/abc.2014s003 25387466

[35] Pinckard K Baskin K Stanford K. Effects of exercise to improve cardiovascular health. *Front Cardiovasc Med.* (2019) 6:69. 10.3389/fcvm.2019.00069 31214598 PMC6557987

[36] Rhodes R Fiala B Conner M. A review and meta-analysis of affective judgments and physical activity in adult populations. *Ann Behav Med.* (2009) 38:180–204. 10.1007/s12160-009-9147-y 20082164

[37] Turk-Adawi K Grace S. Narrative review comparing the benefits of and participation in cardiac rehabilitation in high-, middle- and low-income countries. *Heart Lung Circ.* (2015) 24:510–20. 10.1016/j.hlc.2014.11.013 25534902 PMC4527841

[38] Kahn E Ramsey L Brownson R Heath G Howze E Powell K The effectiveness of interventions to increase physical activity. A systematic review. *Am J Prev Med.* (2002) 22(Suppl. 4):73–107. 10.1016/s0749-3797(02)00434-8 11985936

[39] Thomas R Beatty A Beckie T Brewer L Brown T Forman D Home-based cardiac rehabilitation: a scientific statement from the American association of cardiovascular and pulmonary rehabilitation, the American heart association, and the American college of cardiology. *Circulation.* (2019) 140:e69–89. 10.1161/CIR.0000000000000663 31082266

[40] Lima M Rocha M Nunes M Sousa L Costa H Alencar M A randomized trial of the effects of exercise training in chagas cardiomyopathy. *Eur J Heart Fail.* (2010) 12:866–73. 10.1093/eurjhf/hfq123 20675669

[41] Fialho P Tura B Sousa A Oliveira C Soares C Oliveira J Effects of an exercise program on the functional capacity of patients with chronic Chagas’ heart disease, evaluated by cardiopulmonary testing. *Rev Soc Bras Med Trop.* (2012) 45:220–4. 10.1590/s0037-86822012000200016 22534996

[42] Mediano M Mendes Fde S Pinto V Silva G Silva P Carneiro F Cardiac rehabilitation program in patients with Chagas heart failure: a single-arm pilot study. *Rev Soc Bras Med Trop.* (2016) 49:319–28. 10.1590/0037-8682-0083-2016 27384829

[43] Mediano M Mendes F Pinto V Silva P Hasslocher-Moreno A Sousa A. Reassessment of quality of life domains in patients with compensated Chagas heart failure after participating in a cardiac rehabilitation program. *Rev Soc Bras Med Trop.* (2017) 50:404–7. 10.1590/0037-8682-0429-2016 28700063

[44] de Souza Nogueira Sardinha Mendes F Mediano M de Castro E da Silva P Carneiro F de Holanda M Effect of physical exercise training in patients with Chagas heart disease (from the Peach Study). *Am J Cardiol.* (2020) 125:1413–20. 10.1016/j.amjcard.2020.01.035 32171439

[45] Portela L Mesquita M Giraldes J Varela M Brasil P Costa A Socio-epidemiological factors and comorbidities associated with Chagas disease manifestations in two urban reference health care centres in Rio De Janeiro, Brazil. *Trans R Soc Trop Med Hyg.* (2022):trac068. 10.1093/trstmh/trac068 [Epub ahead of print]. 35896031

[46] Fleck M Louzada S Xavier M Chachamovich E Vieira G Santos L [Application of the Portuguese version of the abbreviated instrument of quality life whoqol-bref]. *Rev Saude Publica.* (2000) 34:178–83. 10.1590/s0034-89102000000200012 10881154

[47] Jones D. Implementing automated office blood pressure measurement. *Hypertension.* (2019) 74:436–40. 10.1161/HYPERTENSIONAHA.118.10966 31352826 PMC6685739

[48] Balady G Arena R Sietsema K Myers J Coke L Fletcher G Clinician’s guide to cardiopulmonary exercise testing in adults: a scientific statement from the American heart association. *Circulation.* (2010) 122:191–225. 10.1161/CIR.0b013e3181e52e69 20585013

[49] American College of Sports Medicine Thompson W Gordon N Pescatello L. *Acsm’s Guidelines for Exercise Testing and Prescription.* 8th ed. Philadelphia, PA: Lippincott Williams & Wilkins (2010). xxi,380 p.

[50] Norton K Olds T Australian Sports Commission. *Anthropometrica: A Textbook of Body Measurement for Sports and Health Courses.* Sydney, NSW: UNSW Press (1996). xi,413 p.

[51] Kyle U Bosaeus I De Lorenzo A Deurenberg P Elia M Gomez J Bioelectrical impedance analysis–part I: review of principles and methods. *Clin Nutr.* (2004) 23:1226–43. 10.1016/j.clnu.2004.06.004 15380917

[52] Kyle U Bosaeus I De Lorenzo A Deurenberg P Elia M Manuel Gomez J Bioelectrical impedance analysis-part Ii: utilization in clinical practice. *Clin Nutr.* (2004) 23:1430–53. 10.1016/j.clnu.2004.09.012 15556267

[53] Simonsick E Schrack J Glynn N Ferrucci L. Assessing fatigability in mobility-intact older adults. *J Am Geriatr Soc.* (2014) 62:347–51. 10.1111/jgs.12638 24417536 PMC3947865

[54] Wanigatunga A Varadhan R Simonsick E Carlson O Studenski S Ferrucci L Longitudinal relationship between interleukin-6 and perceived fatigability among well-functioning adults in mid-to-late life. *J Gerontol A Biol Sci Med Sci.* (2019) 74:720–5. 10.1093/gerona/gly120 29846512 PMC6941496

[55] Duque A Mediano M De Lorenzo A Rodrigues L Jr. Cardiovascular autonomic neuropathy in diabetes: pathophysiology, clinical assessment and implications. *World J Diabetes.* (2021) 12:855–67. 10.4239/wjd.v12.i6.855 34168733 PMC8192252

[56] Circulation. Heart rate variability: standards of measurement, physiological interpretation and clinical use. Task force of the European society of cardiology and the North American society of pacing and electrophysiology. *Circulation*. (1996) 93:1043–65. 8598068

[57] Lang R Badano L Mor-Avi V Afilalo J Armstrong A Ernande L Recommendations for cardiac chamber quantification by echocardiography in adults: an update from the American society of echocardiography and the European association of cardiovascular imaging. *J Am Soc Echocardiogr.* (2015) 28:1.e–39.e. 10.1016/j.echo.2014.10.003 25559473

[58] Rudski L Lai W Afilalo J Hua L Handschumacher M Chandrasekaran K Guidelines for the echocardiographic assessment of the right heart in adults: a report from the American society of echocardiography endorsed by the European association of echocardiography, a registered branch of the European society of cardiology, and the Canadian society of echocardiography. *J Am Soc Echocardiogr.* (2010) 23:685–713;quiz86–8. 10.1016/j.echo.2010.05.010 20620859

[59] Simonsick E Aronson B Schrack J Hicks G Jerome G Patel K Lumbopelvic pain and threats to walking ability in well-functioning older adults: findings from the Baltimore longitudinal study of aging. *J Am Geriatr Soc.* (2018) 66:714–20. 10.1111/jgs.15280 29411349 PMC5906159

[60] Nusbaum L Natour J Ferraz M Goldenberg J. Translation, adaptation and validation of the Roland-Morris questionnaire–Brazil Roland-Morris. *Braz J Med Biol Res.* (2001) 34:203–10. 10.1590/s0100-879x2001000200007 11175495

[61] Gomes-Oliveira M Gorenstein C Lotufo Neto F Andrade L Wang Y. Validation of the Brazilian Portuguese version of the beck depression inventory-ii in a community sample. *Braz J Psychiatry.* (2012) 34:389–94. 10.1016/j.rbp.2012.03.005 23429809

[62] Cunha J. *Manual Da Versão Em Português Das Escalas Beck.* 1a ed. São Paulo: Casa do Psicólogo (2001). 172 p.

[63] Reis R Hino A Anez C. Perceived stress scale: reliability and validity study in Brazil. *J Health Psychol.* (2010) 15:107–14. 10.1177/1359105309346343 20064889

[64] Bertolazi A Fagondes S Hoff L Dartora E Miozzo I de Barba M Validation of the Brazilian Portuguese version of the Pittsburgh sleep quality index. *Sleep Med.* (2011) 12:70–5. 10.1016/j.sleep.2010.04.020 21145786

[65] Thompson K Kulkarni J Sergejew A. Reliability and validity of a new medication adherence rating scale (Mars) for the psychoses. *Schizophr Res.* (2000) 42:241–7. 10.1016/s0920-9964(99)00130-9 10785582

[66] Moreira I Bandeira M Pollo T Oliveira M. Cross-cultural adaptation to Brazil of medication adherence rating scale for psychiatric patients. *J Bras Psiquiatr.* (2014) 63:273–80. 10.1590/0047-2085000000035

[67] Naranjo C Busto U Sellers E Sandor P Ruiz I Roberts E A method for estimating the probability of adverse drug reactions. *Clin Pharmacol Ther.* (1981) 30:239–45. 10.1038/clpt.1981.154 7249508

[68] Friedewald W Levy R Fredrickson D. Estimation of the concentration of low-density lipoprotein cholesterol in plasma, without use of the preparative ultracentrifuge. *Clin Chem.* (1972) 18:499–502. 4337382

[69] Persinger R Foster C Gibson M Fater D Porcari J. Consistency of the talk test for exercise prescription. *Med Sci Sports Exerc.* (2004) 36:1632–6. 15354048

[70] Heil D. Predicting activity energy expenditure using the actical activity monitor. *Res Q Exerc Sport.* (2006) 77:64–80. 10.1080/02701367.2006.10599333 16646354

[71] Paravidino V Mediano M Crochemore-Silva I da Cruz V Antunes M Beaulieu K The compensatory effect of exercise on physical activity and energy intake in young men with overweight: the efect randomised controlled trial. *Physiol Behav.* (2021) 229:113249. 10.1016/j.physbeh.2020.113249 33221391

[72] Shim J Oh K Kim H. Dietary assessment methods in epidemiologic studies. *Epidemiol Health.* (2014) 36:e2014009. 10.4178/epih/e2014009 25078382 PMC4154347

[73] Carta M Hardoy M Pilu A Sorba M Floris A Mannu F Improving physical quality of life with group physical activity in the adjunctive treatment of major depressive disorder. *Clin Pract Epidemiol Ment Health.* (2008) 4:1. 10.1186/1745-0179-4-1 18221549 PMC2266746

[74] Dias J. [Globalization, inequity and Chagas disease]. *Cad Saude Publica.* (2007) 23(Suppl. 1):S13–22. 10.1590/s0102-311x2007001300003 17308713

[75] Jackson Y Castillo S Hammond P Besson M Brawand-Bron A Urzola D Metabolic, mental health, behavioural and socioeconomic characteristics of migrants with Chagas disease in a non-endemic country. *Trop Med Int Health.* (2012) 17:595–603. 10.1111/j.1365-3156.2012.02965.x 22487303

[76] Navarro E Abreu M Tavares F Corrente J Arruda C Pereira P. Indeterminate form of Chagas’ disease and metabolic syndrome: a dangerous combination. *Am J Med Med Sci.* (2013) 3:6. 10.5923/j.ajmms.20130304.03 22499009

[77] Mikkelsen K Stojanovska L Polenakovic M Bosevski M Apostolopoulos V. Exercise and mental health. *Maturitas.* (2017) 106:48–56. 10.1016/j.maturitas.2017.09.003 29150166

[78] Fiuza-Luces C Santos-Lozano A Joyner M Carrera-Bastos P Picazo O Zugaza J Exercise benefits in cardiovascular disease: beyond attenuation of traditional risk factors. *Nat Rev Cardiol.* (2018) 15:731–43. 10.1038/s41569-018-0065-1 30115967

