# Corrigendum: Home-based exercise program in the indeterminate form of Chagas disease (PEDI-CHAGAS study): a study protocol for a randomized clinical trial

**DOI:** 10.3389/fmed.2023.1292606

**Published:** 2023-09-27

**Authors:** Mauro F. F. Mediano, Leonardo G. Ribeiro, Rudson S. Silva, Isis G. G. Xavier, Marcelo C. Vieira, Tatiana R. Gonçalves, Vitor B. Paravidino, Juliana P. Borges, Luiz Fernando Rodrigues Junior, Henrique S. Costa, Michel S. Reis, Livia C. Liporagi-Lopes, Pablo Martinez-Amezcua, Paula S. Silva, Gilberto M. Sperandio Da Silva, Andrea S. Sousa, Marcelo T. Holanda, Henrique H. Veloso, Fernanda M. Carneiro, Flavia Mazzoli-Rocha, Andrea R. Costa, Roberto M. Saraiva, Fernanda S. N. S. Mendes, Luiz Henrique C. Sangenis, Alejandro M. Hasslocher-Moreno

**Affiliations:** ^1^Evandro Chagas National Institute of Infectious Diseases, Oswaldo Cruz Foundation, Rio de Janeiro, RJ, Brazil; ^2^Department of Research and Education, National Institute of Cardiology, Rio de Janeiro, RJ, Brazil; ^3^Center for Cardiology and Exercise, Aloysio de Castro State Institute of Cardiology, Rio de Janeiro, RJ, Brazil; ^4^Institute of Collective Health, Federal University of Rio de Janeiro, Rio de Janeiro, RJ, Brazil; ^5^Institute of Social Medicine, State University of Rio de Janeiro, Rio de Janeiro, RJ, Brazil; ^6^Department of Physical Education and Sports, Naval Academy – Brazilian Navy, Rio de Janeiro, RJ, Brazil; ^7^Institute of Physical Education and Sports, State University of Rio de Janeiro, Rio de Janeiro, RJ, Brazil; ^8^Physical Therapy Department, Federal University of Jequitinhonha and Mucuri Valleys, Diamantina, MG, Brazil; ^9^Faculty of Physical Therapy, School of Medicine, Federal University of Rio de Janeiro, Rio de Janeiro, RJ, Brazil; ^10^Faculty of Pharmacy, Federal University of Rio de Janeiro, Rio de Janeiro, RJ, Brazil; ^11^Department of Epidemiology, Johns Hopkins Bloomberg School of Public Health, Baltimore, MD, United States

**Keywords:** physical activity, exercise training, mental health, fitness, chagas disease, quality of life

In the published article, there was an error in the Funding statement. The CAPES funding source (CAPES–Finance Code 001) that will cover the publication fee costs was not included. The correct Funding statement appears below.

This present study was partially funded by grant number E-26/201.436/2021 from Fundação de Amparo à Pesquisa do Estado do Rio de Janeiro—FAPERJ (MM) and partially funded by the Coordenação de Aperfeiçoamento de Pessoal de Nível Superior—Brasil (CAPES)—Finance Code 001.

The authors apologize for this error and state that this does not change the scientific conclusions of the article in any way. The original article has been updated.

