# Supplementary material for: Home-based exercise program in the indeterminate form of Chagas disease (PEDI-CHAGAS study): A study protocol for a randomized clinical trial
Source: Front Med (Lausanne). 2023 Jan 6;9:1087188. doi: 10.3389/fmed.2022.1087188 (PMC9852894; doi:10.3389/fmed.2022.1087188)
Supplement: Supplementary file 1 [file Data_Sheet_1.PDF]

HOME-BASED EXERCISE PROGRAM

Program 1/2

1

Hello,

This booklet is part of a physical exercise program to be done at home. These exercises should be performed three times a week, initially during 30 minutes, until reaching 60 minutes a day. You can choose the best time and place to perform these exercises. Always respect your body limits. Good practice!

LaPClin-Chagas Group

Guidelines and recommendations

1. These exercises will help you to feel better in your daily life
2. Wear light clothes and comfortable shoes (flat and non-slip)
3. Do not perform exercises in the hottest hours
4. While performing the exercises, you may feel a little tired. Keep a pace where you can talk to someone next to you, if necessary
5. Perform your exercises continuously
6. Breathe normally while performing your exercises. Don't hold your breath!
7. Rest for one minute between exercises
8. At the end of the session, perform the stretches. These movements must be done to the limit, where you notice a slight discomfort in the requested muscle, and should be repeated twice for approximately 15 seconds.
9. No exercise should cause pain. If you feel any kind of pain, stop the exercise immediately.
10. Do not start you exercises if you are feeling symptoms such as tiredness, shortness of breath, chest pain or malaise.

11. If you have any question, please contact Dr. Mauro Mediano ([redacted] Prof. Leonardo Ribeiro ([redacted]) or INI Cardiological Center ([redacted]).

Your exercise session has three phases:

1. Warm up

2. Training

3. Cool down

Exercises progression

1st and 2nd weeks: Perform 1 set of each exercise

3rd and 4th weeks: Perform 2 sets of each exercise

5th to 12th weeks: Perform 3 sets of each exercise

13th week: Change to the physical exercise program at booklet 2

1. Warm Up (Perform the movements slowly)

1.1	Standing, arms along the body, lean the torso with the hands touching the thighs	10 repetitions
1.2	Open and close your hands	10 repetitions
1.3	With your hands closed, move your hands back and forth	10 repetitions
1.4	Flex and extend your elbows	10 repetitions
1.5	Raise and lower your hands above your head	10 repetitions
1.6	Raise your knee to waist height and lower, one at a time (with wall support)	10 repetitions

1.1. Standing, arms along the body, lean the torso with the hands touching the thighs

Number of repetitions: 10

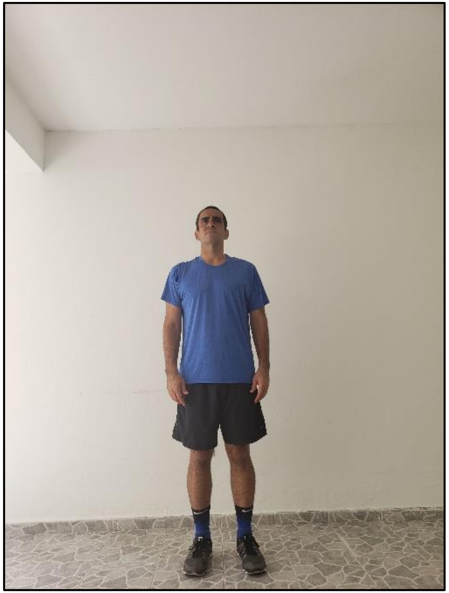

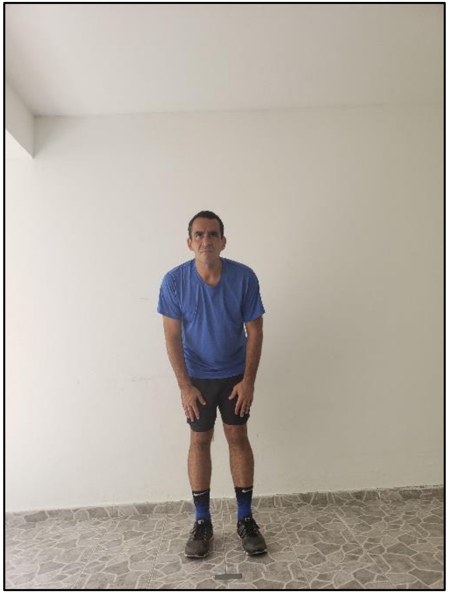

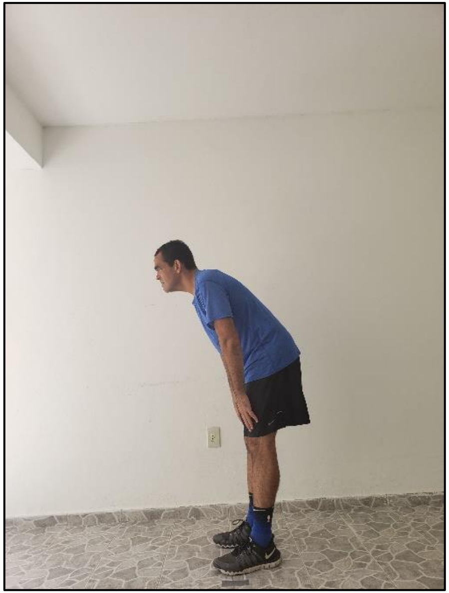

1.2. Open and close your hands

Number of repetitions: 10

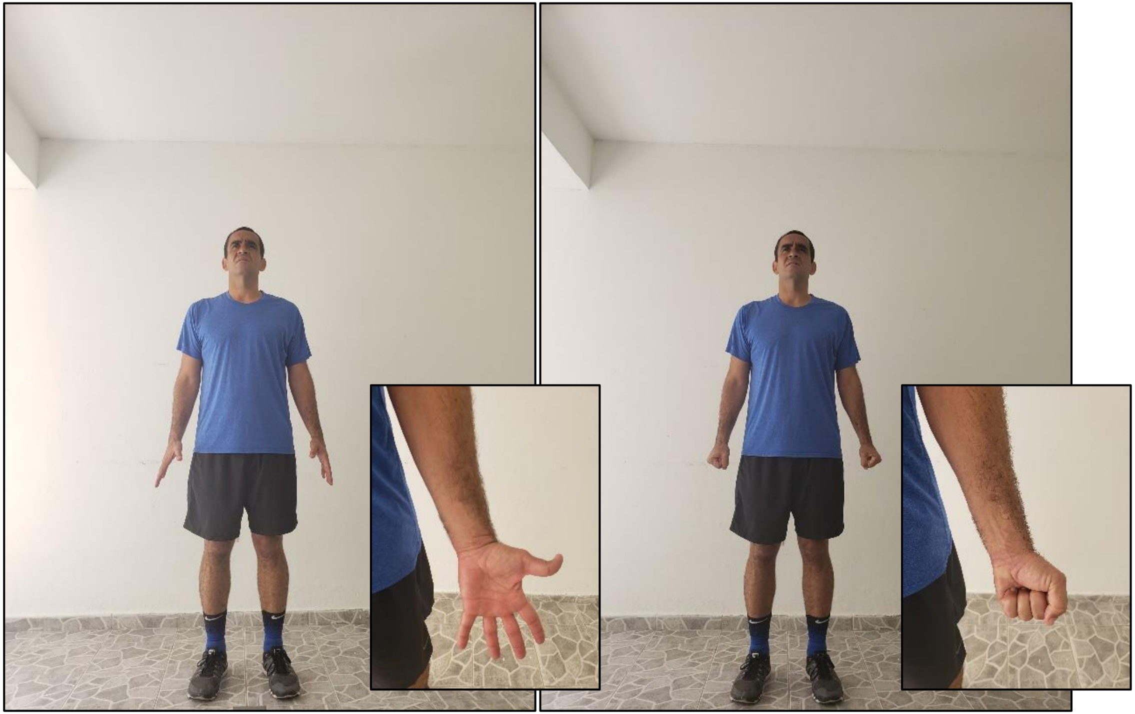

1.3. With your hands closed, move your hands back and forth

Number of repetitions: 10

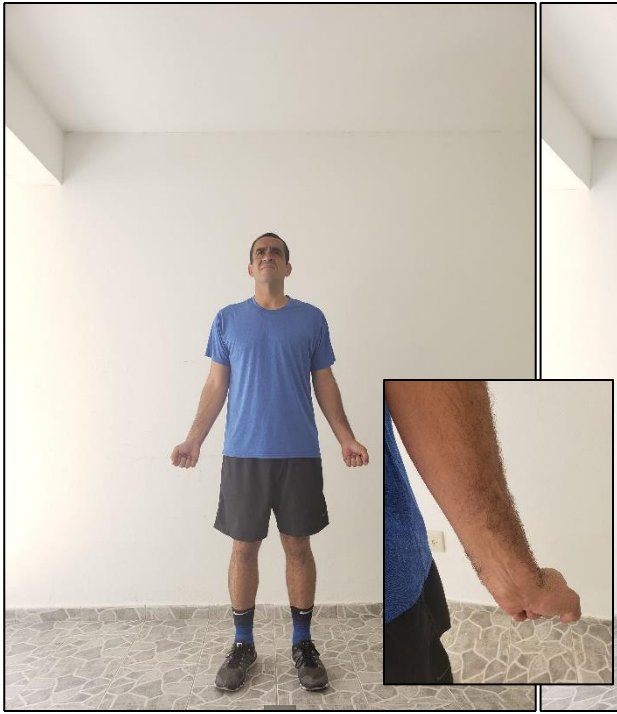

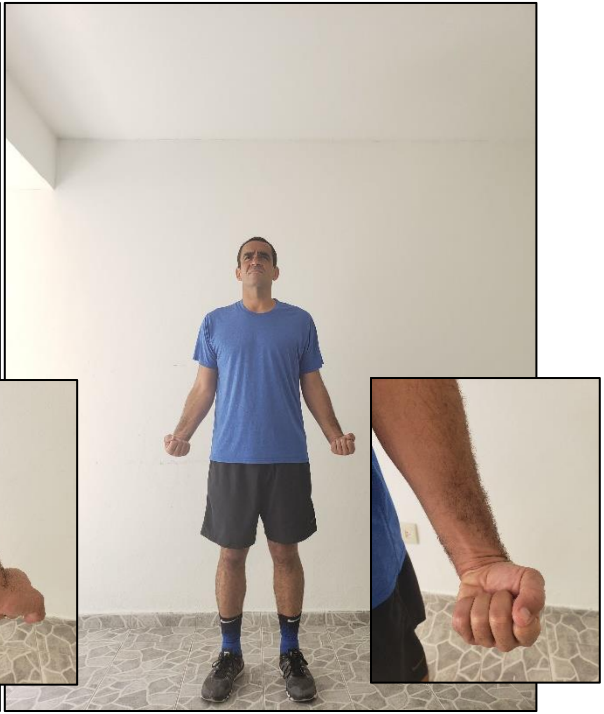

1.4. Flex and extend your elbows

Number of repetitions: 10

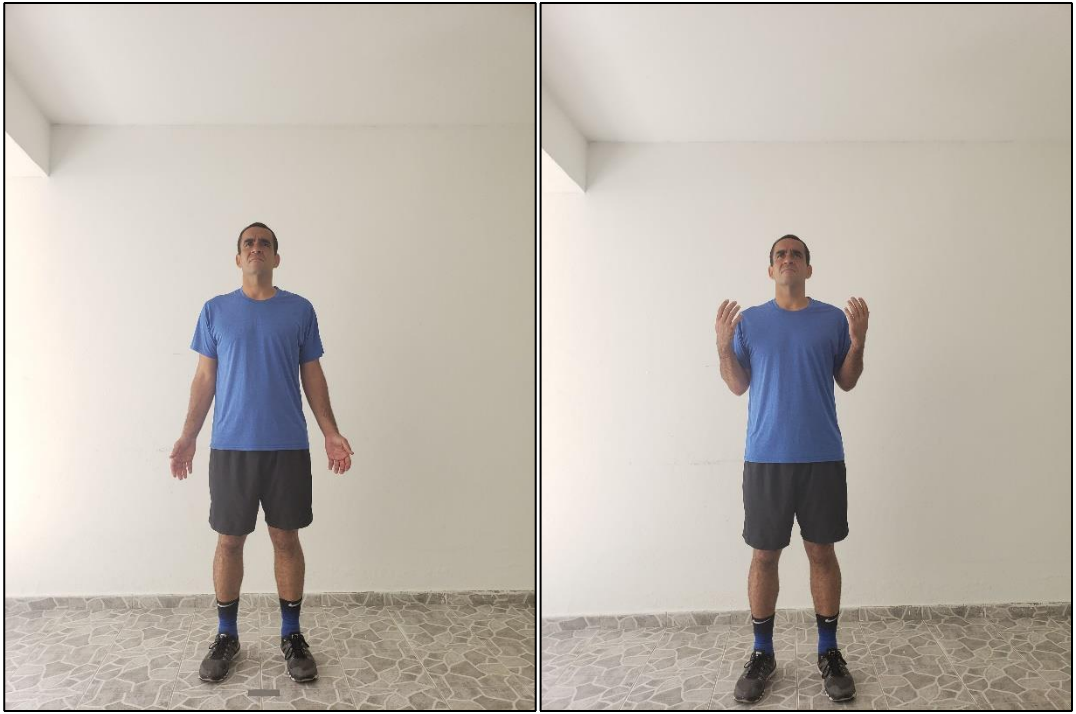

1.5. Raise and lower your hands above your head

Number of repetitions: 10

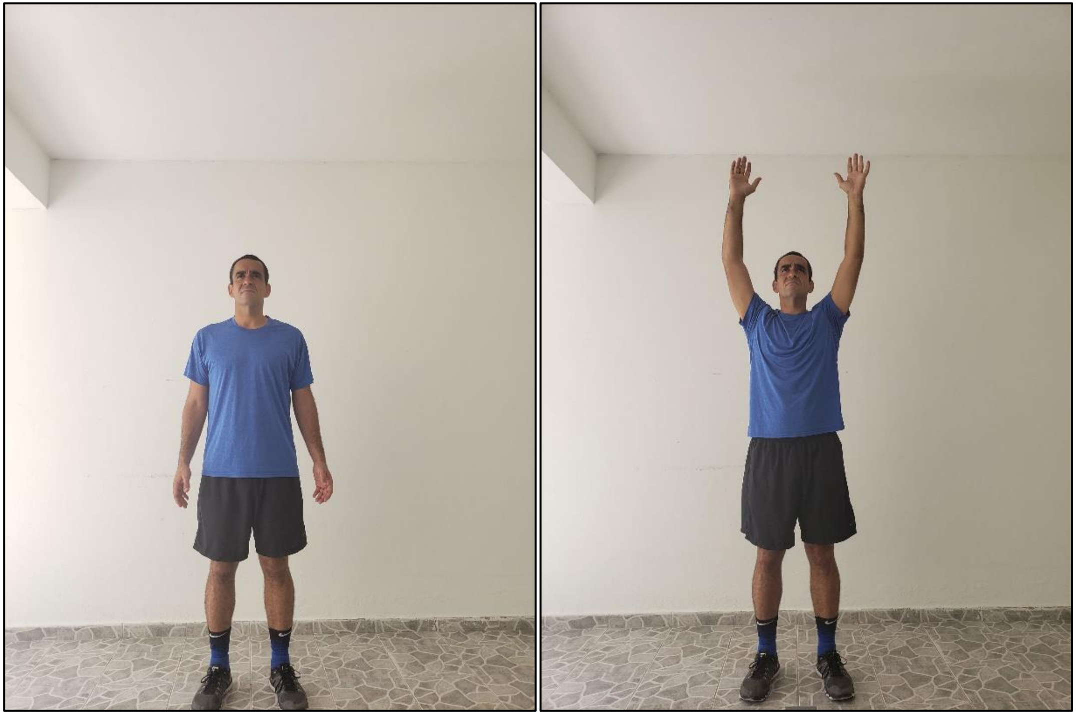

1.6. Raise your knee to waist height and lower, one at a time (with wall support)

Number of repetitions: 10

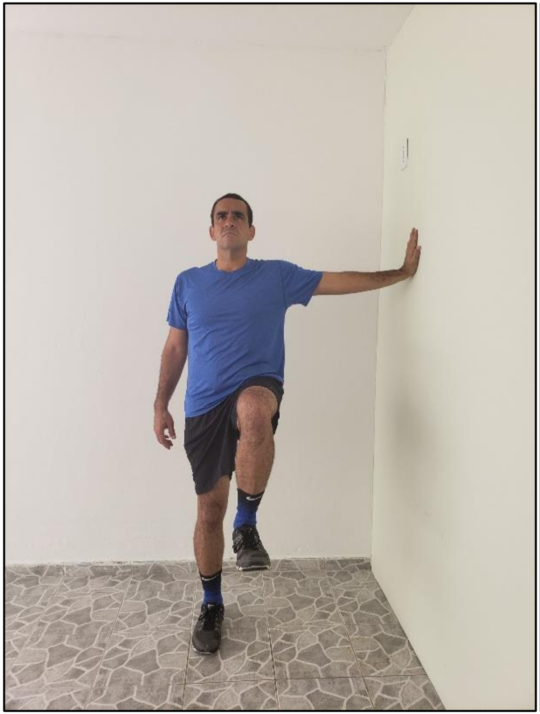

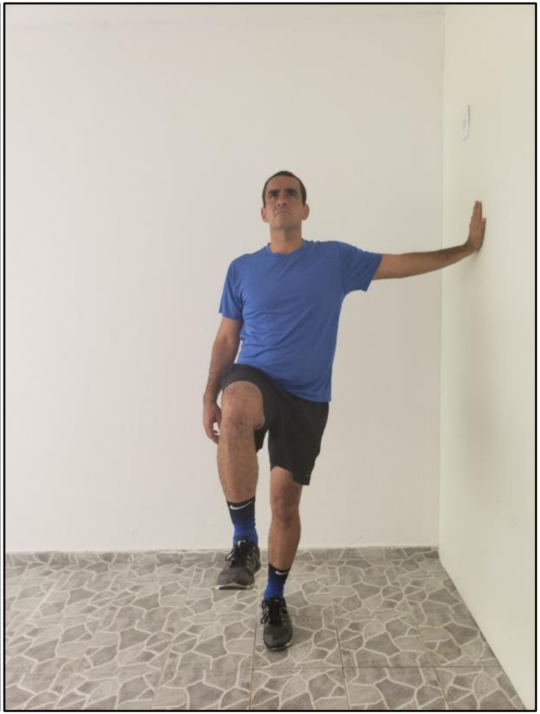

2. Training

2.1	Chair stands - stand-up and sit down on a chair	10 repetitions
2.2	Walk back and forth, flexing and extending your elbows	10 repetitions
2.3	Calf raise - lift and lower your heels (with wall support)	10 repetitions
2.4	Side pass, opening and closing arms	10 repetitions
2.5	Wall push ups	10 repetitions
2.6	Walk in place	2 minutes
2.7	Pulling one arm at a time with support	10 repetitions
2.8	Alternate arm and leg raise	10 repetitions

Important: 1 minute rest between exercise sets

2.1. Chair stands - stand-up and sit down on a chair

Number of repetitions: 10

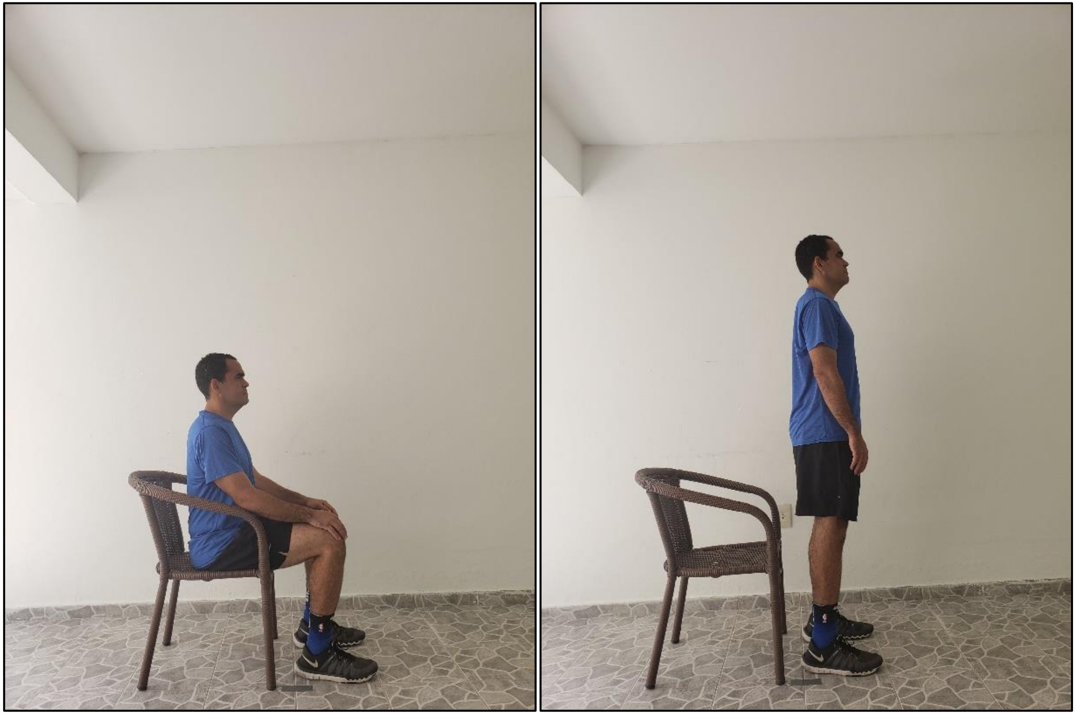

2.2. Walk back and forth, flexing and extending your elbows

Number of repetitions: 10

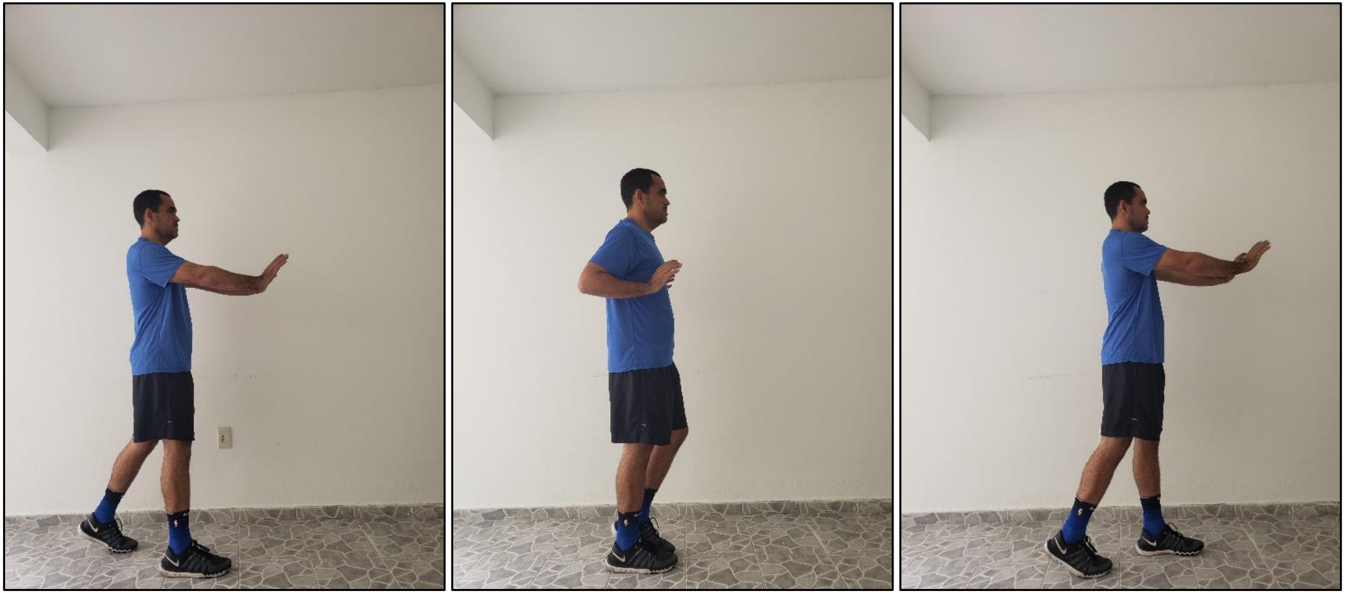

2.3. Calf raise - lift and lower your heels (with wall support)

Number of repetitions: 10

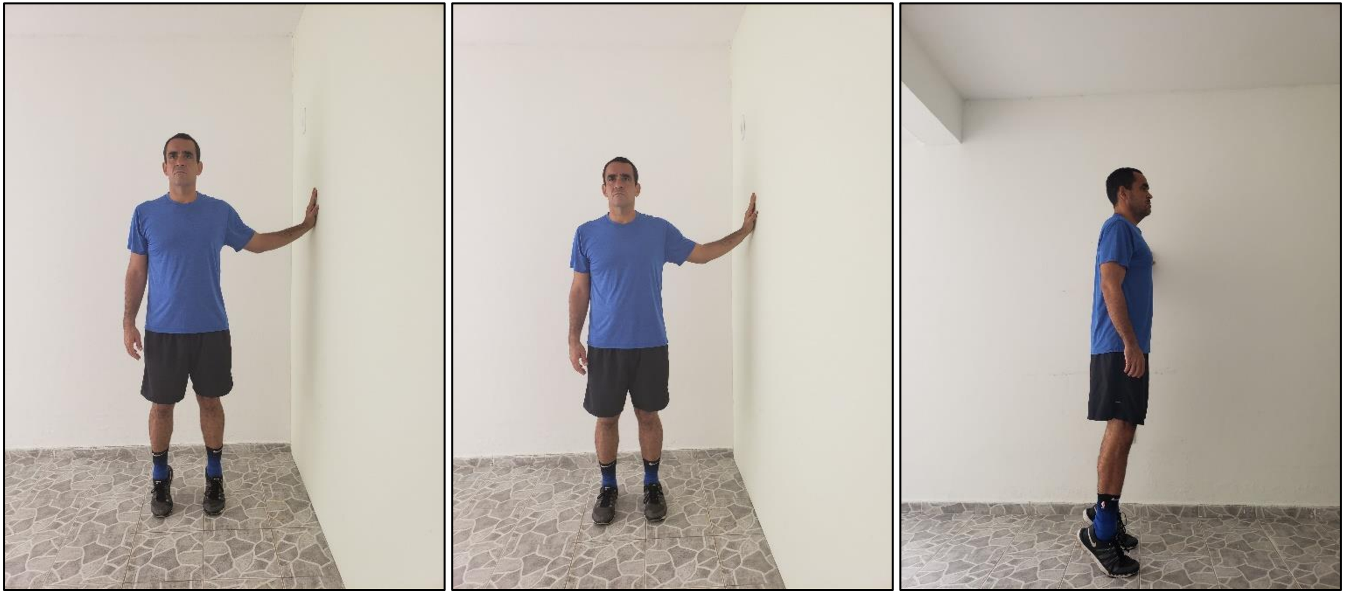

2.4. Side pass, opening and closing the arms

Number of repetitions: 10

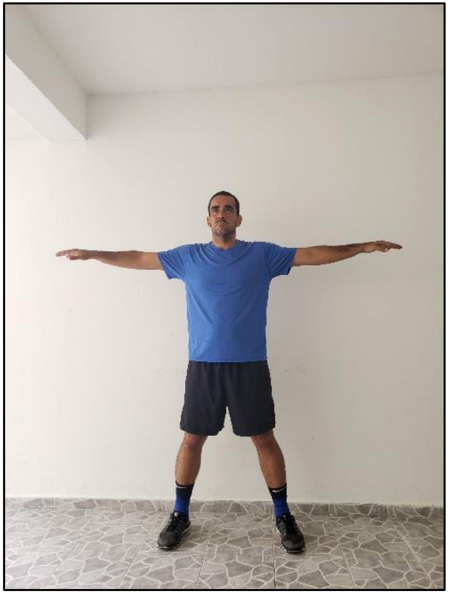
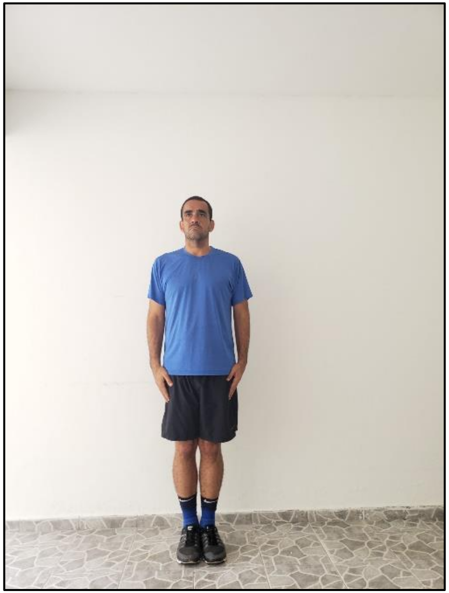
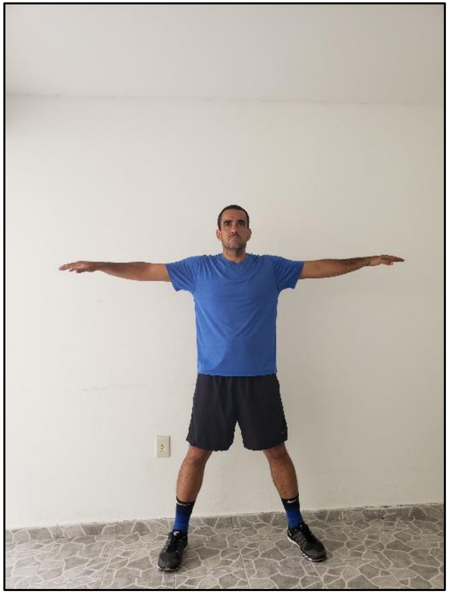

2.5. Wall push ups

Number of repetitions: 10

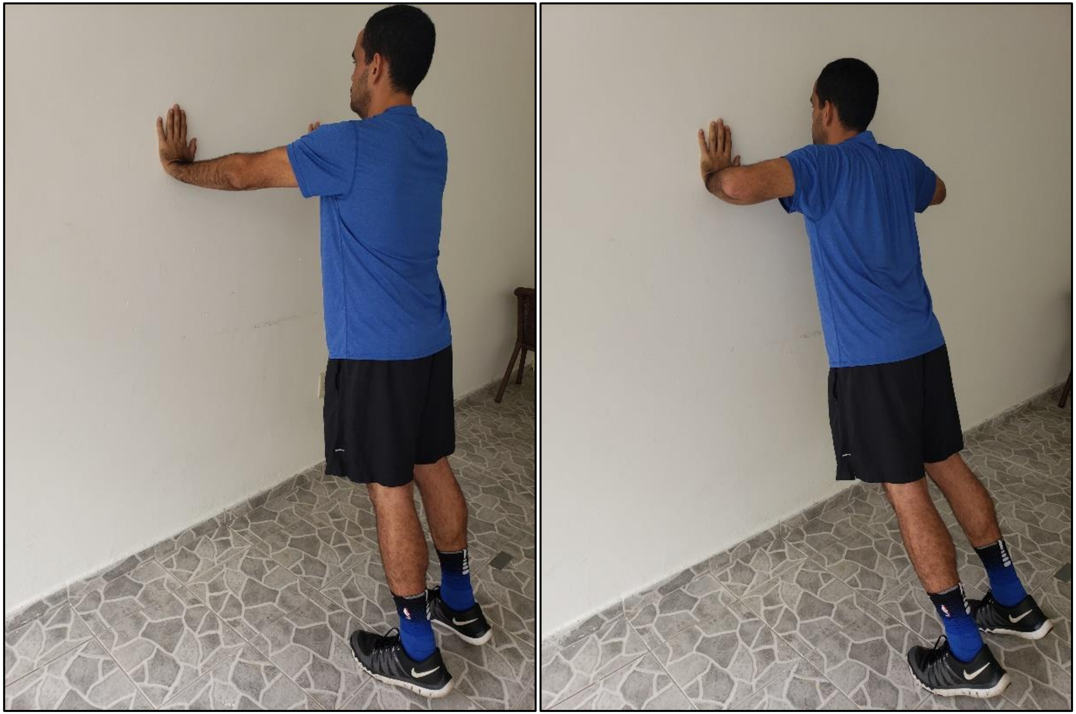

2.6. Walk in place

Time of execution: 2 minutes

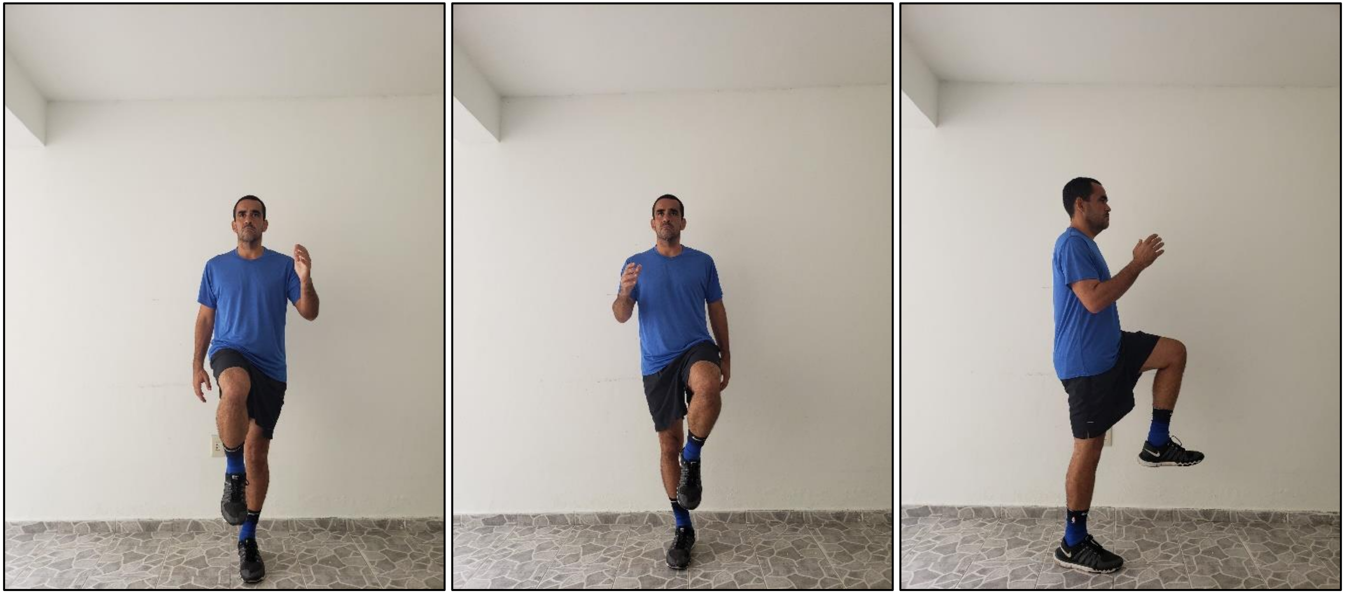

2.7. Pulling one arm at a time with support

Number of repetitions: 10

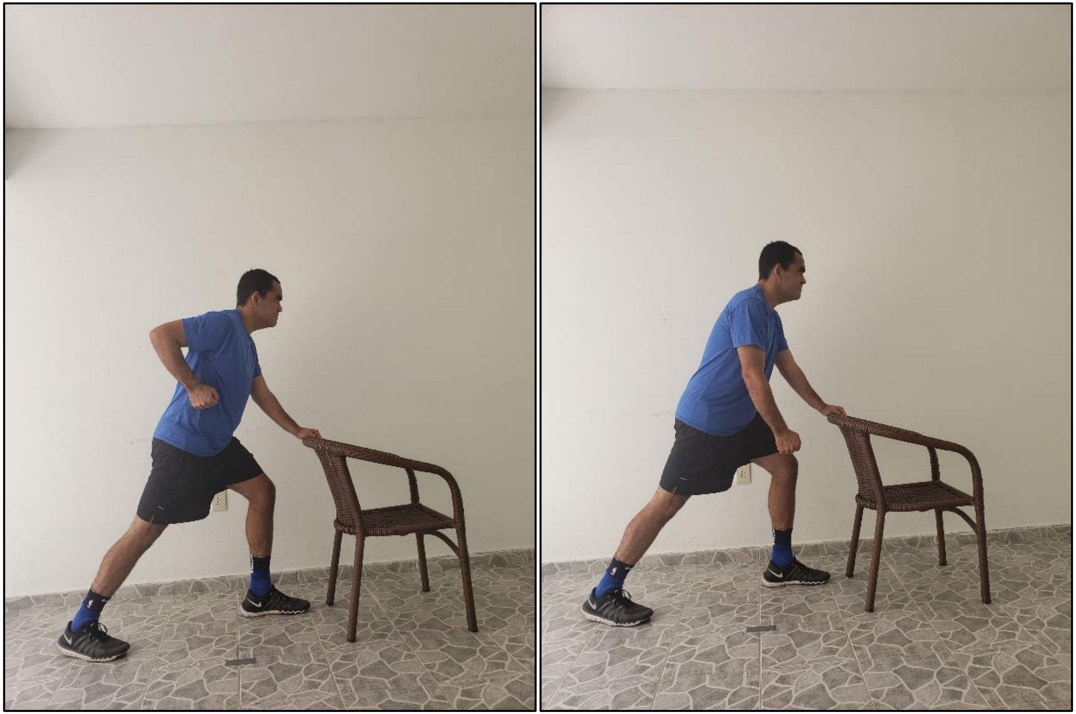

2.8. Alternate arm and leg raise

Number of repetitions: 10

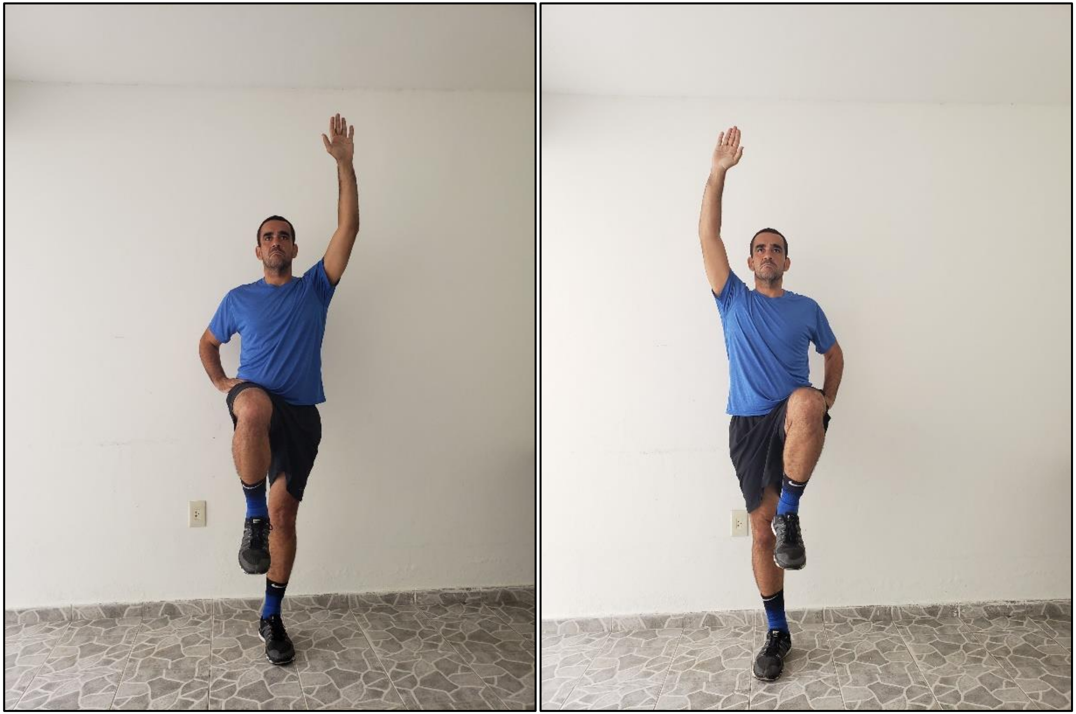

3. Cool down

3.1	Stretching 1	2 x 15 seconds
3.2	Stretching 2	2 x 15 seconds
3.3	Stretching 3	2 x 15 seconds
3.4	Stretching 4	2 x 15 seconds

3.1. Stretching 1

Execution time: 15 seconds for each side (twice)

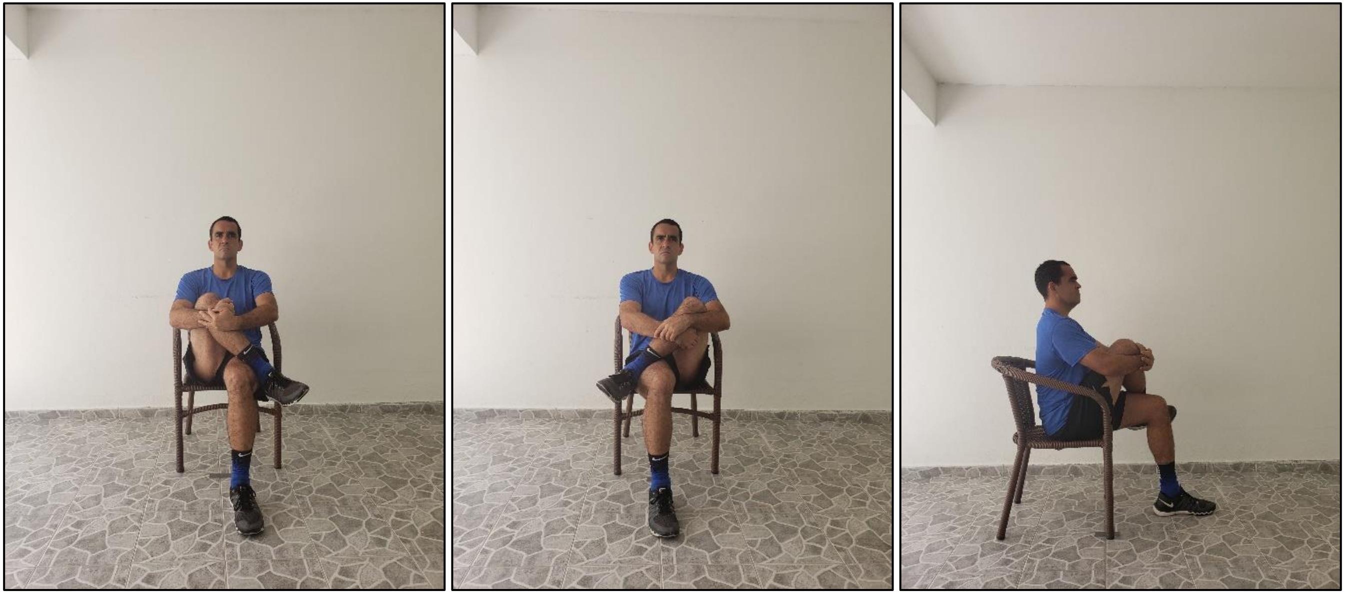

3.2. Stretching 2

Execution time: 15 seconds for each side (twice)

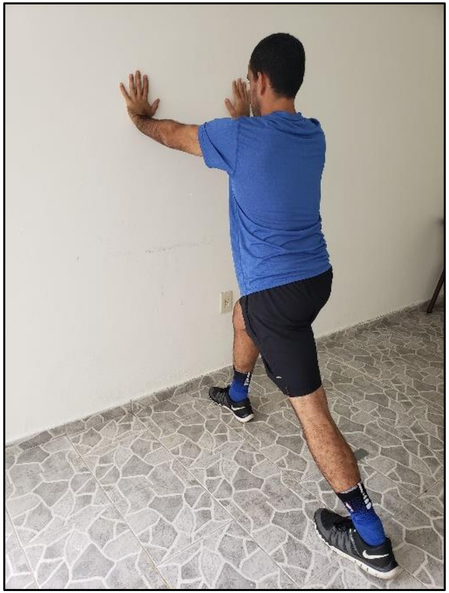
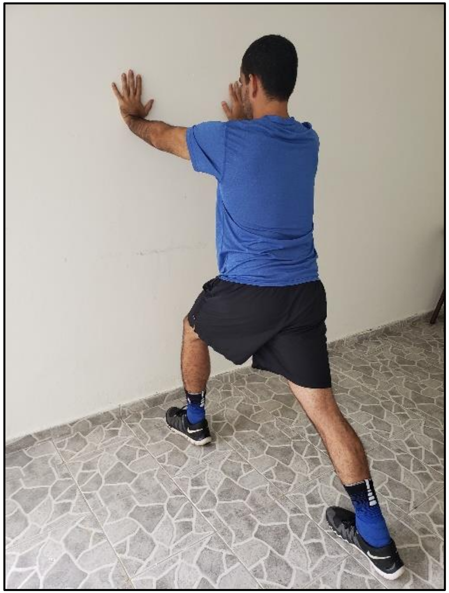
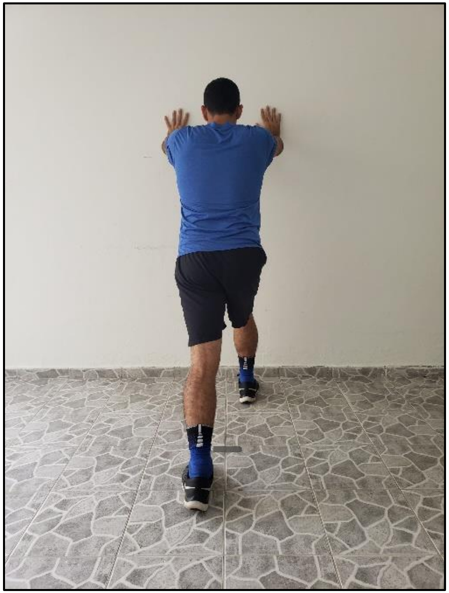

3.3. Stretching 3

Execution time: 15 seconds for each side (twice)

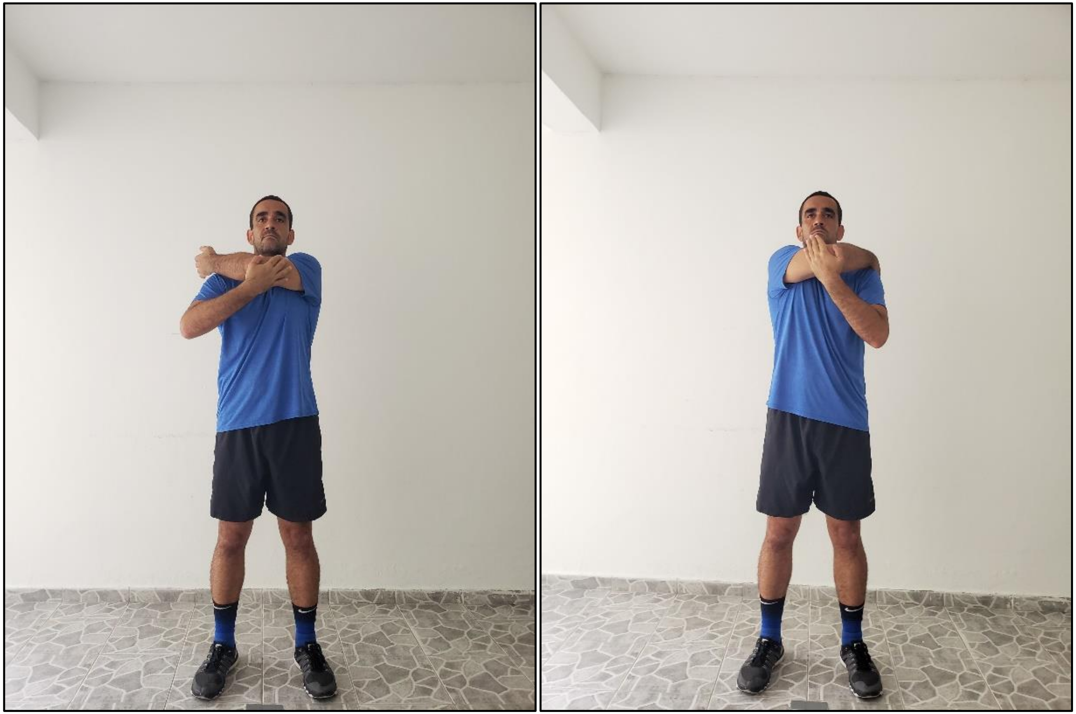

3.4. Stretching 4

Execution time: 15 seconds for each side (twice)

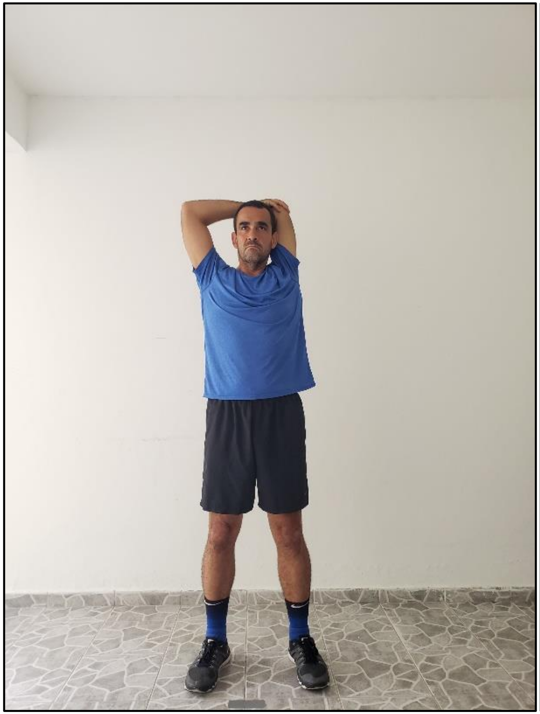
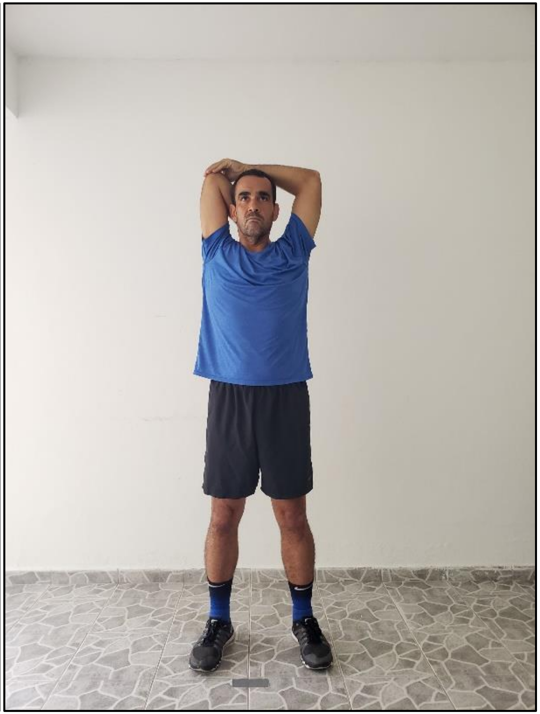

4. Participation record:

Mark with an X the days you performed your exercises.

January					1	2	3	4	5	6	7	8	9	10	11	12	13
14	15	16	17	18	19	20	21	22	23	24	25	26	27	28	29	30	31

February					1	2	3	4	5	6	7	8	9	10	11	12	13
14	15	16	17	18	19	20	21	22	23	24	25	26	27	28			

March					1	2	3	4	5	6	7	8	9	10	11	12	13
14	15	16	17	18	19	20	21	22	23	24	25	26	27	28	29	30	31

April					1	2	3	4	5	6	7	8	9	10	11	12	13
14	15	16	17	18	19	20	21	22	23	24	25	26	27	28	29	30	

May					1	2	3	4	5	6	7	8	9	10	11	12	13

14	15	16	17	18	19	20	21	22	23	24	25	26	27	28	29	30	31

June					1	2	3	4	5	6	7	8	9	10	11	12	13
14	15	16	17	18	19	20	21	22	23	24	25	26	27	28	29	30	

July					1	2	3	4	5	6	7	8	9	10	11	12	13
14	15	16	17	18	19	20	21	22	23	24	25	26	27	28	29	30	31

August					1	2	3	4	5	6	7	8	9	10	11	12	13
14	15	16	17	18	19	20	21	22	23	24	25	26	27	28	29	30	31

September					1	2	3	4	5	6	7	8	9	10	11	12	13
14	15	16	17	18	19	20	21	22	23	24	25	26	27	28	29	30	

October					1	2	3	4	5	6	7	8	9	10	11	12	13
14	15	16	17	18	19	20	21	22	23	24	25	26	27	28	29	30	31

November					1	2	3	4	5	6	7	8	9	10	11	12	13
14	15	16	17	18	19	20	21	22	23	24	25	26	27	28	29	30	

December					1	2	3	4	5	6	7	8	9	10	11	12	13
14	15	16	17	18	19	20	21	22	23	24	25	26	27	28	29	30	31
