# Supplementary material for: Home-based exercise program in the indeterminate form of Chagas disease (PEDI-CHAGAS study): A study protocol for a randomized clinical trial
Source: Front Med (Lausanne). 2023 Jan 6;9:1087188. doi: 10.3389/fmed.2022.1087188 (PMC9852894; doi:10.3389/fmed.2022.1087188)
Supplement: Supplementary file 2 [file Data_Sheet_2.PDF]

FIOCRUZ
FUNDAÇÃO OSWALDO CRUZ

INI
Instituto Nacional de Infectologia
Evandro Chagas

HOME-BASED EXERCISE PROGRAM

Program 2/2

2

Hello,

As in the first exercise program, this training should be performed three times a week, for 60 minutes a day.

We would like to thank you once again for your participation in this study.

LaPClin-Chagas Group

Guidelines and recommendations

1. These exercises will help you feel better in your everyday life
2. Wear light clothes and comfortable shoes (flat and non-slip)
3. Do not perform exercises in the hottest hours
4. While performing the exercises, you may feel a little tired. Keep a pace where you can talk to someone next to you, if necessary
5. Perform your exercises continuously
6. Breathe normally while performing your exercises. Don't hold your breath!
7. Rest for one minute between exercises
8. At the end of the session, perform the stretches. These movements must be done to the limit, where you notice a slight discomfort in the requested muscle, and should be repeated twice for approximately 15 seconds
9. No exercise should cause pain. If you feel any kind of pain, stop the exercise immediately
10. Do not start you exercises if you are feeling symptoms such as tiredness, shortness of breath, chest pain or malaise

Your exercise session has three phases:

1. Warm up

2. Training

3. Cool down

Exercises progression

13th and 14th weeks: Perform each exercise twice

15th to 24th weeks: Perform each exercise 3 times

1. Warm Up (Perform the movements slowly)

1.1	Standing, arms along the body, lean the torso with the hands touching the thighs	10 repetitions
1.2	Open and close your hands	10 repetitions
1.3	With your hands closed, move your hands back and forth	10 repetitions
1.4	Flex and extend your elbows	10 repetitions
1.5	Raise and lower your hands above your head	10 repetitions
1.6	Raise your knee to waist height and lower, one at a time (with wall support)	10 repetitions

1.1. Standing, arms along the body, lean the torso with the hands touching the thighs

Number of repetitions: 10

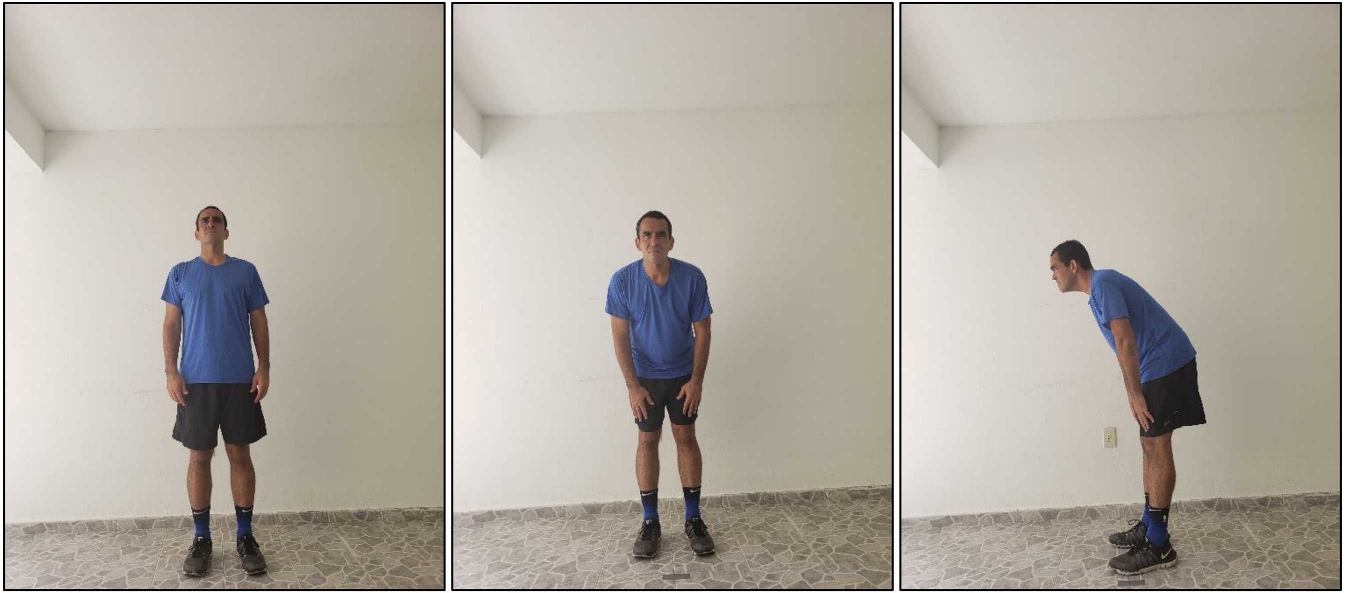

1.2. Open and close your hands

Number of repetitions: 10

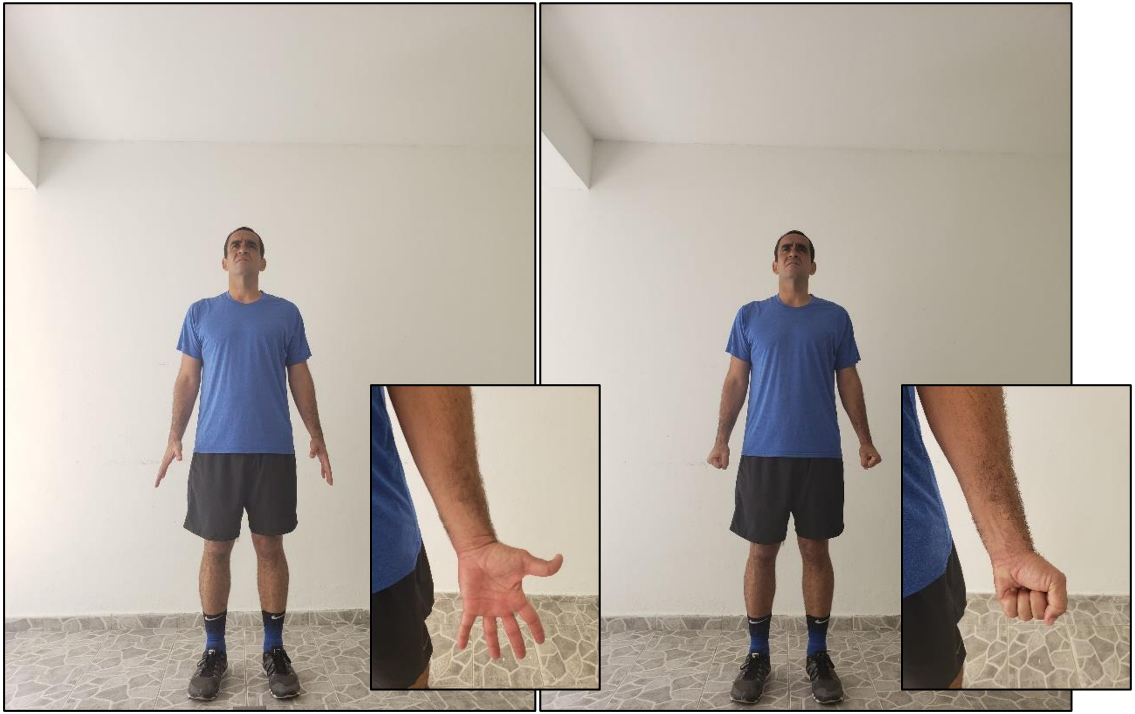

1.3. With your hands closed, move your hands back and forth

Number of repetitions: 10

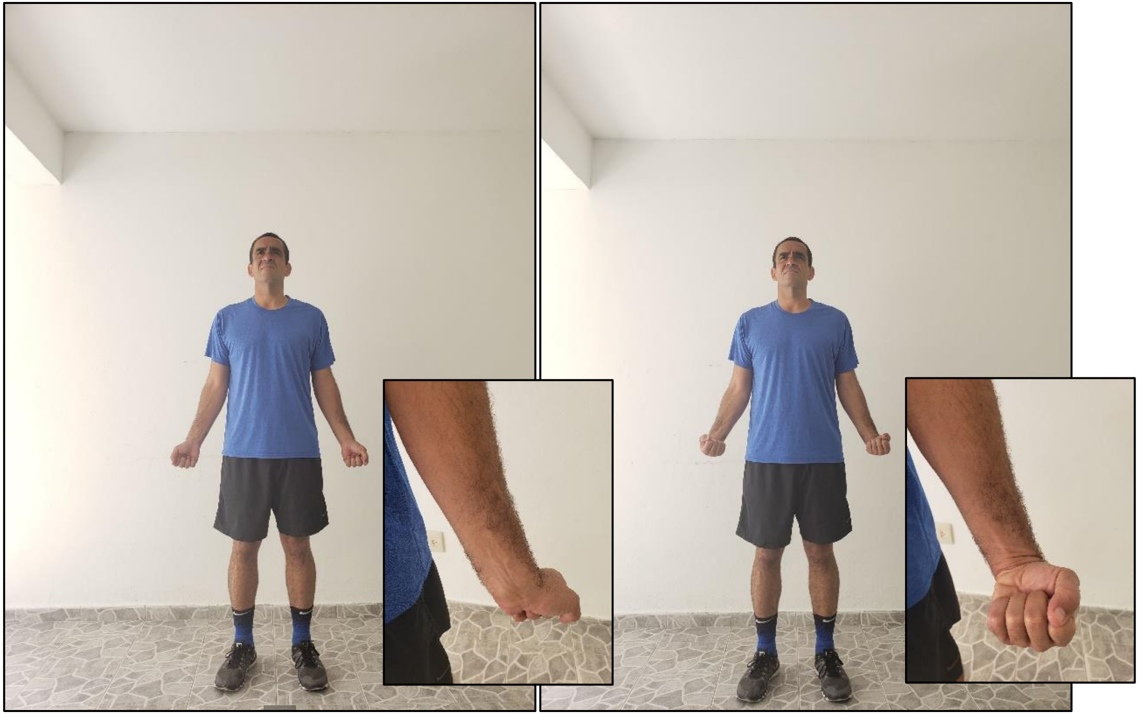

1.4. Flex and extend your elbows

Number of repetitions: 10

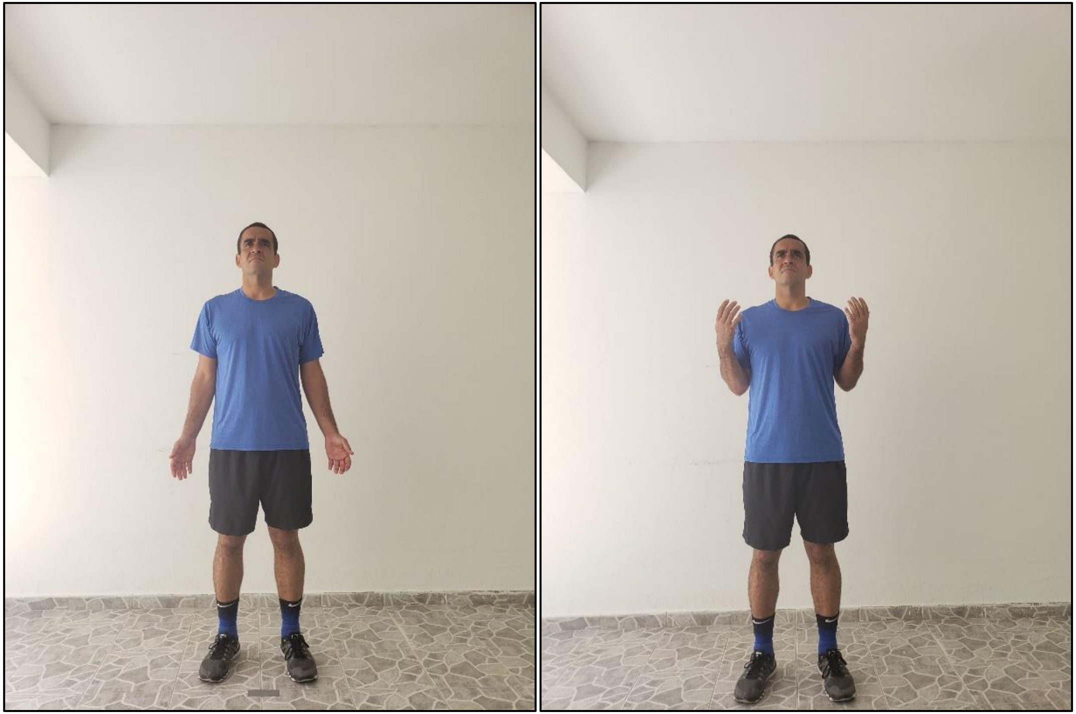

1.5. Raise and lower your hands above your head

Number of repetitions: 10

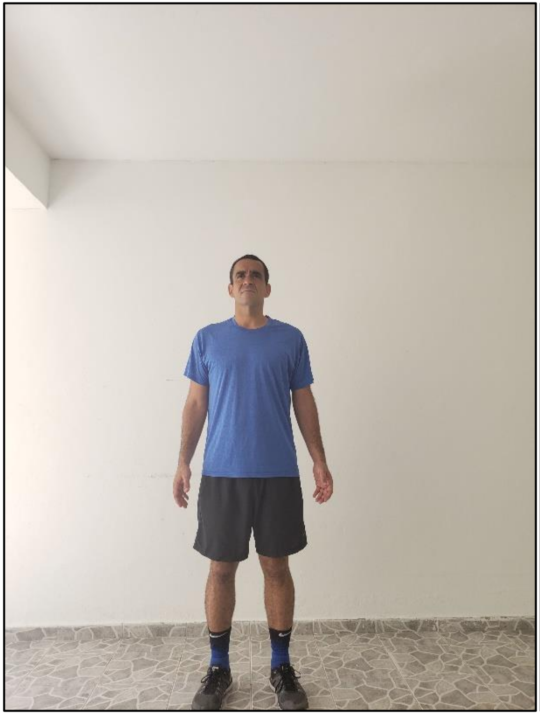
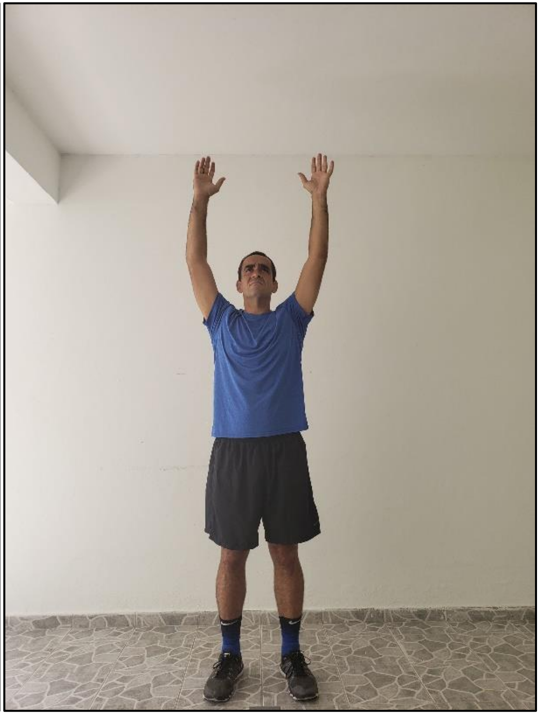

1.6. Raise your knee to waist height and lower, one at a time (with wall support)

Number of repetitions: 10

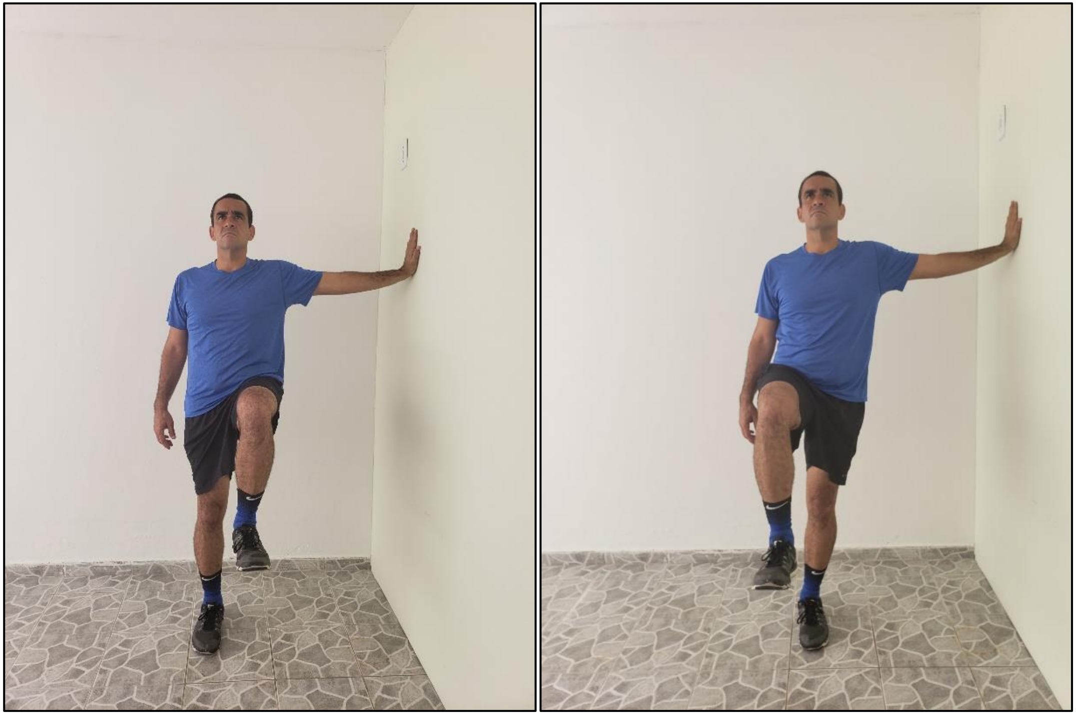

2. Training

2.1	Walk in place	2 minutes
2.2	Holding the ball, flex your elbow and extend above your head	10 repetitions
2.3	Throw the ball up and catch it	10 repetitions
2.4	Chair stands holding the ball – stand up and sit down on a chair	10 repetitions
2.5	Trunk rotation holding the ball and knee lift	10 repetitions
2.6	Walk back and forth bouncing the ball	10 repetitions
2.7	Flex your knee, one at a time (with wall support)	10 repetitions
2.8	Adapted jumping jack (touch your head and lift your heels)	10 repetitions

Important: 1 minute rest between exercise sets

2.1. Walk in place

Execution time: 2 minutes

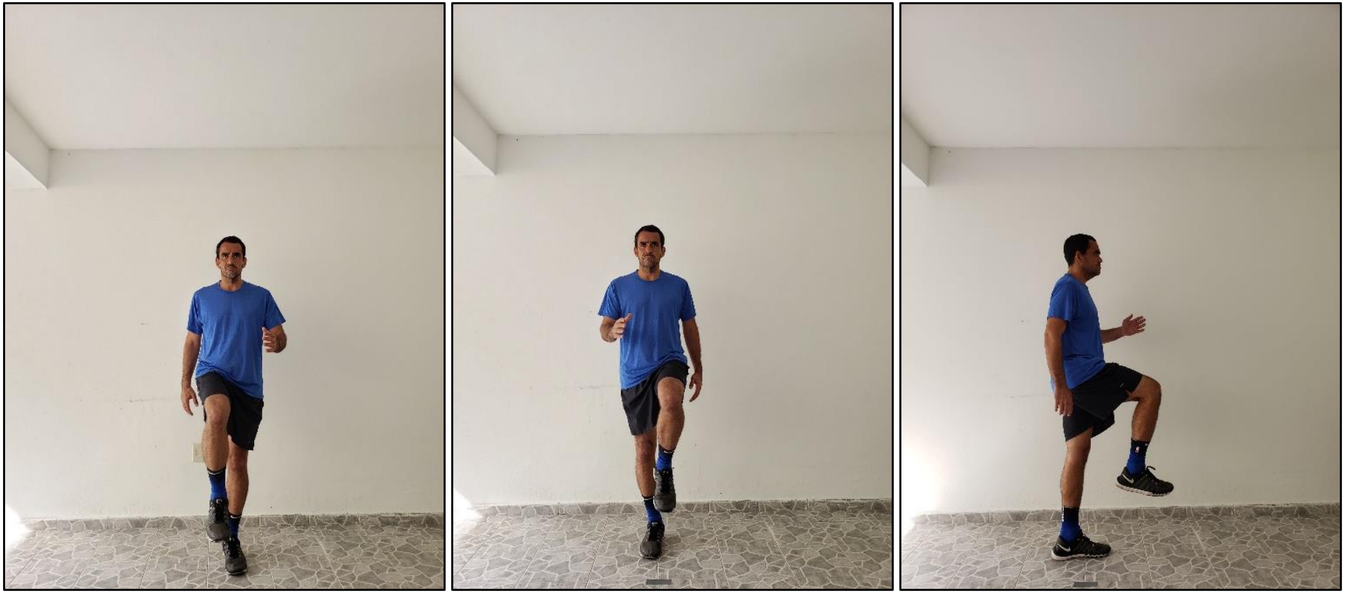

2.2. Holding the ball, flex your elbow and extend above your head

Number of repetitions: 10

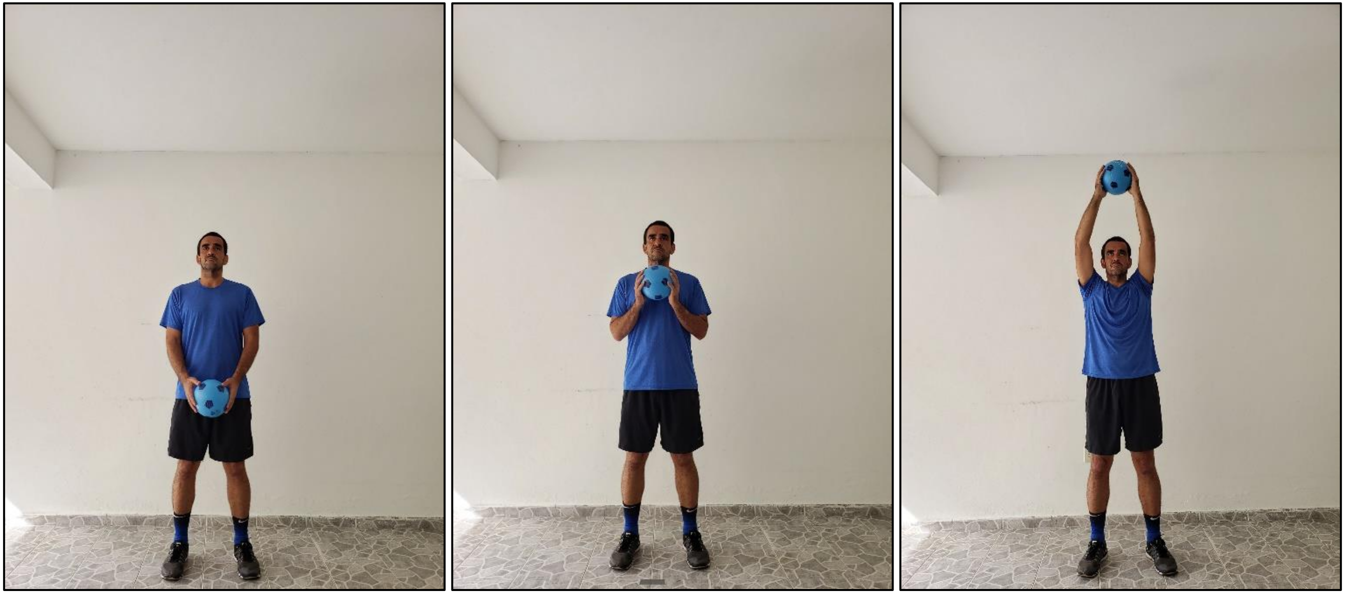

2.3. Throw the ball up and catch it

Number of repetitions: 10

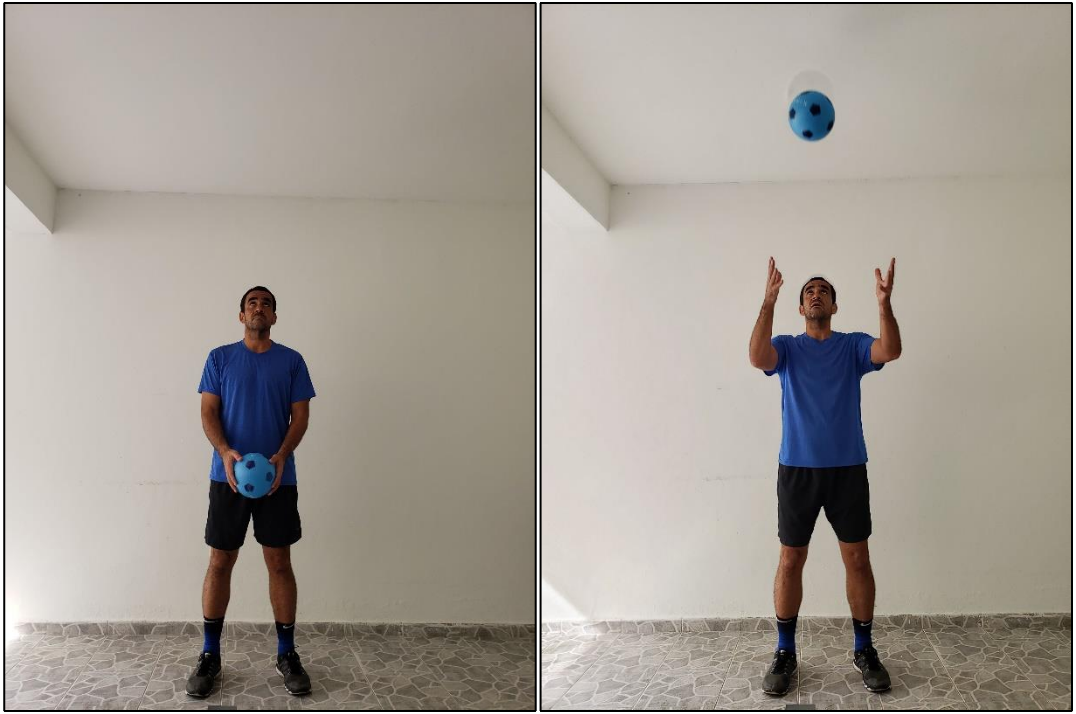

2.4. Chair stands holding the ball – stand up and sit down on a chair

Number of repetitions: 10

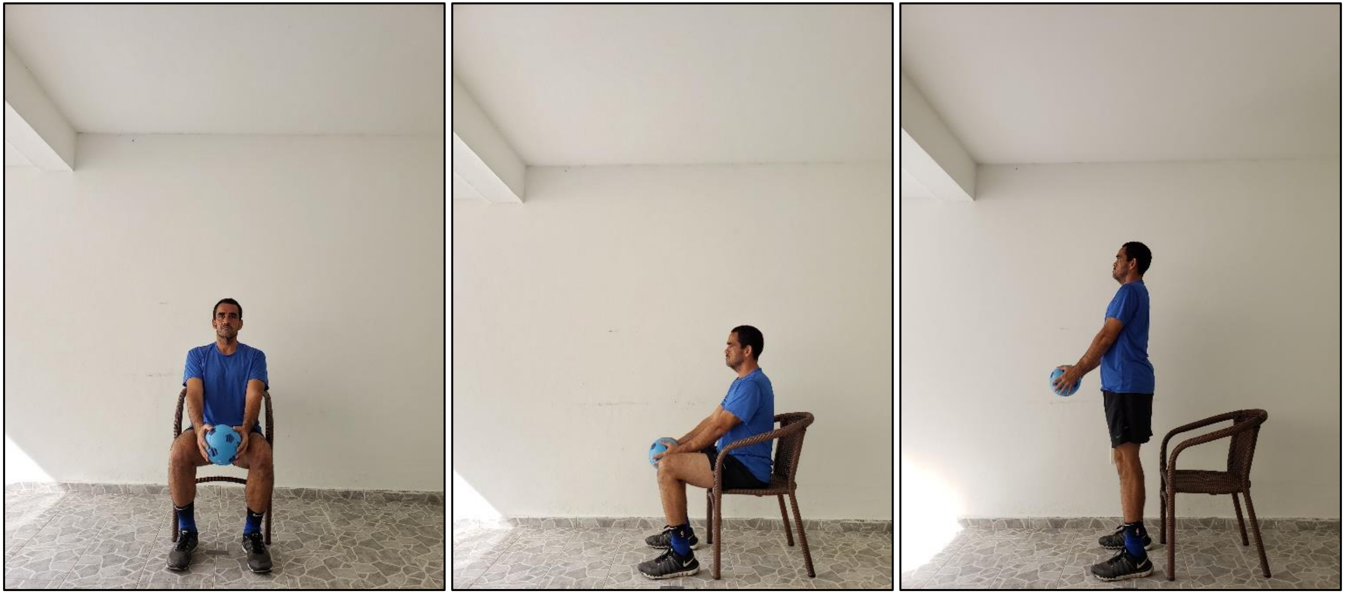

2.5. Trunk rotation holding the ball and knee lift

Number of repetitions: 10

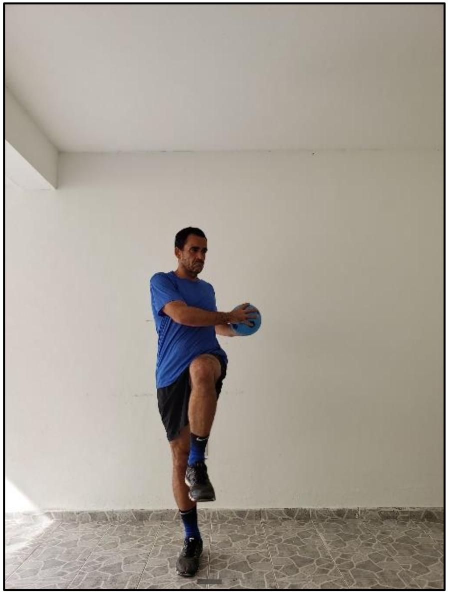
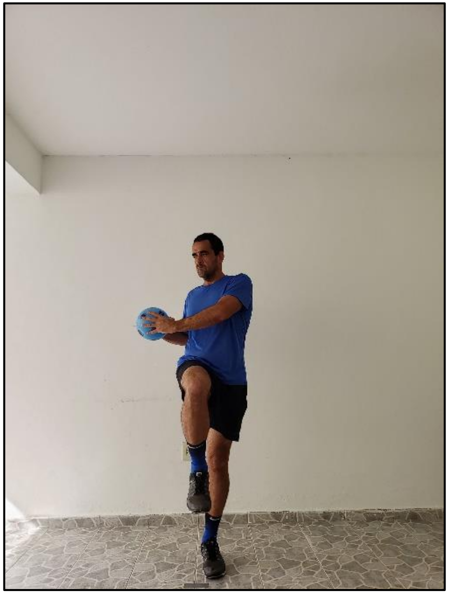
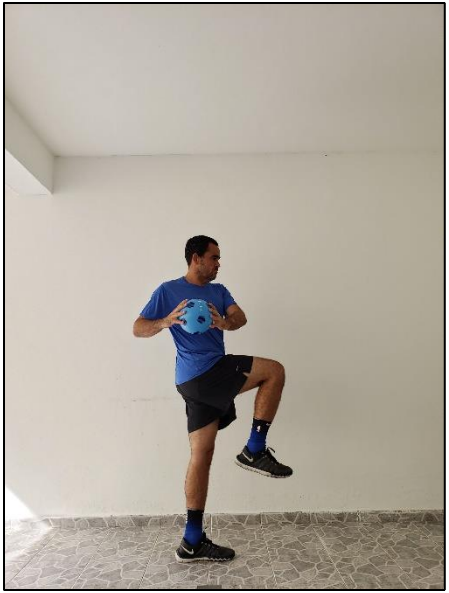

2.6. Walk back and forth bouncing the ball

Number of repetitions: 10

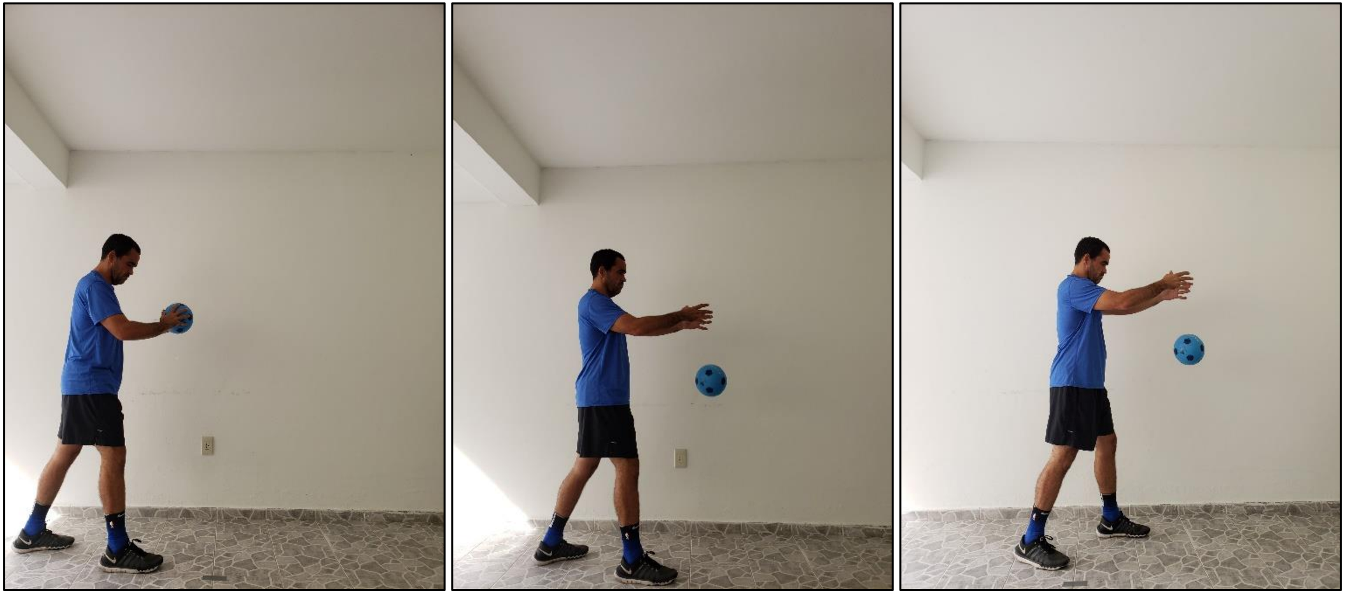

2.7. Flex your knee, one at a time (with wall support)

Number of repetitions: 10

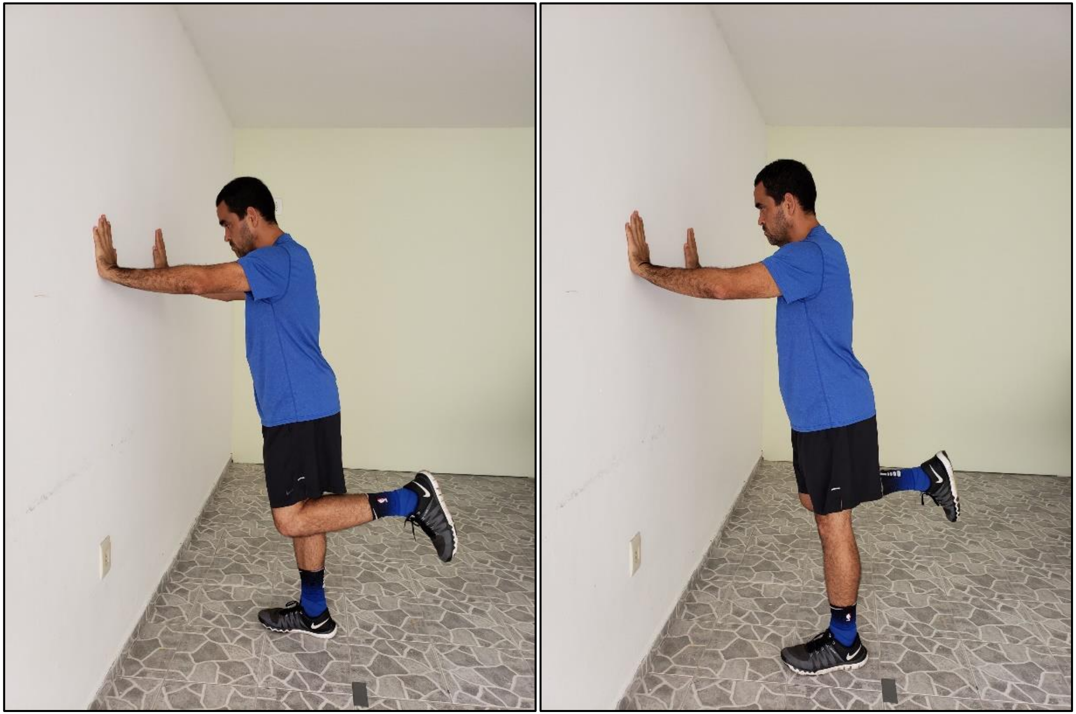

2.8. Adapted jumping jack (touch your head and lift your heels)

Number of repetitions: 10

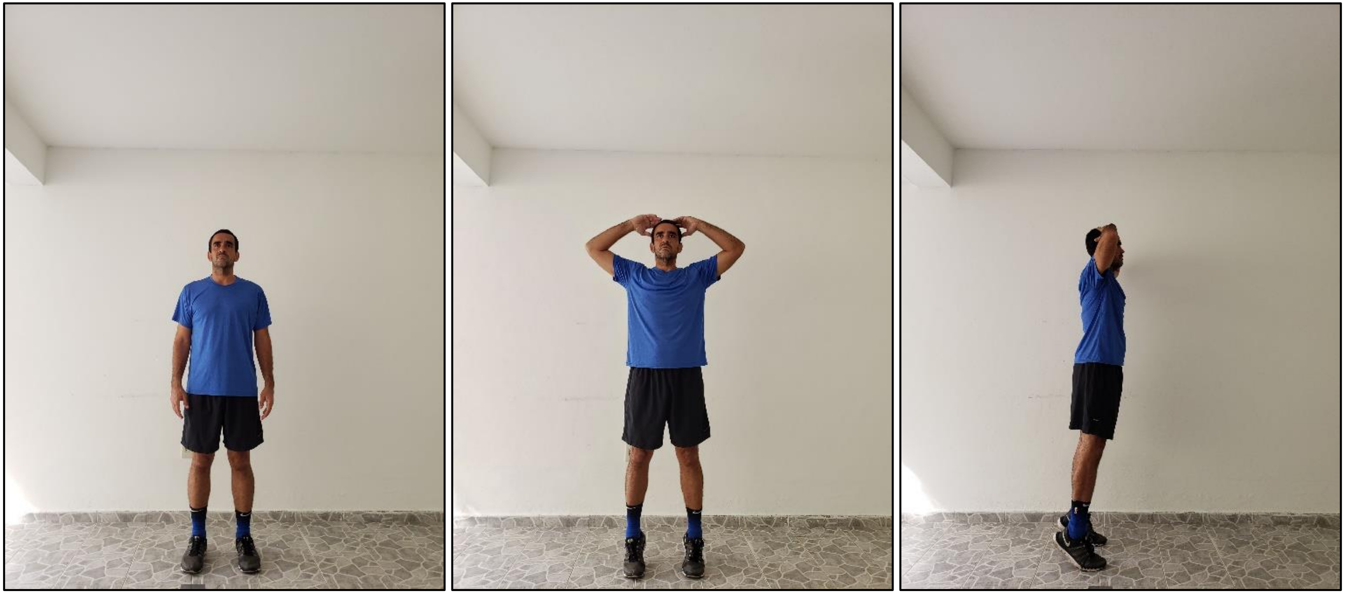

3. Cool down

3.1	Stretching 1	2 X 15 seconds
3.2	Stretching 2	2 X 15 seconds
3.3	Stretching 3	2 X 15 seconds
3.4	Stretching 4	2 X 15 seconds

3.1. Stretching 1

Execution time: 15 seconds for each side (twice)

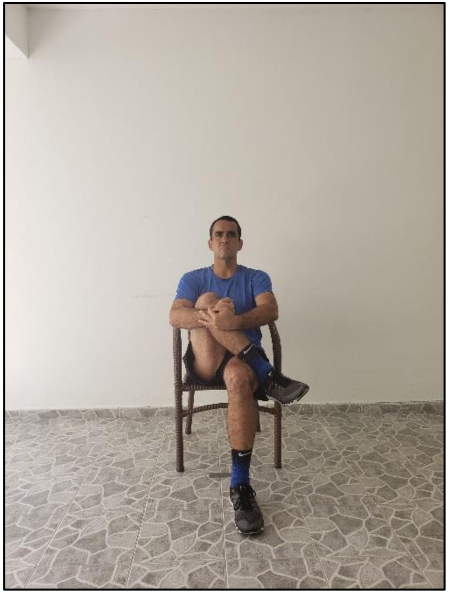
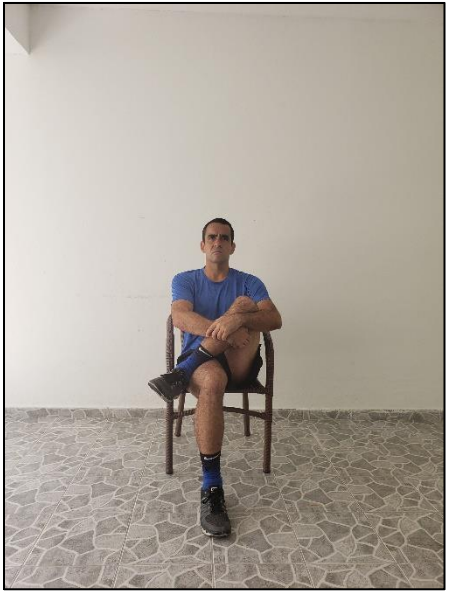
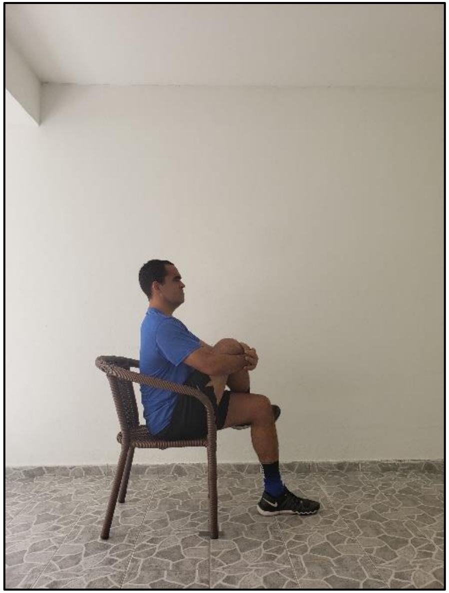

3.2. Stretching 2

Execution time: 15 seconds for each side (twice)

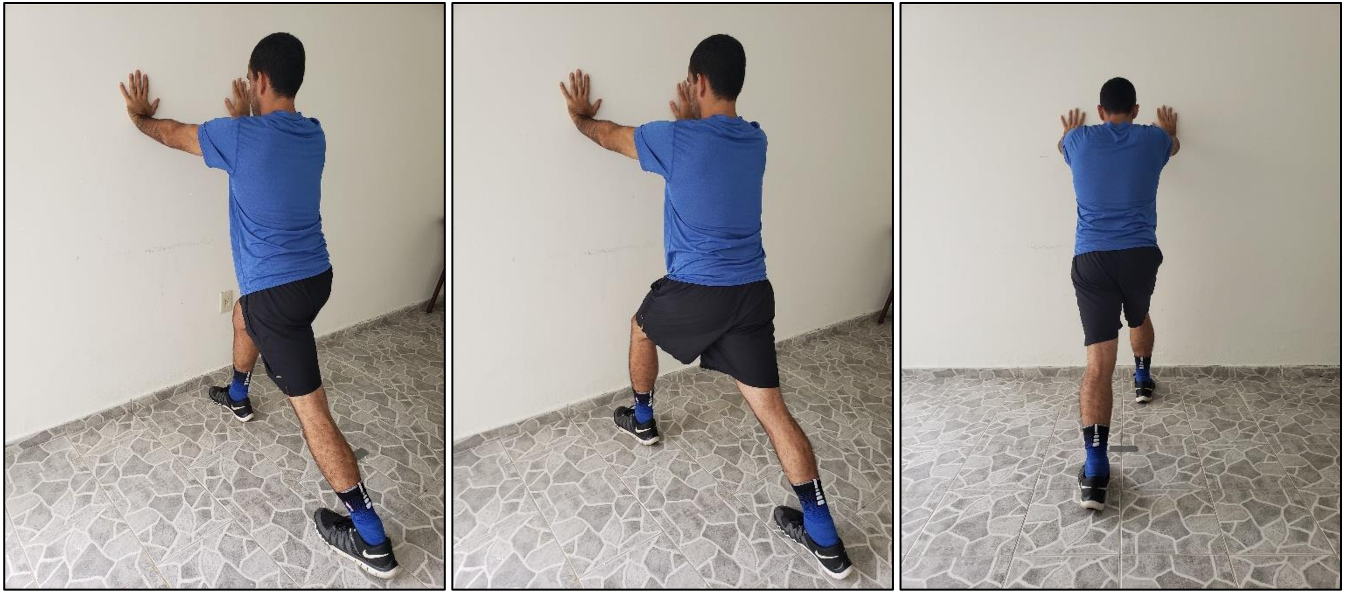

3.3. Stretching 3

Execution time: 15 seconds for each side (twice)

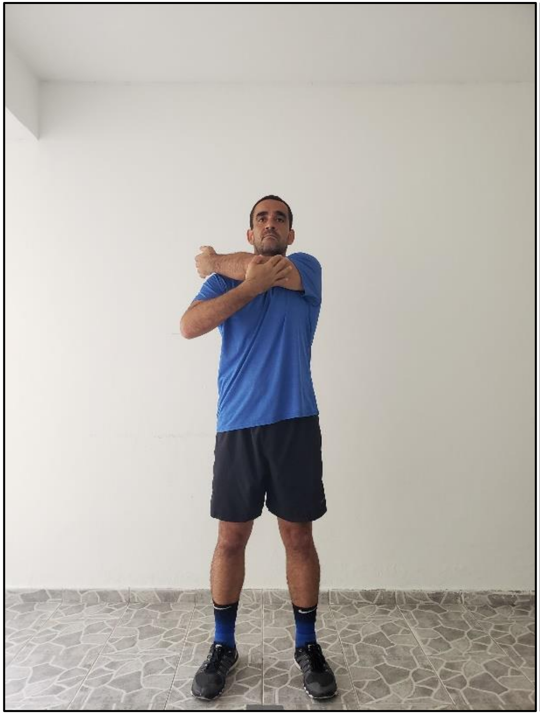

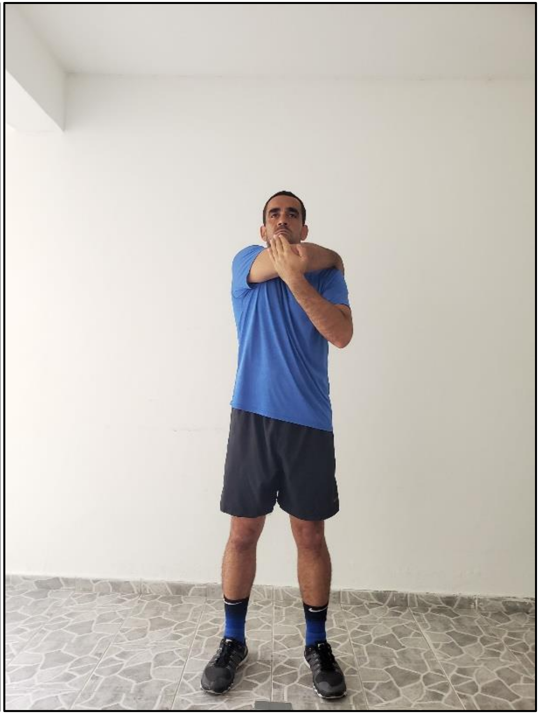

3.4. Stretching 4

Execution time: 15 seconds for each side (twice)

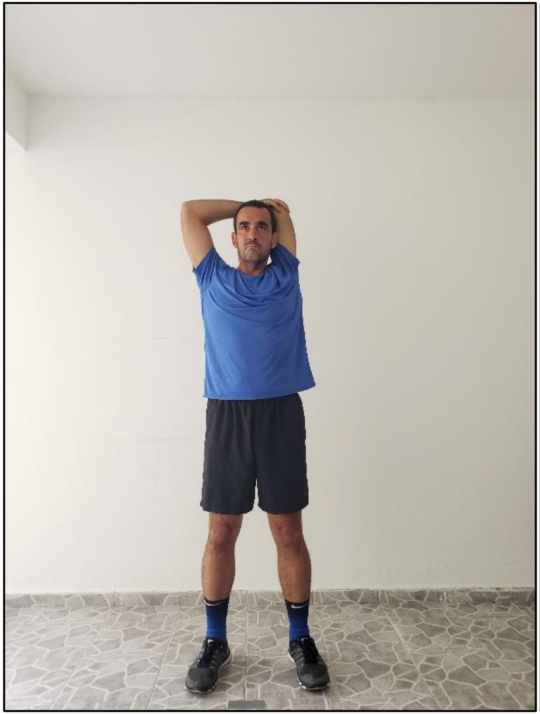

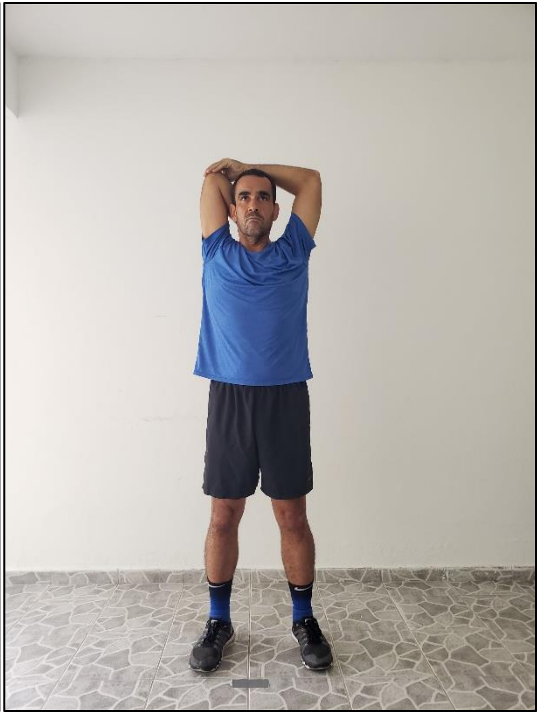

4. Participation record:

Mark with an X the days you performed your exercises

January					1	2	3	4	5	6	7	8	9	10	11	12	13
14	15	16	17	18	19	20	21	22	23	24	25	26	27	28	29	30	31

February					1	2	3	4	5	6	7	8	9	10	11	12	13
14	15	16	17	18	19	20	21	22	23	24	25	26	27	28			

March					1	2	3	4	5	6	7	8	9	10	11	12	13
14	15	16	17	18	19	20	21	22	23	24	25	26	27	28	29	30	31

April					1	2	3	4	5	6	7	8	9	10	11	12	13
14	15	16	17	18	19	20	21	22	23	24	25	26	27	28	29	30	

May					1	2	3	4	5	6	7	8	9	10	11	12	13

14	15	16	17	18	19	20	21	22	23	24	25	26	27	28	29	30	31

June					1	2	3	4	5	6	7	8	9	10	11	12	13
14	15	16	17	18	19	20	21	22	23	24	25	26	27	28	29	30	

July					1	2	3	4	5	6	7	8	9	10	11	12	13
14	15	16	17	18	19	20	21	22	23	24	25	26	27	28	29	30	31

August					1	2	3	4	5	6	7	8	9	10	11	12	13
14	15	16	17	18	19	20	21	22	23	24	25	26	27	28	29	30	31

September					1	2	3	4	5	6	7	8	9	10	11	12	13
14	15	16	17	18	19	20	21	22	23	24	25	26	27	28	29	30	

October					1	2	3	4	5	6	7	8	9	10	11	12	13
14	15	16	17	18	19	20	21	22	23	24	25	26	27	28	29	30	31

November					1	2	3	4	5	6	7	8	9	10	11	12	13
14	15	16	17	18	19	20	21	22	23	24	25	26	27	28	29	30	

December					1	2	3	4	5	6	7	8	9	10	11	12	13
14	15	16	17	18	19	20	21	22	23	24	25	26	27	28	29	30	31
