# Supplementary material for: Home-based exercise program in the indeterminate form of Chagas disease (PEDI-CHAGAS study): A study protocol for a randomized clinical trial
Source: Front Med (Lausanne). 2023 Jan 6;9:1087188. doi: 10.3389/fmed.2022.1087188 (PMC9852894; doi:10.3389/fmed.2022.1087188)
Supplement: Supplementary file 3 [file Data_Sheet_3.PDF]

FIOCRUZ
FUNDAÇÃO OSWALDO CRUZ

INI
Instituto Nacional de Infectologia
Evandro Chagas

PROGRAMA DE EXERCÍCIOS DOMICILIAR

Programa 1/2

1

Olá,

Esta cartilha faz parte de um programa de exercícios físicos para você realizar em sua casa. Os exercícios devem ser realizados três vezes por semana, com duração inicial de 30 minutos até chegarmos a 60 minutos por dia. Você pode escolher o melhor horário e local para realizar os exercícios. Respeite sempre o limite do seu corpo. Bom exercício!

Muito obrigado pela sua participação,

Equipe Lapclin-Chagas

Orientações e recomendações

1. Esses exercícios ajudarão você a se sentir melhor no seu dia a dia.
2. Utilize roupas leves e calçado confortável, sem salto e que não escorregue.
3. Não realize os exercícios em horários muito quentes.
4. Durante a realização dos exercícios, você poderá ficar um pouco cansado(a). Mantenha um esforço no qual seja possível conversar com alguém ao seu lado, caso seja necessário.
5. Execute os exercícios com movimentos contínuos e equilibrados.
6. Respire normalmente durante a execução dos exercícios. Não prenda a respiração!
7. Descanse por um minuto entre os exercícios.
8. Ao término dos exercícios, realize os alongamentos, que devem ser repetidos 2 vezes por cerca de 15 segundos, até o limite onde você perceba um leve desconforto no local do alongamento.
9. Nenhum exercício pode causar dor. Caso isso ocorra, pare o exercício imediatamente.
10. A sessão de exercícios não deverá ser iniciada em caso de sintomas como cansaço, falta de ar, dor no peito ou mal-estar.
11. Em caso de dúvidas entre em contato com o Dr. Mauro Mediano (98521-0609), Prof. Leonardo Ribeiro (97254-0977) ou Centro Cardiológico do INI (3865-9648).

A sua sessão de exercícios tem três etapas a serem realizadas:

1. Aquecimento

2. Parte principal

3. Volta à calma

Progressão dos exercícios

1ª e 2ª semanas: Realizar cada exercício 1 vez

3ª e 4ª semanas: Realizar cada exercício 2 vezes

5ª a 12ª semanas: Realizar cada exercício 3 vezes

13ª semana: Troca do programa de exercícios (2/2)

1. Aquecimento (Executar os movimentos lentamente)

1.1	De pé, braços ao longo do corpo, incline o tronco com as mãos tocando as coxas	10 repetições
1.2	Abra e feche as mãos	10 repetições
1.3	Mãos fechadas, dobre e estique os punhos	10 repetições
1.4	Dobre e estique os cotovelos	10 repetições
1.5	Eleve e abaixe as mãos acima da cabeça	10 repetições
1.6	Eleve um joelho de cada vez até a altura da cintura e volte (apoio na parede)	10 repetições

1.1. De pé, braços ao longo do corpo, incline o tronco com as mãos tocando as coxas

Quantidade: 10 repetições

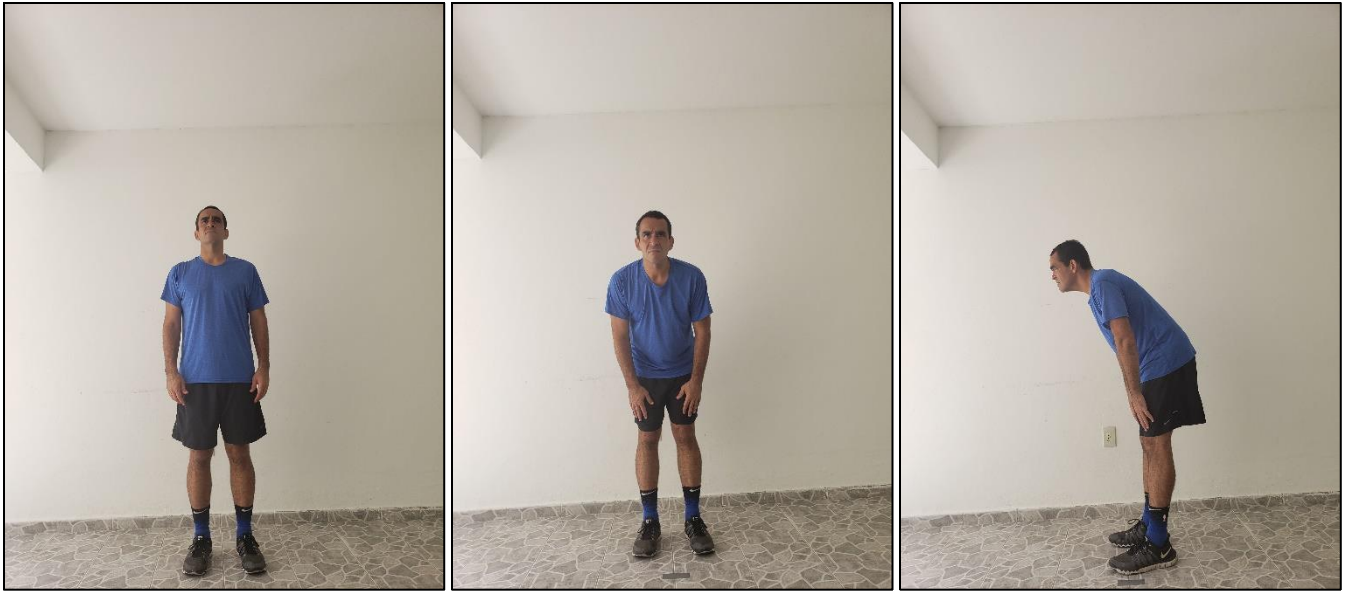

1.2. Abra e feche as mãos

Quantidade: 10 repetições

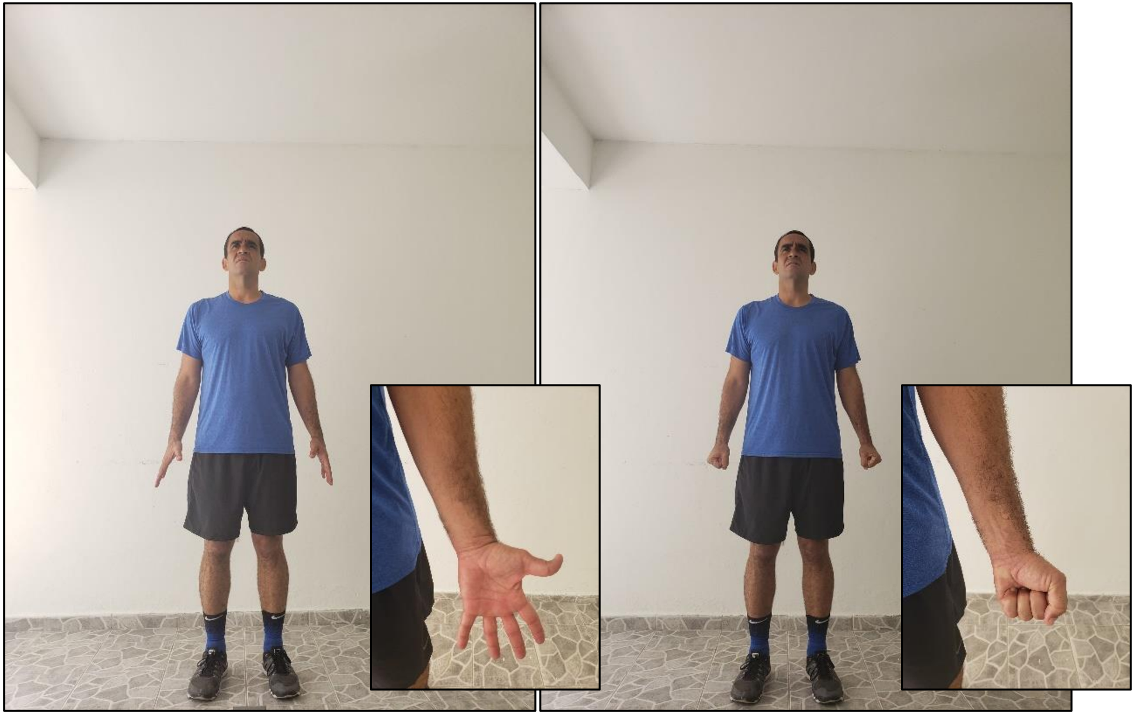

1.3. Mãos fechadas, dobre e estique os punhos

Quantidade: 10 repetições

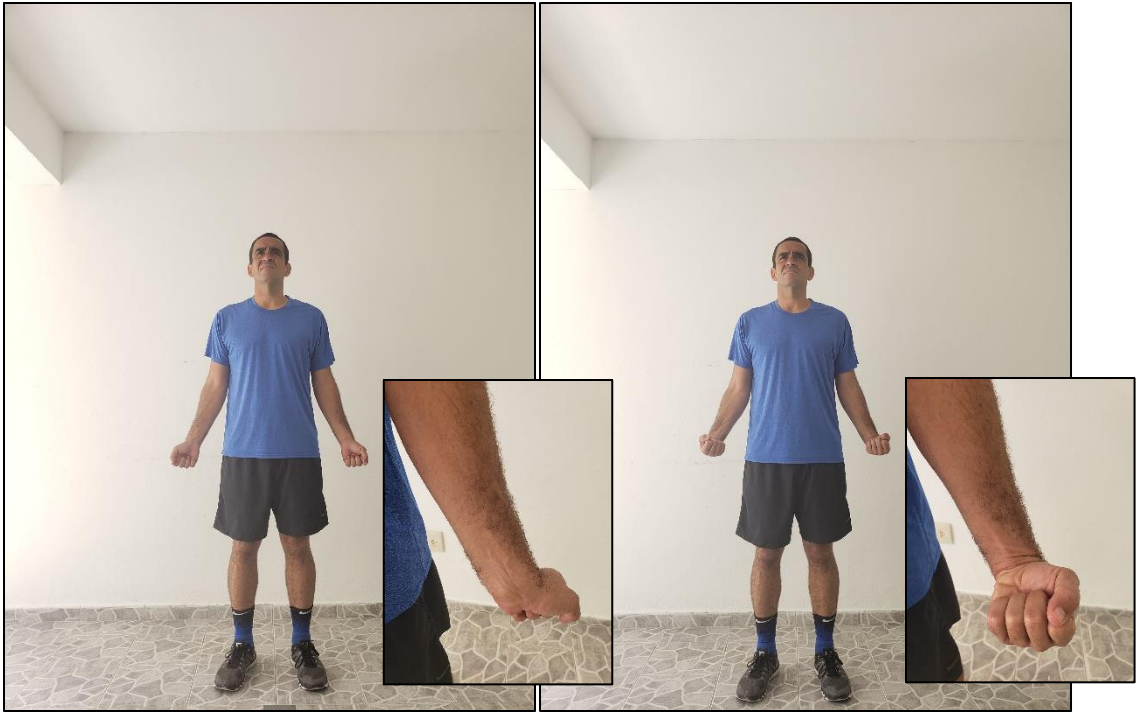

1.4. Dobre e estique os cotovelos

Quantidade: 10 repetições

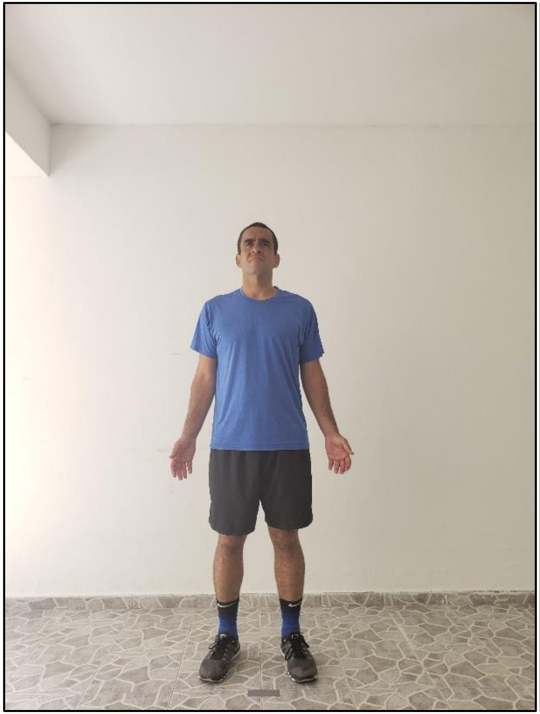

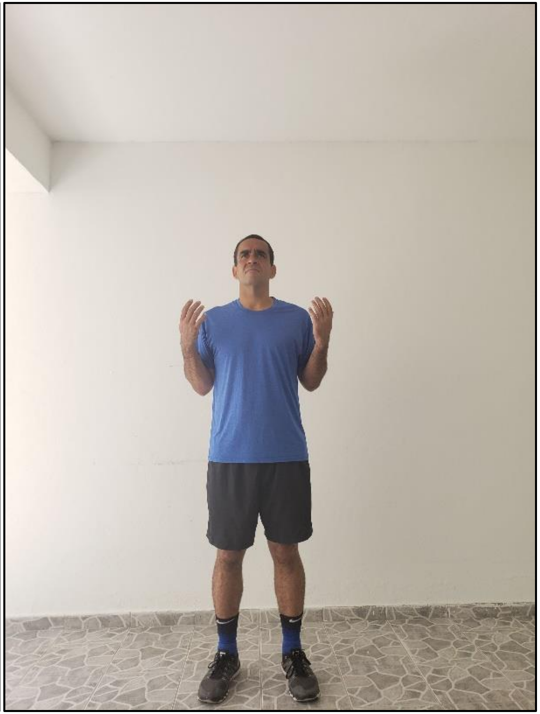

1.5. Eleve e abaixe as mãos acima da cabeça

Quantidade: 10 repetições

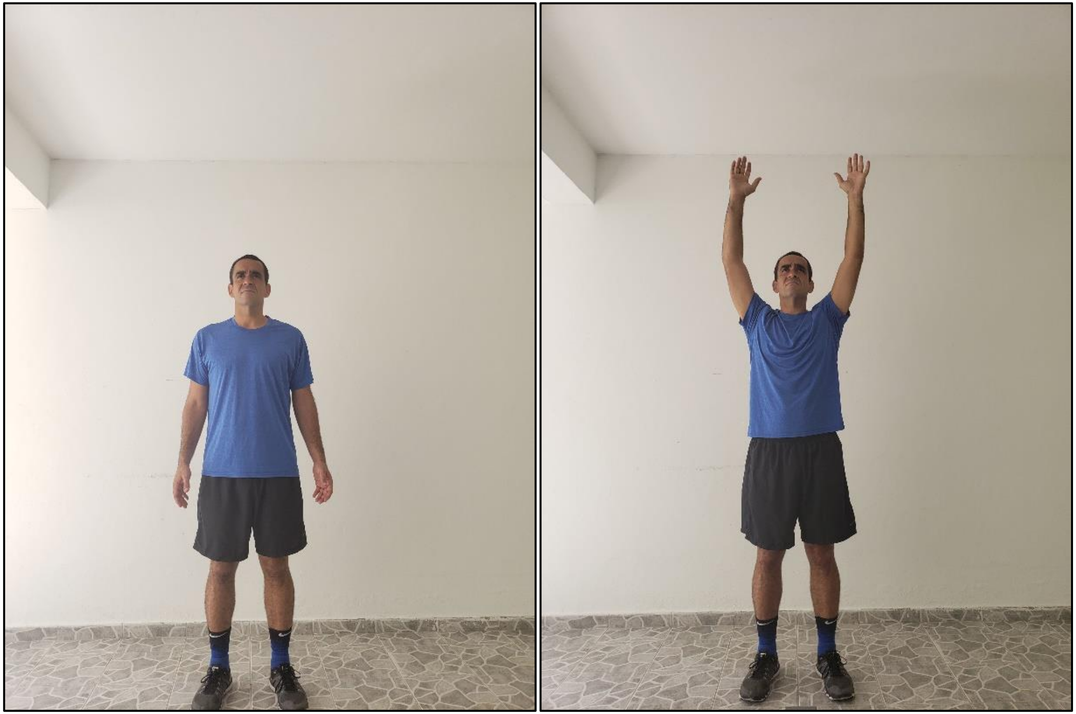

1.6. Eleve um joelho de cada vez até a altura da cintura e volte (apoio na parede)
Quantidade: 10 repetições

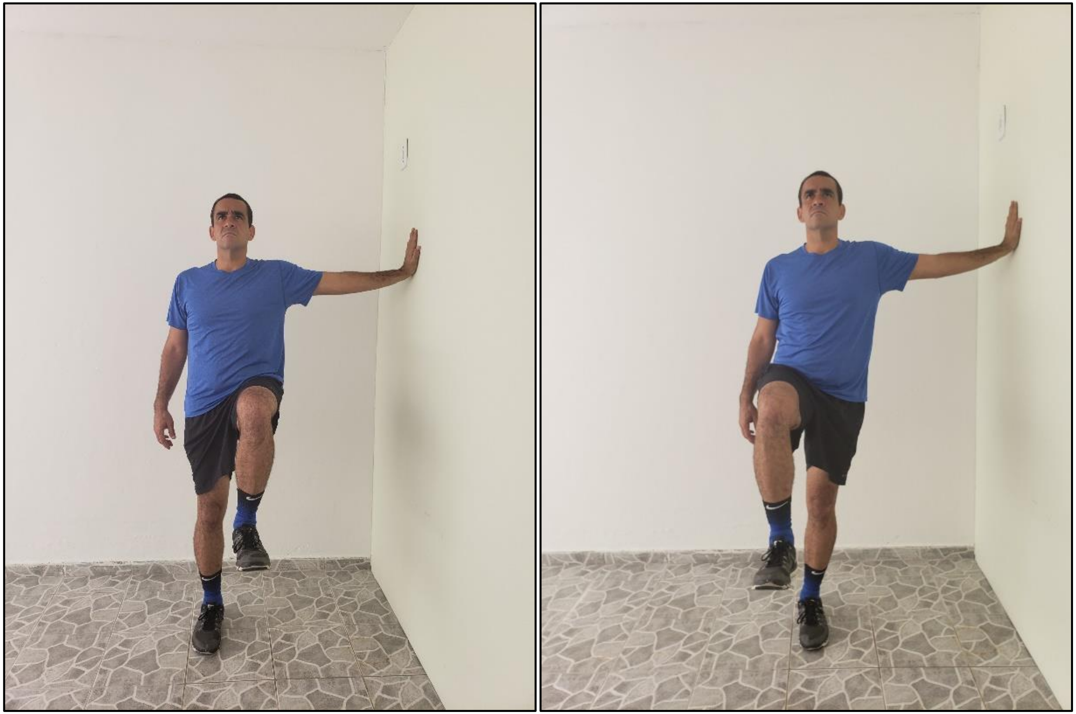

2. Parte Principal

2.1	Sentar e levantar	10 repetições
2.2	Caminhada para frente e para trás dobrando e esticando os braços	10 idas e voltas
2.3	Subir na ponta do pé	10 repetições
2.4	Passada lateral abrindo e fechando os braços	10 idas e voltas
2.5	Empurrar a parede	10 repetições
2.6	Caminhada no lugar	2 minutos
2.7	Puxar um braço de cada vez com apoio	10 repetições
2.8	Elevação alternada de braço e perna	10 repetições

Importante: Descanse 1 minuto entre os exercícios

2.1. Sentar e levantar

Quantidade: 10 repetições

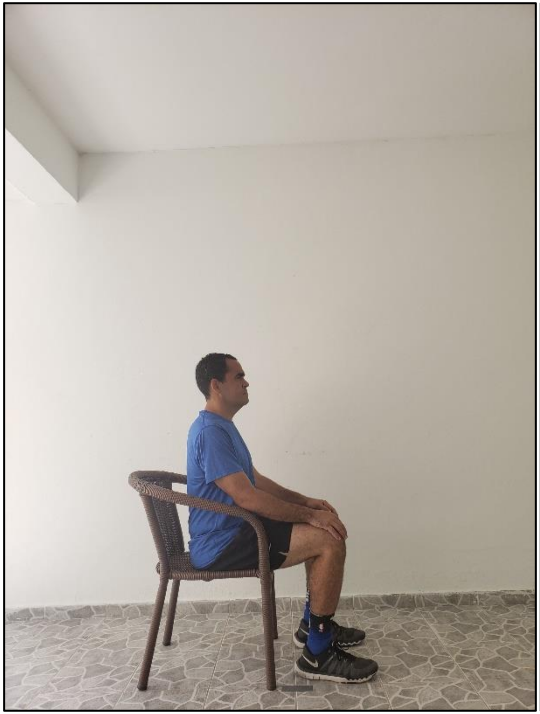

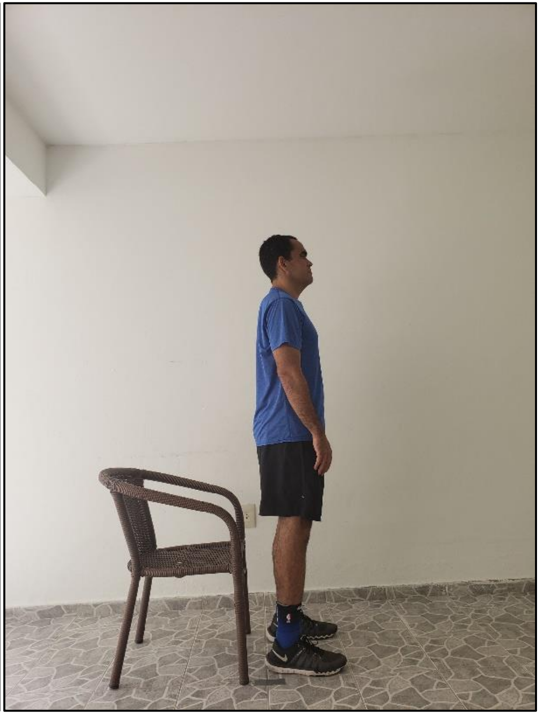

2.2. Caminhada para frente e para trás dobrando e esticando os braços

Quantidade: 10 idas e voltas

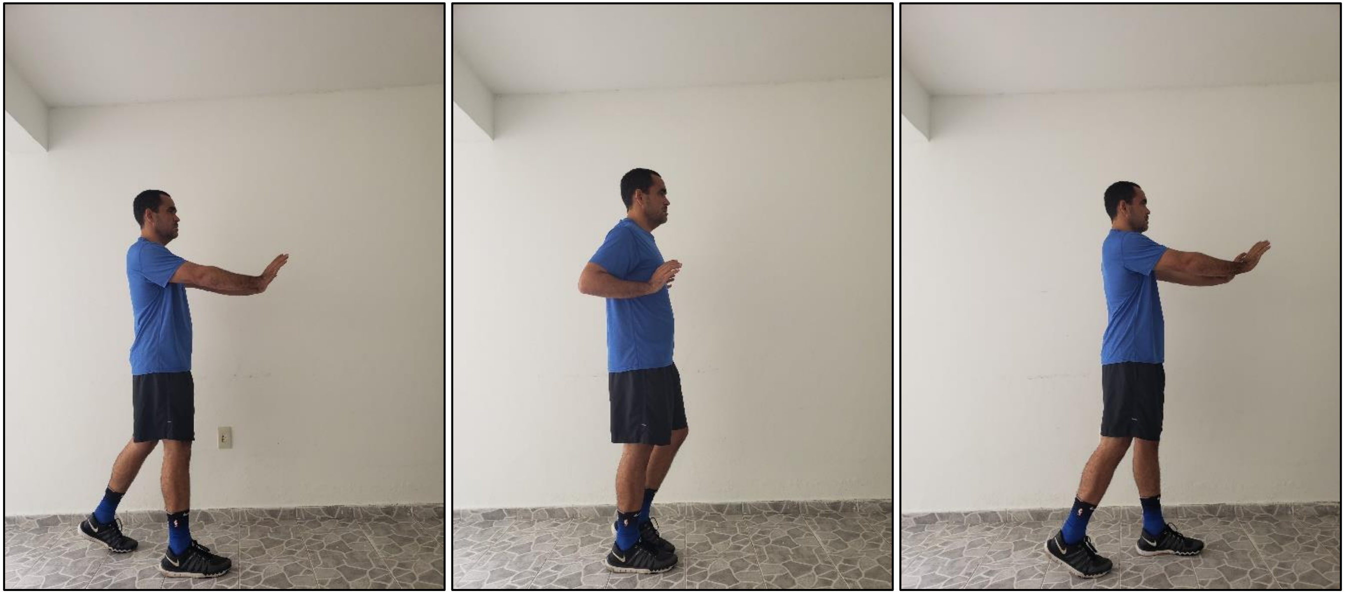

2.3. Subir na ponta do pé (apoio na parede)

Quantidade: 10 repetições

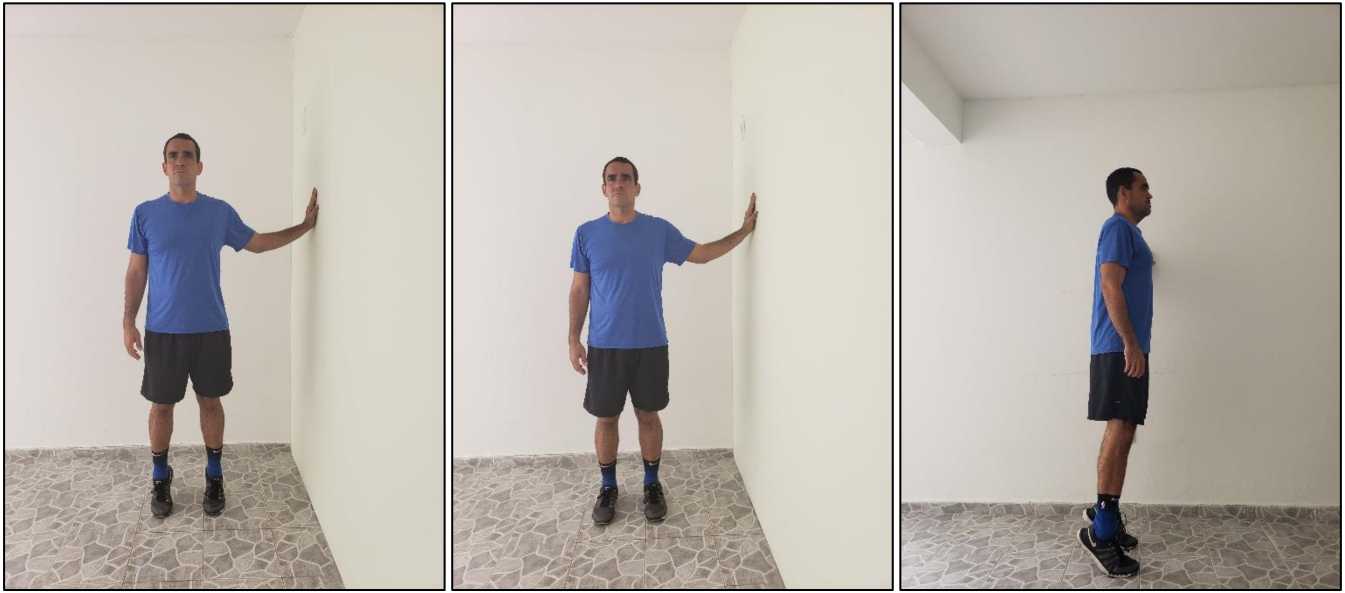

2.4. Passada lateral abrindo e fechando os braços

Quantidade: 10 idas e voltas

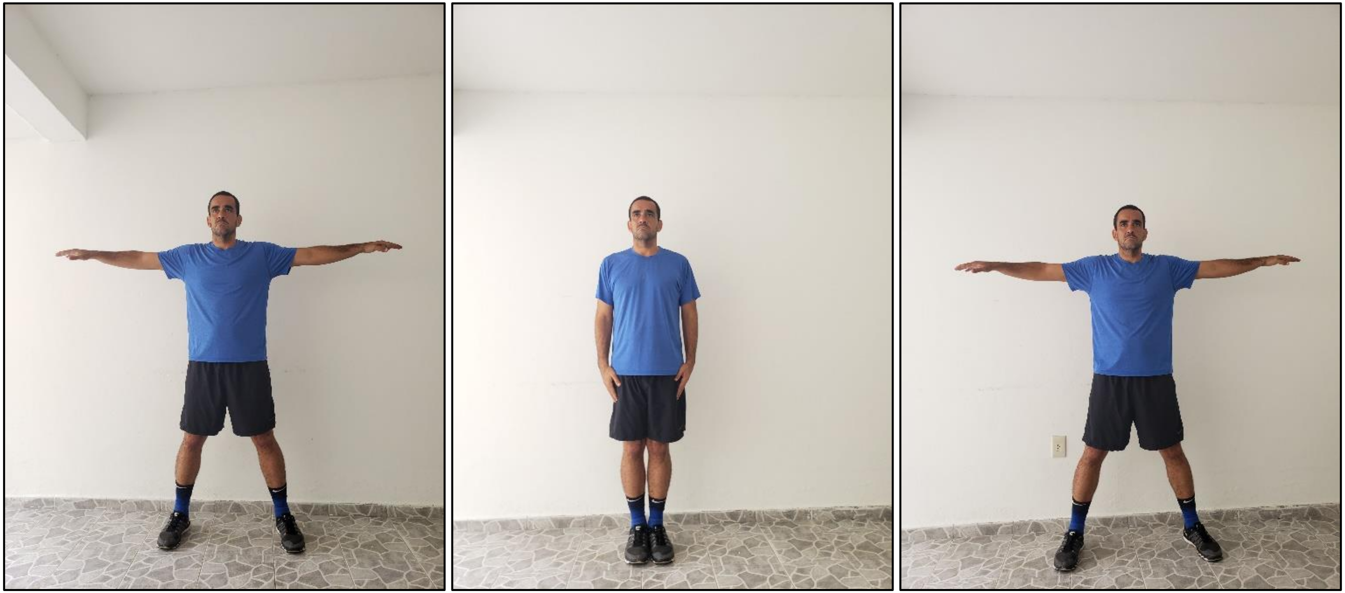

2.5. Empurrar a parede

Quantidade: 10 repetições

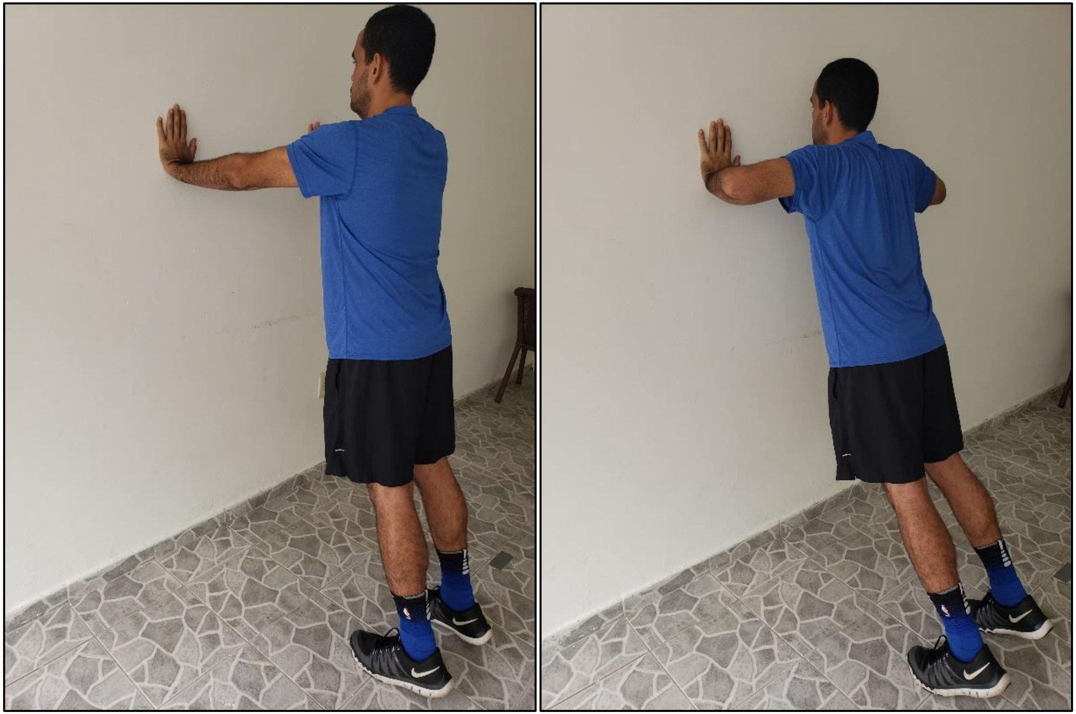

2.6. Caminhada no lugar

Quantidade: 2 minutos

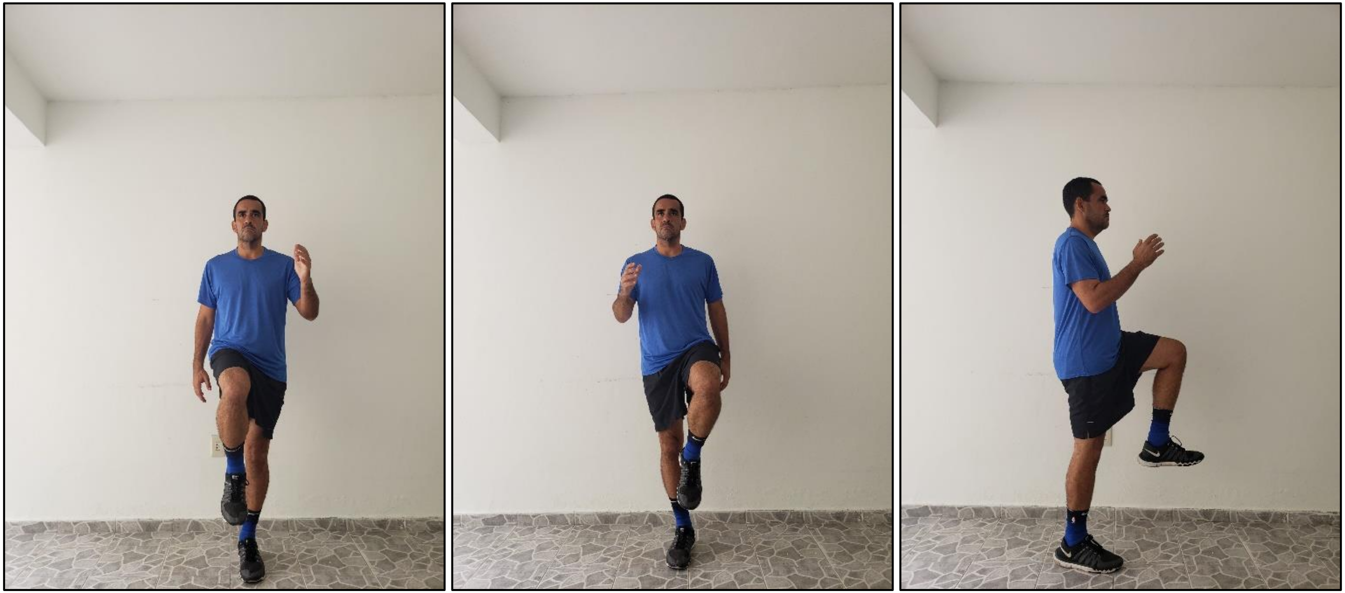

2.7. Puxar um braço de cada vez com apoio

Quantidade: 10 repetições

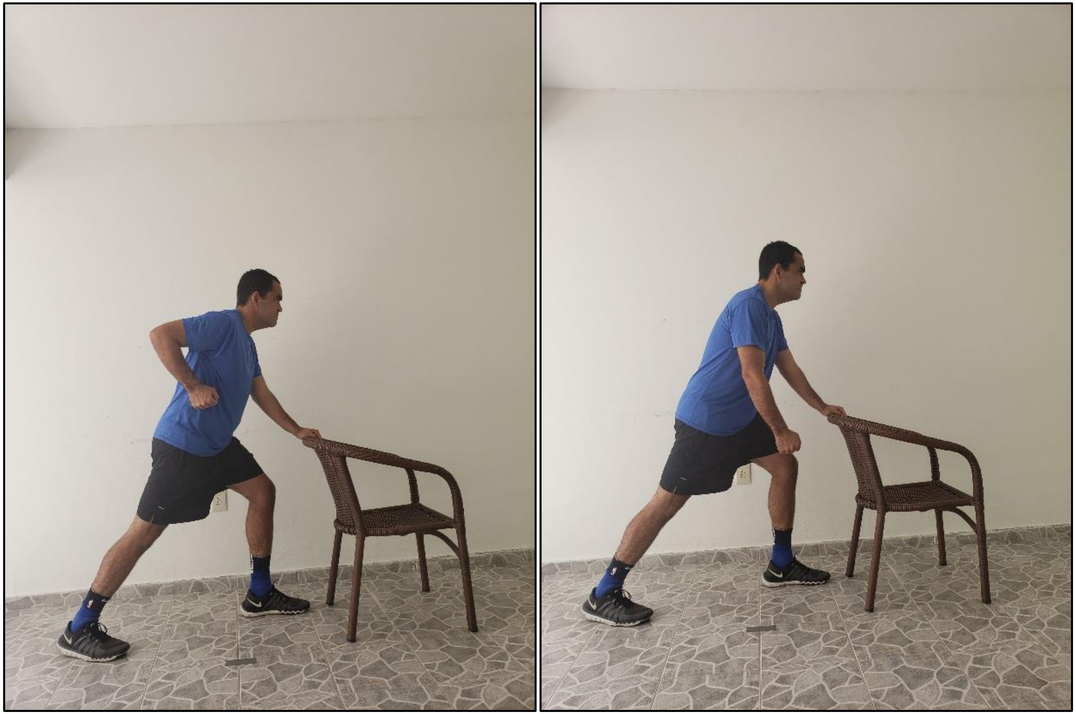

2.8. Elevação alternada de braço e perna

Quantidade: 10 repetições

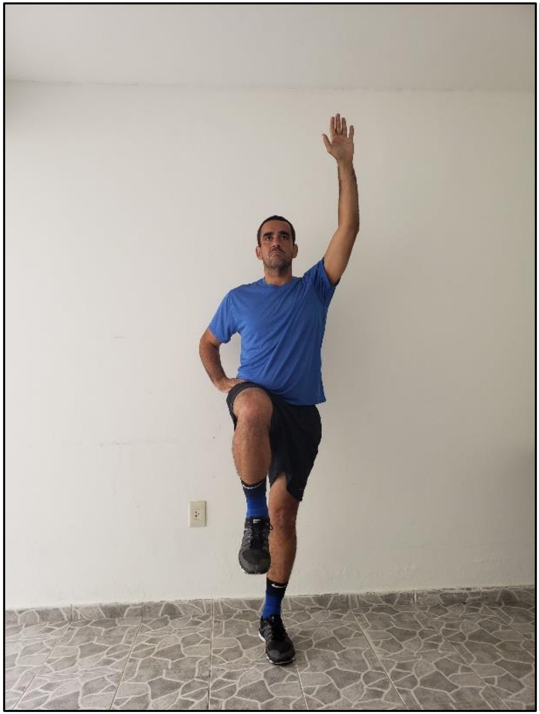

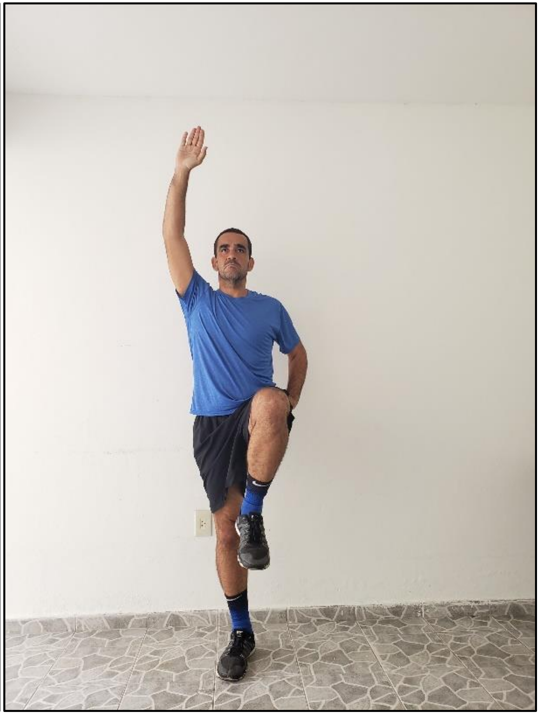

3. Volta à calma

3.1	Alongamento 1	15 segundos
3.2	Alongamento 2	15 segundos
3.3	Alongamento 3	15 segundos
3.4	Alongamento 4	15 segundos

3.1. Alongamento 1

Quantidade: 15 segundos para cada lado (2 vezes)

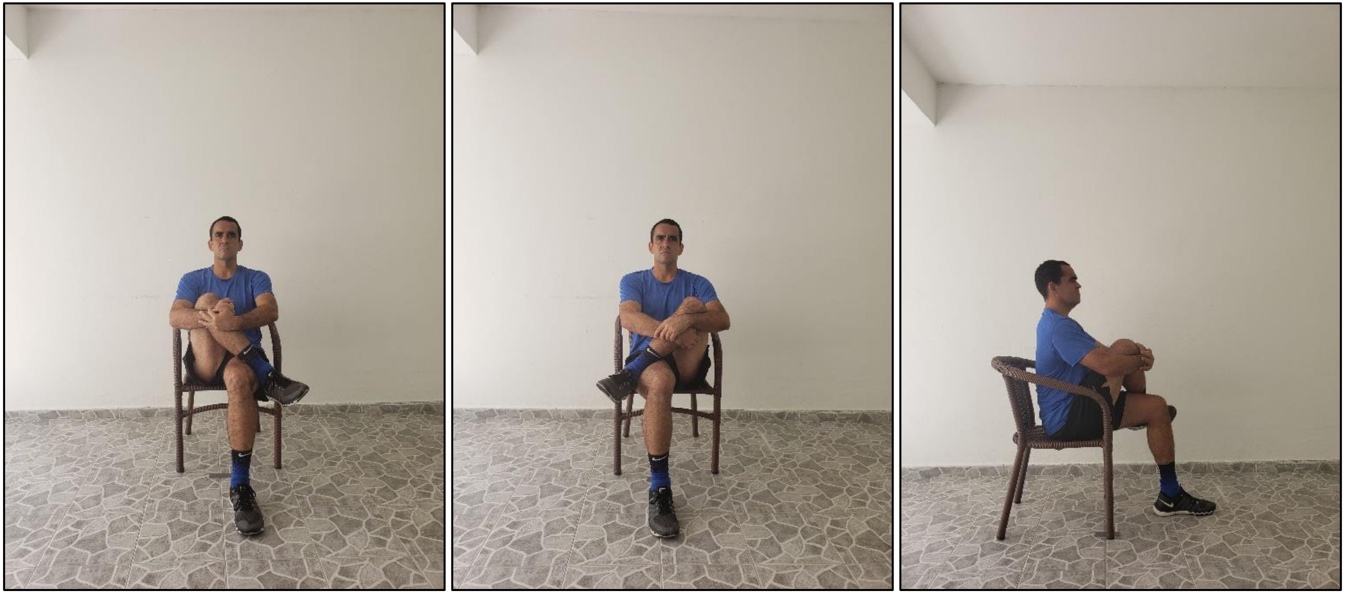

3.2. Alongamento 2

Quantidade: 15 segundos para cada lado (2 vezes)

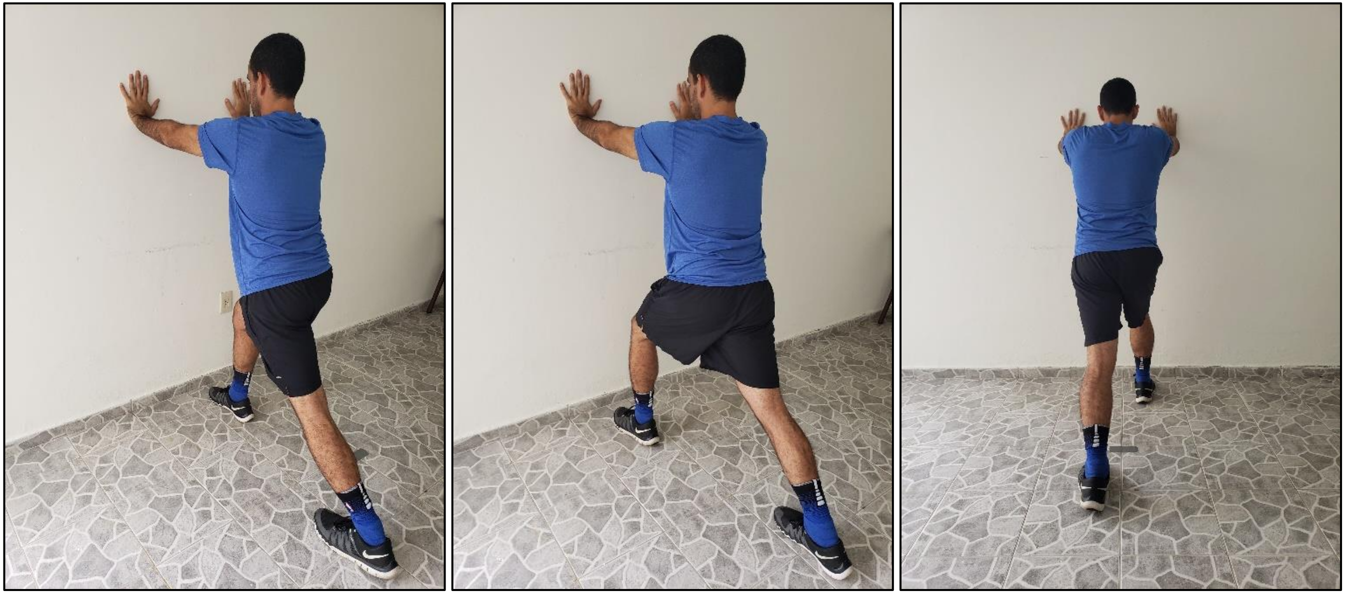

3.3. Alongamento 3

Quantidade: 15 segundos para cada lado (2 vezes)

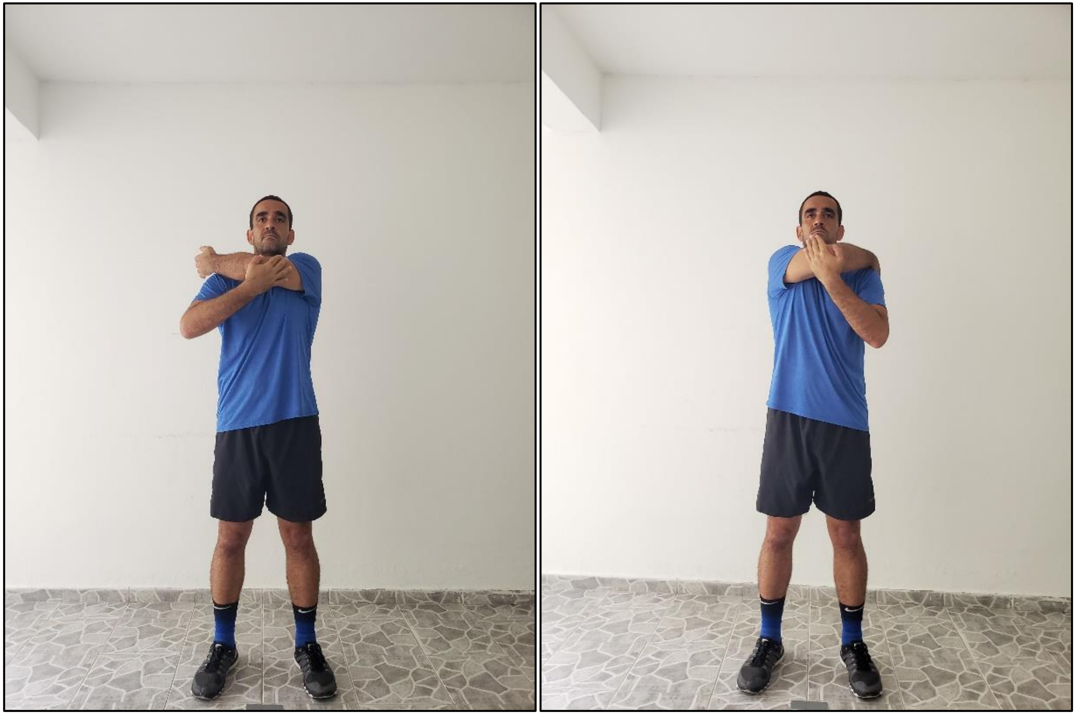

3.4. Alongamento 4

Quantidade: 15 segundos para cada lado (2 vezes)

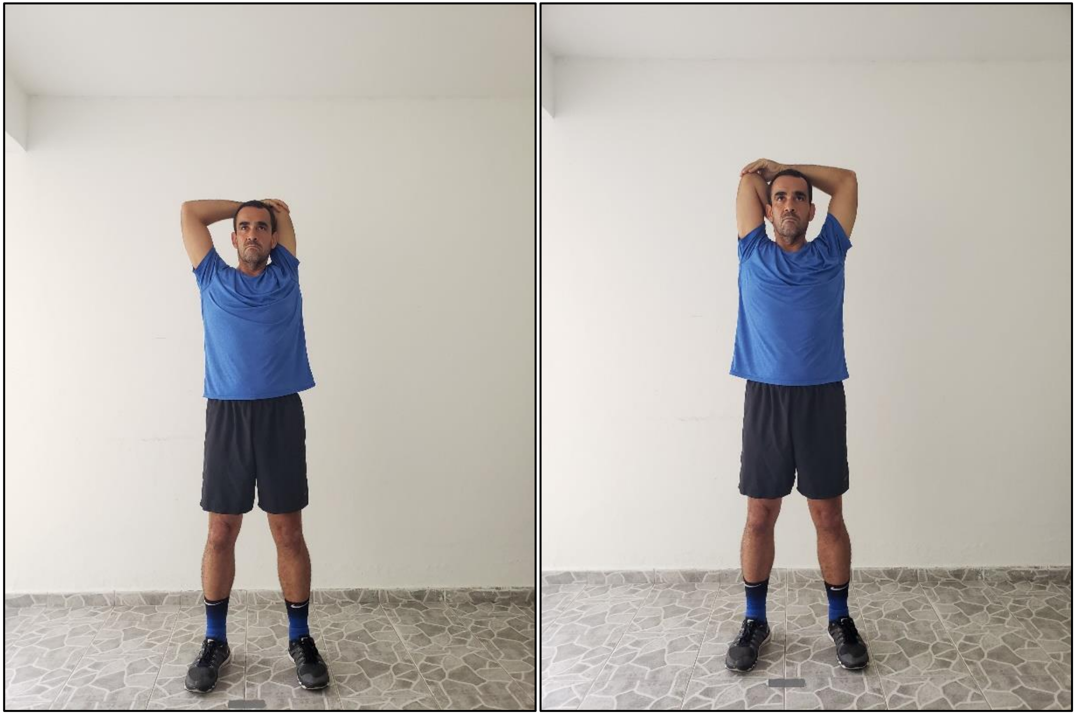

4. Controle de participação:

Marque abaixo os dias em que você conseguiu fazer os exercícios

Janeiro					1	2	3	4	5	6	7	8	9	10	11	12	13
14	15	16	17	18	19	20	21	22	23	24	25	26	27	28	29	30	31

Fevereiro					1	2	3	4	5	6	7	8	9	10	11	12	13
14	15	16	17	18	19	20	21	22	23	24	25	26	27	28			

Março					1	2	3	4	5	6	7	8	9	10	11	12	13
14	15	16	17	18	19	20	21	22	23	24	25	26	27	28	29	30	31

Abril					1	2	3	4	5	6	7	8	9	10	11	12	13
14	15	16	17	18	19	20	21	22	23	24	25	26	27	28	29	30	

Maio					1	2	3	4	5	6	7	8	9	10	11	12	13
14	15	16	17	18	19	20	21	22	23	24	25	26	27	28	29	30	31

Junho					1	2	3	4	5	6	7	8	9	10	11	12	13
14	15	16	17	18	19	20	21	22	23	24	25	26	27	28	29	30	

Julho					1	2	3	4	5	6	7	8	9	10	11	12	13
14	15	16	17	18	19	20	21	22	23	24	25	26	27	28	29	30	31

Agosto					1	2	3	4	5	6	7	8	9	10	11	12	13
14	15	16	17	18	19	20	21	22	23	24	25	26	27	28	29	30	31

Setembro					1	2	3	4	5	6	7	8	9	10	11	12	13
14	15	16	17	18	19	20	21	22	23	24	25	26	27	28	29	30	

Outubro					1	2	3	4	5	6	7	8	9	10	11	12	13
14	15	16	17	18	19	20	21	22	23	24	25	26	27	28	29	30	31

Novembro					1	2	3	4	5	6	7	8	9	10	11	12	13
14	15	16	17	18	19	20	21	22	23	24	25	26	27	28	29	30	

Dezembro					1	2	3	4	5	6	7	8	9	10	11	12	13
14	15	16	17	18	19	20	21	22	23	24	25	26	27	28	29	30	31
