# Supplementary material for: Home-based exercise program in the indeterminate form of Chagas disease (PEDI-CHAGAS study): A study protocol for a randomized clinical trial
Source: Front Med (Lausanne). 2023 Jan 6;9:1087188. doi: 10.3389/fmed.2022.1087188 (PMC9852894; doi:10.3389/fmed.2022.1087188)
Supplement: Supplementary file 4 [file Data_Sheet_4.PDF]

FIOCRUZ
FUNDAÇÃO OSWALDO CRUZ

INI
Instituto Nacional de Infectologia Evandro Chagas

PROGRAMA DE EXERCÍCIOS DOMICILIAR

Programa 2/2

2

Olá,

Assim como no programa 1, os exercícios devem ser realizados três vezes por semana, com duração de 60 minutos por dia.

Mais uma vez, muito obrigado pela sua participação,

Equipe Lapclin-Chagas

Orientações e recomendações

1. Esses exercícios ajudarão você a se sentir melhor no seu dia a dia.
2. Utilize roupas leves e calçado confortável, sem salto e que não escorregue.
3. Não realize os exercícios em horários muito quentes.
4. Durante a realização dos exercícios, você poderá ficar um pouco cansado(a). Mantenha um esforço no qual seja possível conversar com alguém ao seu lado, caso seja necessário.
5. Execute os exercícios com movimentos contínuos e equilibrados.
6. Respire normalmente durante a execução dos exercícios. Não prenda a respiração!
7. Descanse por um minuto entre os exercícios.
8. Ao término dos exercícios, realize os alongamentos, que devem ser repetidos 2 vezes por cerca de 15 segundos, até o limite onde você perceba um leve desconforto no local do alongamento.
9. Nenhum exercício pode causar dor. Caso isso ocorra, pare o exercício imediatamente.
10. A sessão de exercícios não deverá ser iniciada em caso de sintomas como cansaço, falta de ar, dor no peito ou mal-estar.
11. Em caso de dúvidas entre em contato com o Dr. Mauro Mediano (98521-0609), Prof. Leonardo Ribeiro (97254-0977) ou Centro Cardiológico do INI (3865-9648).

A sua sessão de exercícios tem três etapas a serem realizadas:

1. Aquecimento

2. Parte principal

3. Volta à calma

Progressão dos exercícios

13ª e 14ª semanas: Realizar cada exercício 2 vezes

15ª a 24ª semanas: Realizar cada exercício 3 vezes

1. Aquecimento (Executar os movimentos lentamente)

1.1	De pé, braços ao longo do corpo, incline o tronco com as mãos tocando as coxas	10 repetições
1.2	Abra e feche as mãos	10 repetições
1.3	Mãos fechadas, dobre e estique os punhos	10 repetições
1.4	Dobre e estique os cotovelos	10 repetições
1.5	Eleve e abaixe as mãos acima da cabeça	10 repetições
1.6	Eleve um joelho de cada vez até a altura da cintura e volte (apoio na parede)	10 repetições

1.1. De pé, braços ao longo do corpo, incline o tronco com as mãos tocando as coxas

Quantidade: 10 repetições

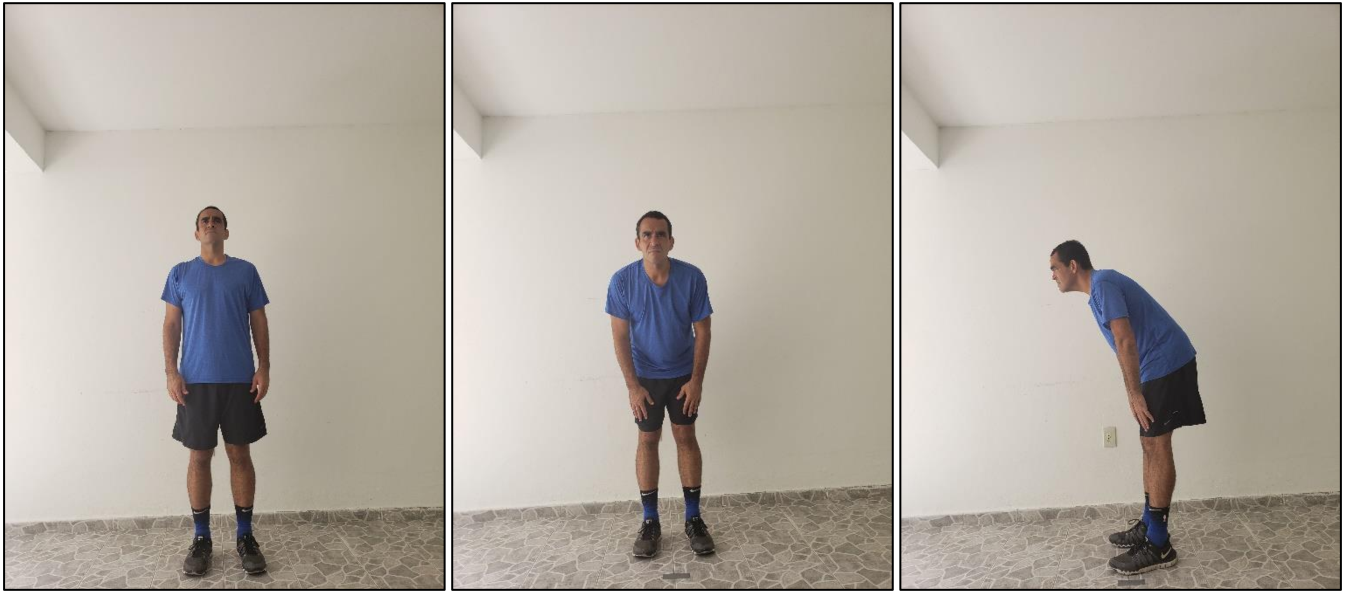

1.2. Abra e feche as mãos

Quantidade: 10 repetições

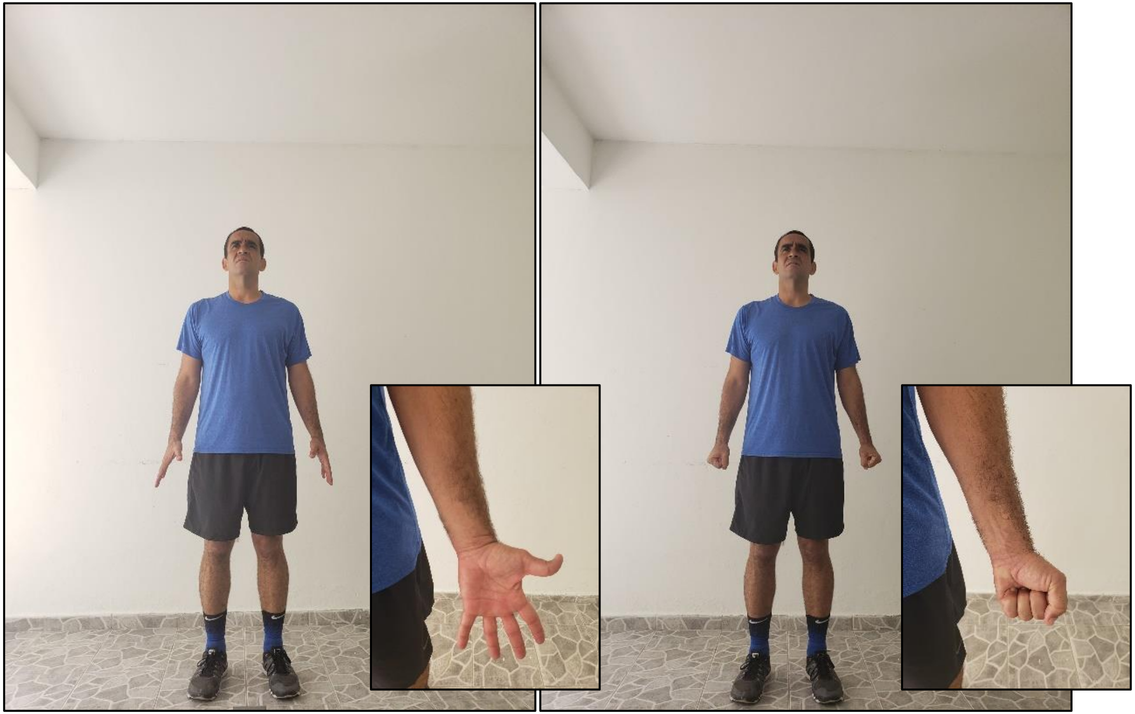

1.3. Mãos fechadas, dobre e estique os punhos

Quantidade: 10 repetições

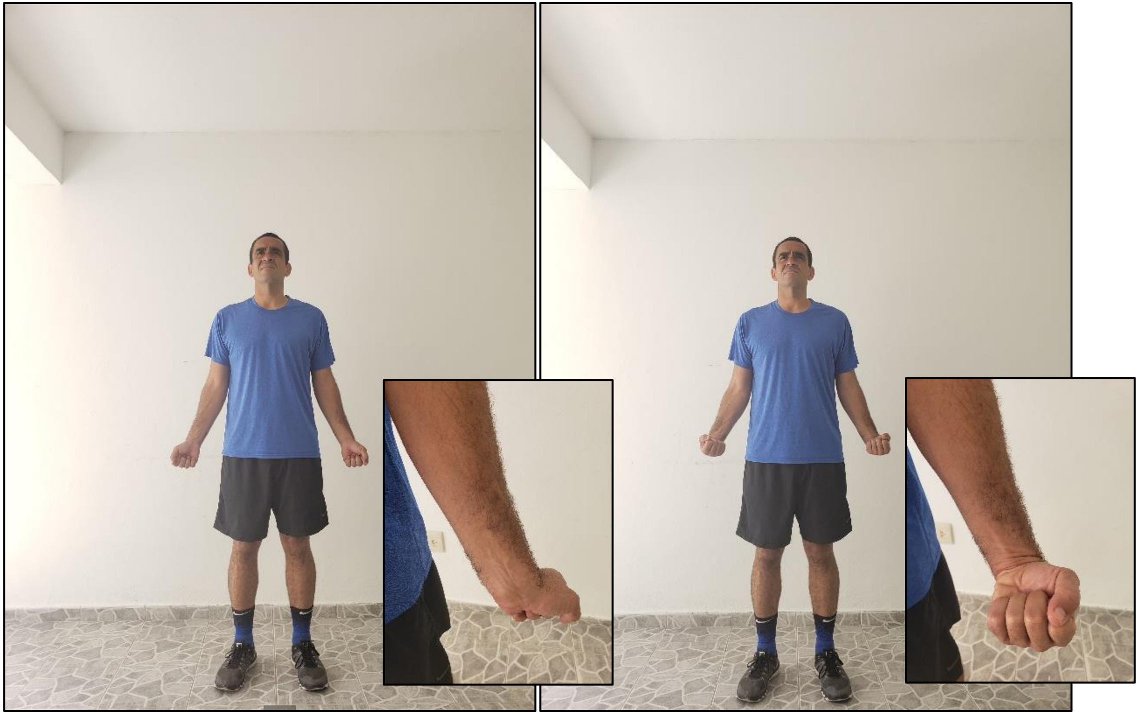

1.4. Dobre e estique os cotovelos

Quantidade: 10 repetições

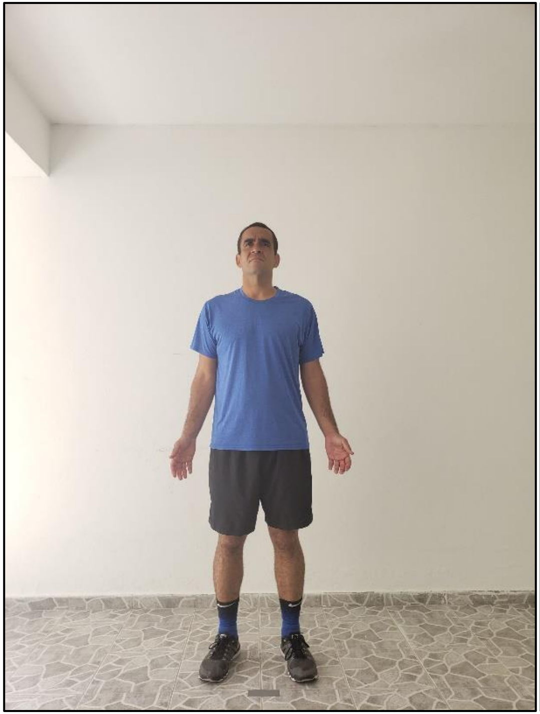

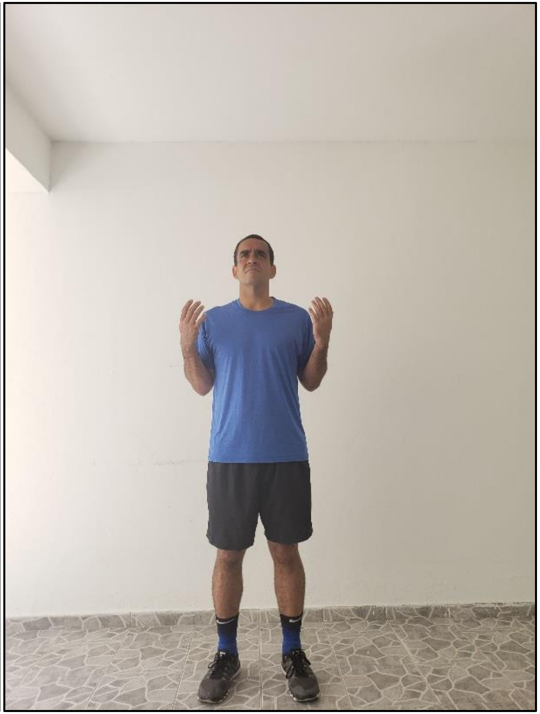

1.5. Eleve e abaixe as mãos acima da cabeça

Quantidade: 10 repetições

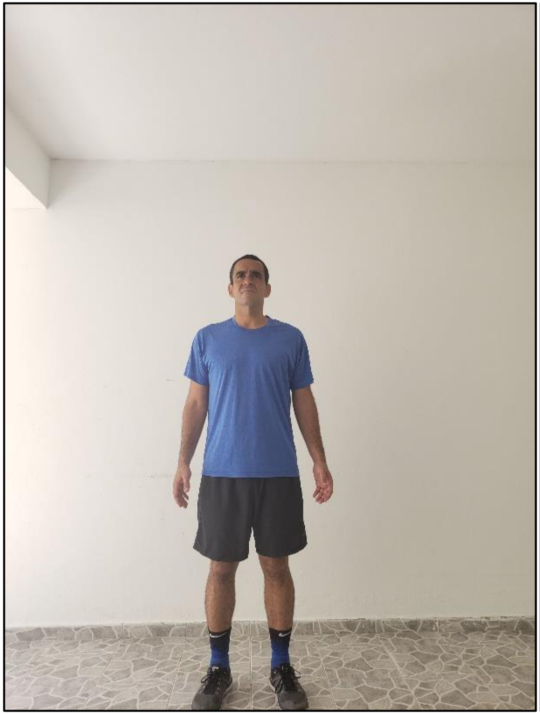
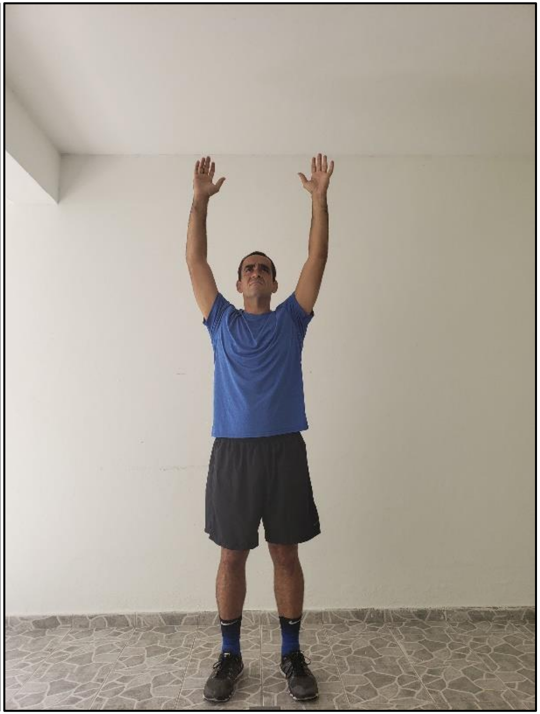

1.6. Eleve um joelho de cada vez até a altura da cintura e volte (apoio na parede)
Quantidade: 10 repetições

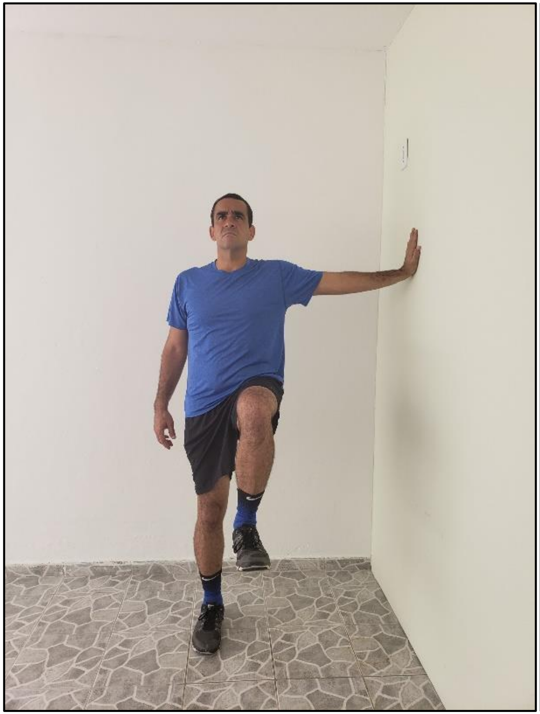

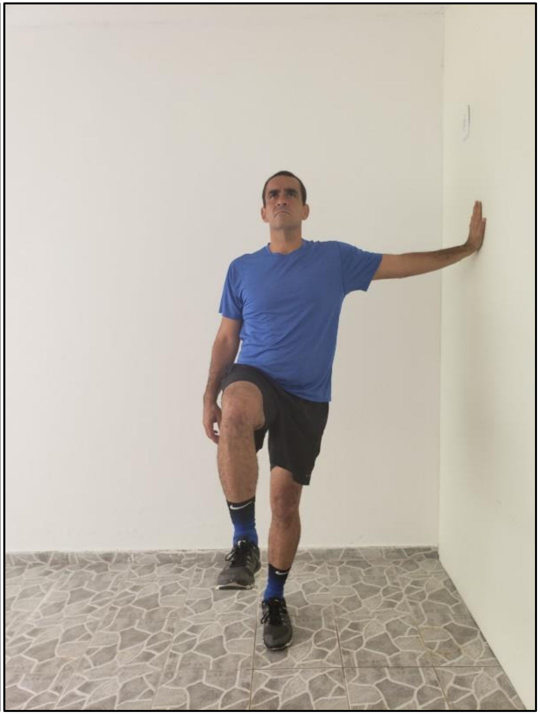

2. Parte Principal

2.1	Caminhada no lugar	2 minutos
2.2	Dobrar os cotovelos e elevar os braços acima da cabeça	10 repetições
2.3	Jogar a bola para o alto e pegar	10 repetições
2.4	Sentar e levantar	10 repetições
2.5	Rotação de tronco com elevação dos joelhos	10 repetições
2.6	Quicar a bola andando para frente e para trás	10 repetições
2.7	Dobrar os joelhos (uma perna de cada vez)	10 repetições
2.8	Polichinelo adaptado (mão na cabeça ficando na ponta do pé)	10 repetições

Importante: Descanse 1 minuto entre os exercícios

2.1. Caminhada no lugar

Quantidade: 2 minutos

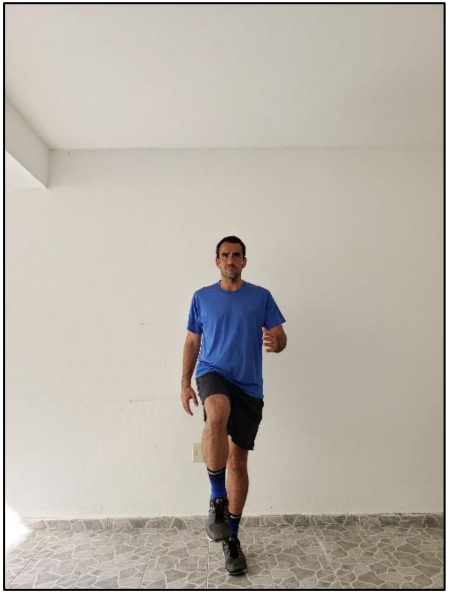
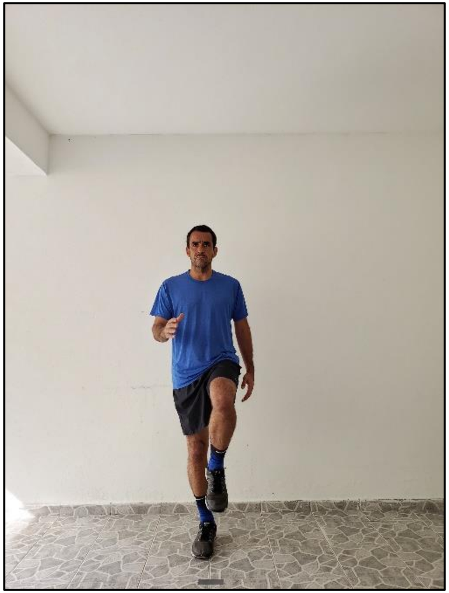
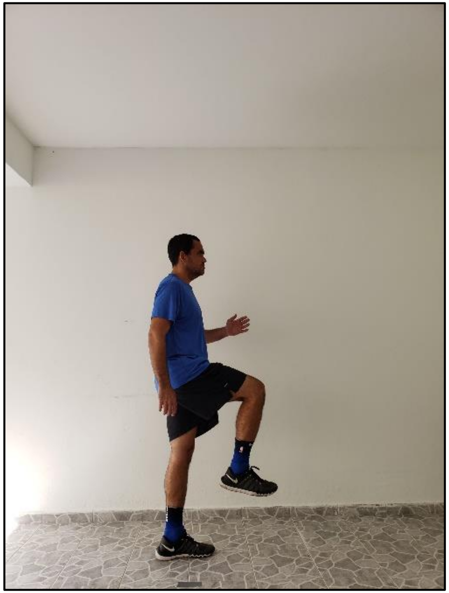

2.2. Dobrar os cotovelos e elevar os braços acima da cabeça

Quantidade: 10 repetições

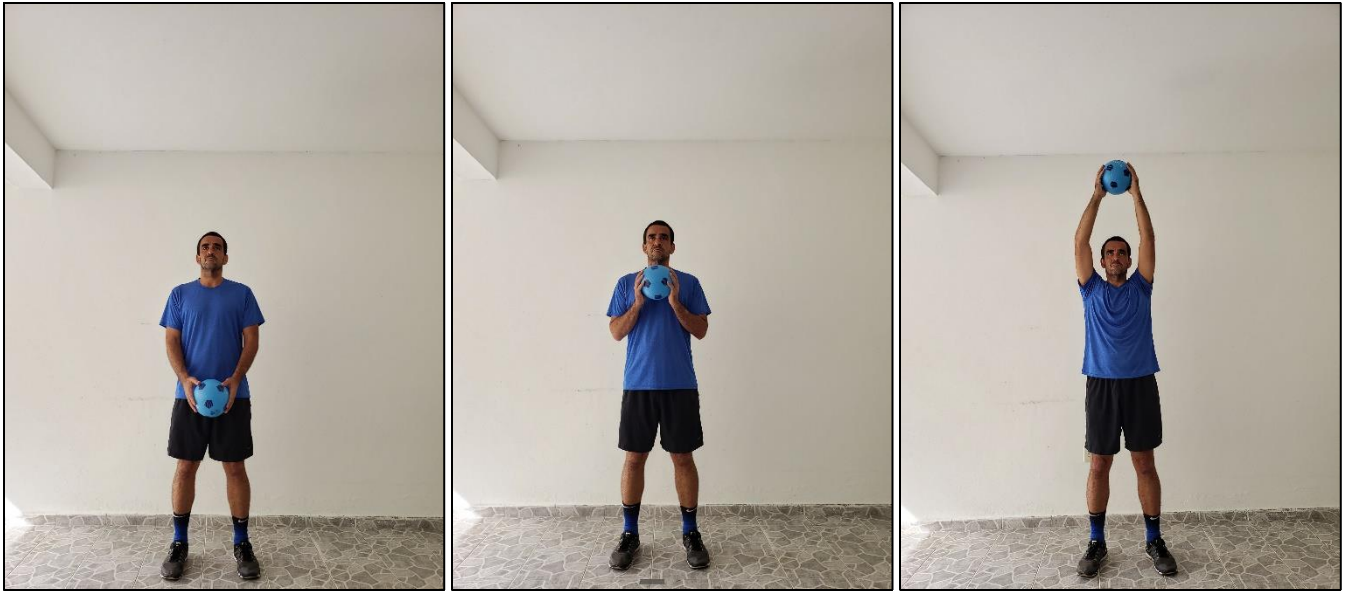

2.3. Jogar a bola para o alto e pegar

Quantidade: 10 repetições

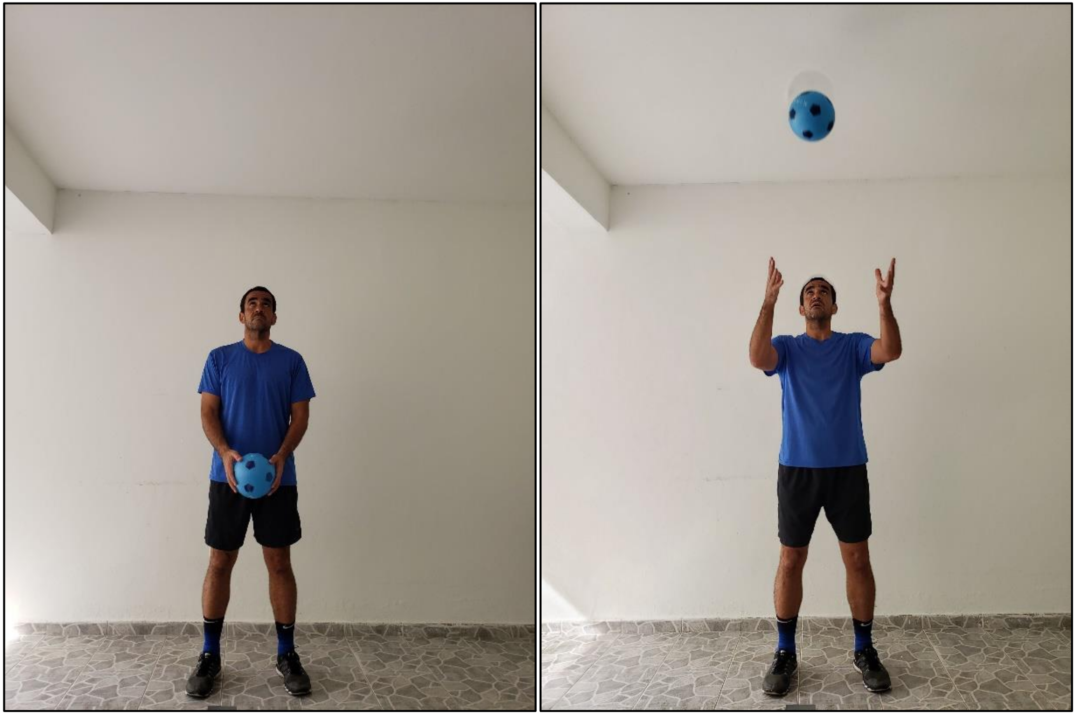

2.4. Sentar e levantar

Quantidade: 10 repetições

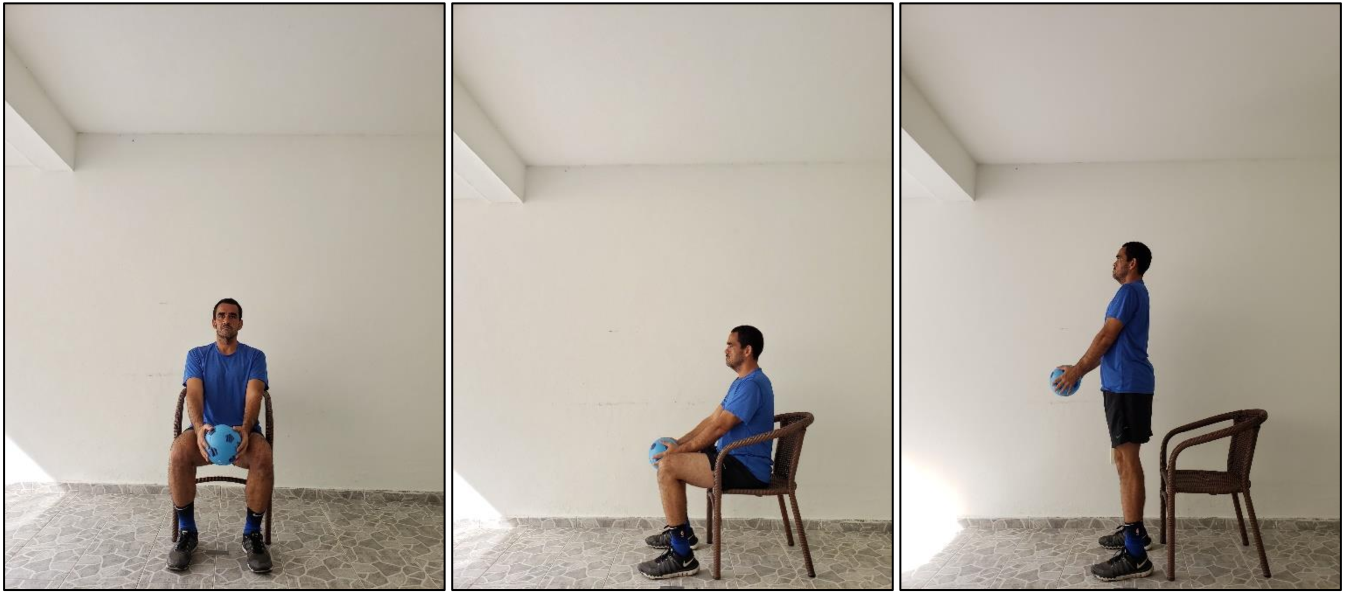

2.5. Rotação de tronco com elevação dos joelhos

Quantidade: 10 repetições

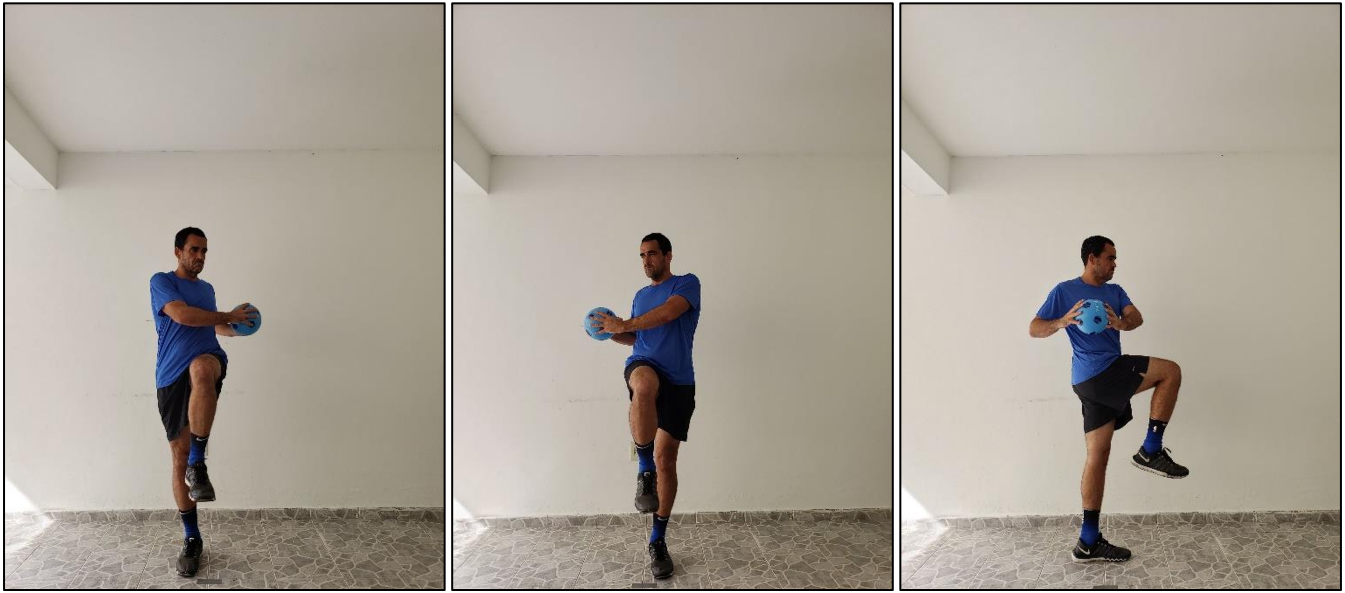

2.6. Quicar a bola andando para frente e para trás

Quantidade: 10 repetições

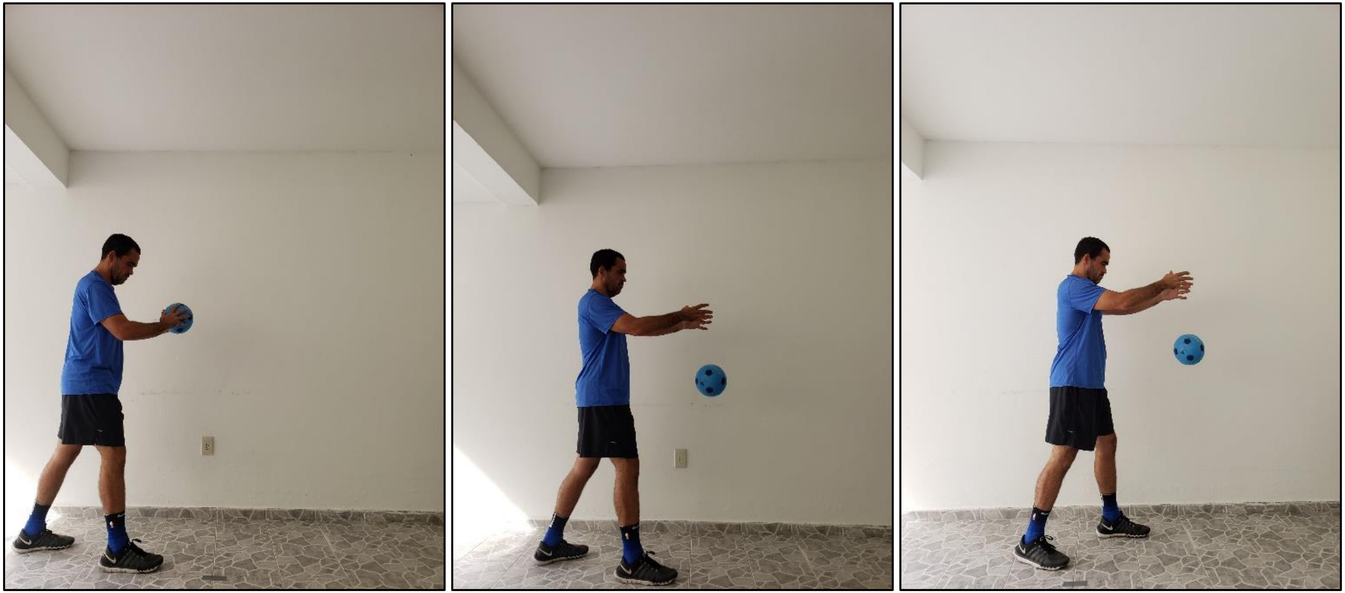

2.7. Dobrar os joelhos (uma perna de cada vez)

Quantidade: 10 repetições

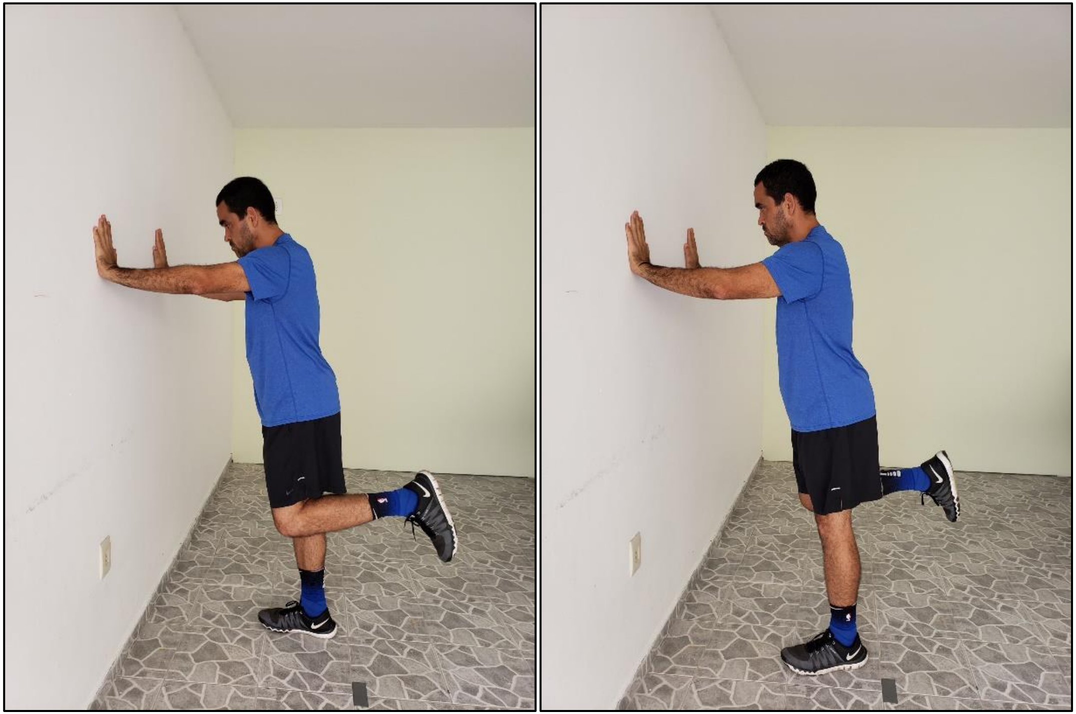

2.8. Polichinelo adaptado (mão na cabeça ficando na ponta do pé)

Quantidade: 10 repetições

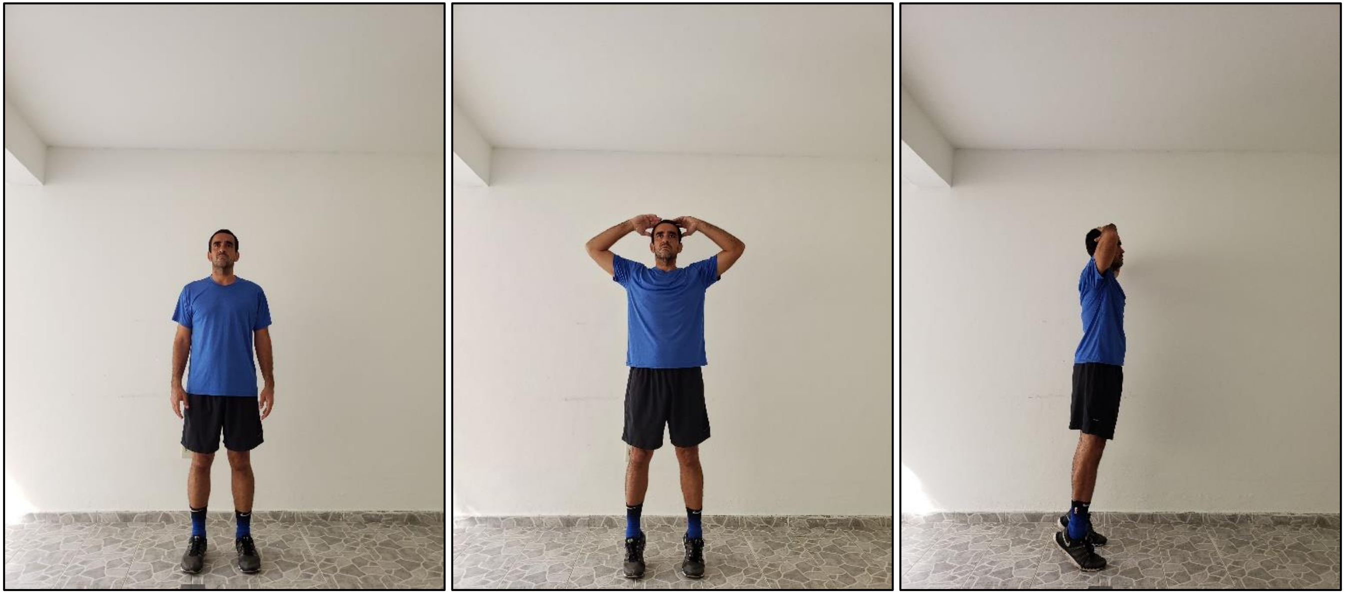

3. Volta à calma

3.1	Alongamento 1	15 segundos
3.2	Alongamento 2	15 segundos
3.3	Alongamento 3	15 segundos
3.4	Alongamento 4	15 segundos

3.1. Alongamento 1

Quantidade: 15 segundos para cada lado (2 vezes)

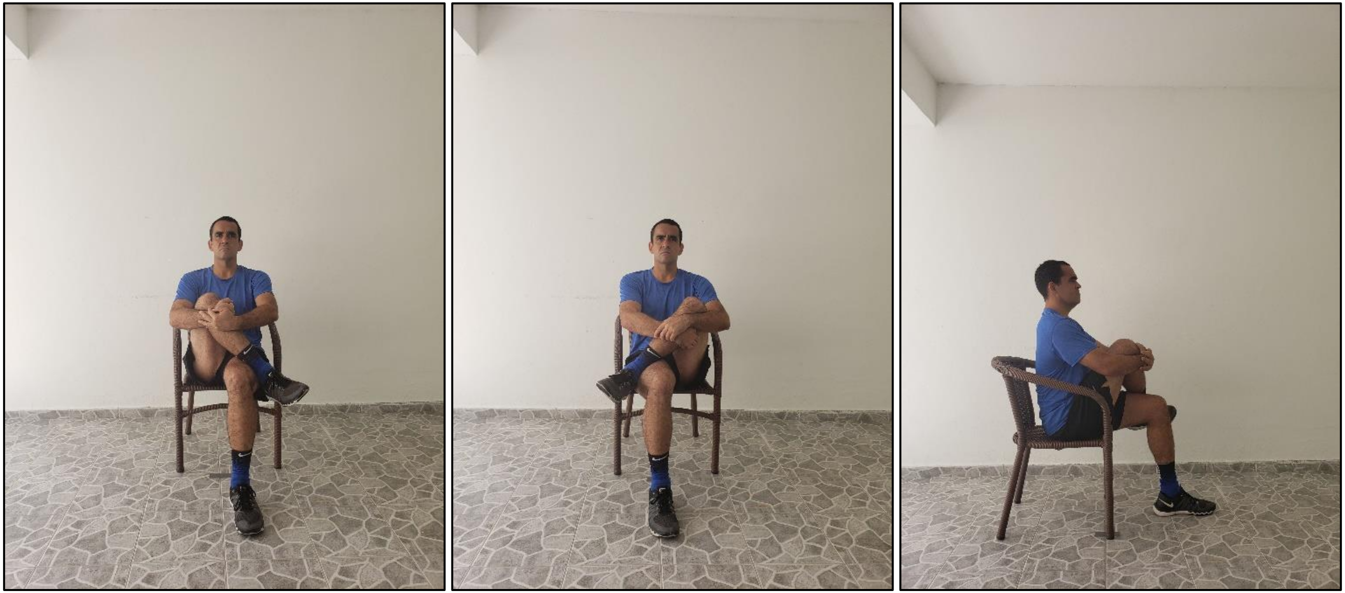

3.2. Alongamento 2

Quantidade: 15 segundos para cada lado (2 vezes)

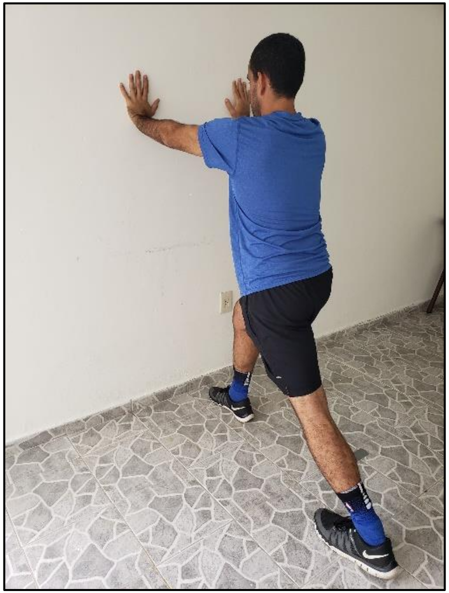
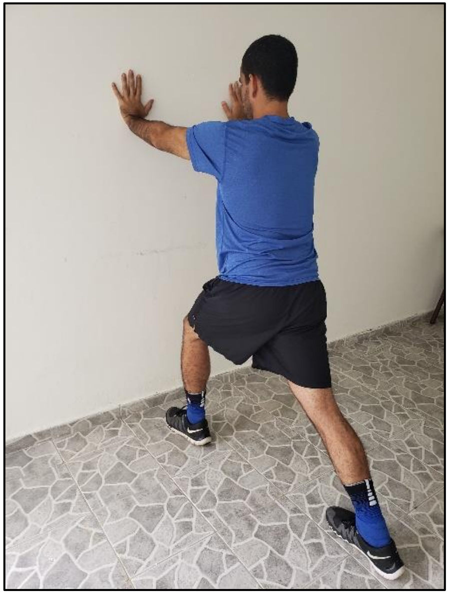
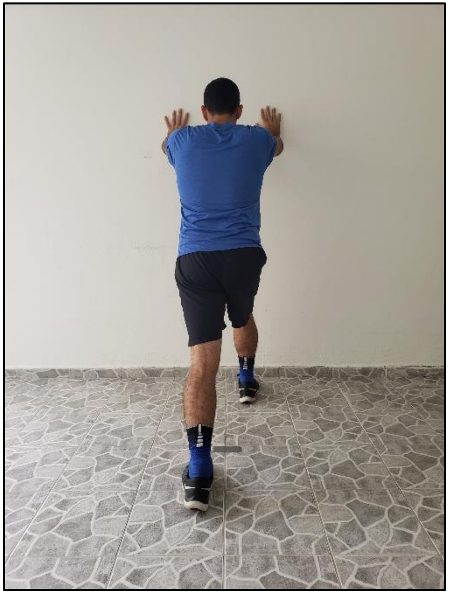

3.3. Alongamento 3

Quantidade: 15 segundos para cada lado (2 vezes)

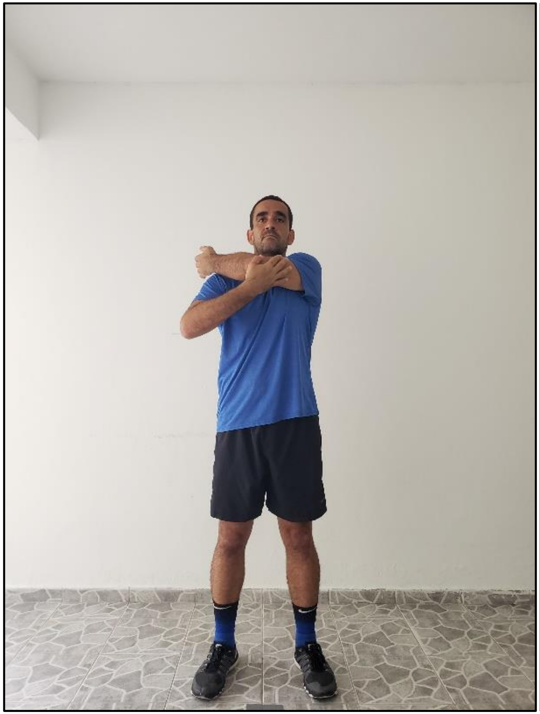

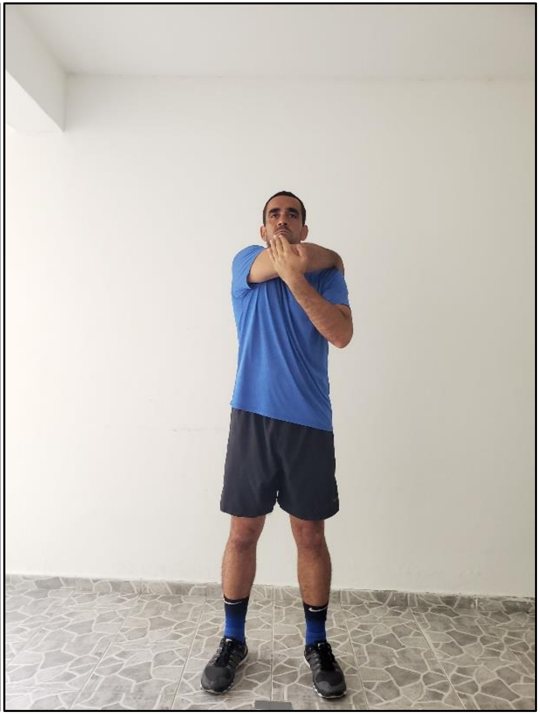

3.4. Alongamento 4

Quantidade: 15 segundos para cada lado (2 vezes)

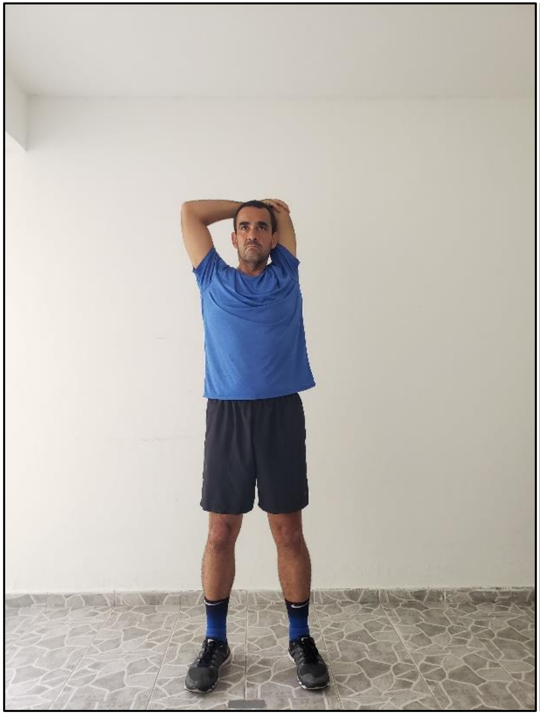

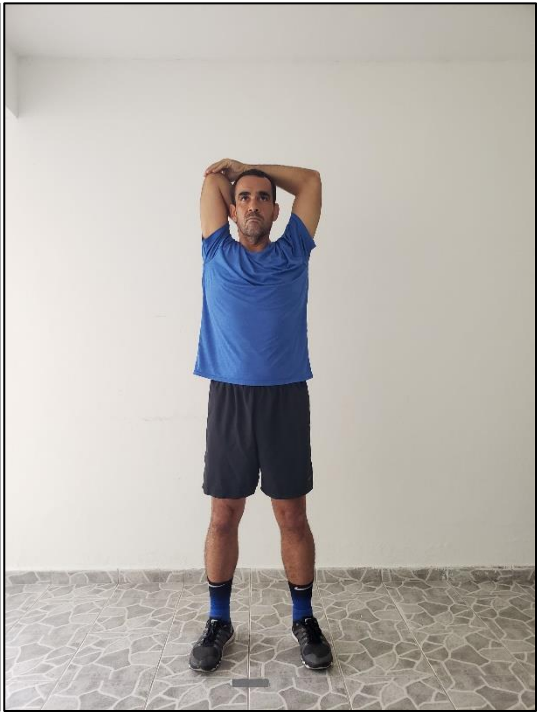

4. Controle de participação:

Marque abaixo os dias em que você conseguiu fazer os exercícios

Janeiro					1	2	3	4	5	6	7	8	9	10	11	12	13
14	15	16	17	18	19	20	21	22	23	24	25	26	27	28	29	30	31

Fevereiro					1	2	3	4	5	6	7	8	9	10	11	12	13
14	15	16	17	18	19	20	21	22	23	24	25	26	27	28			

Março					1	2	3	4	5	6	7	8	9	10	11	12	13
14	15	16	17	18	19	20	21	22	23	24	25	26	27	28	29	30	31

Abril					1	2	3	4	5	6	7	8	9	10	11	12	13
14	15	16	17	18	19	20	21	22	23	24	25	26	27	28	29	30	

Maio					1	2	3	4	5	6	7	8	9	10	11	12	13
14	15	16	17	18	19	20	21	22	23	24	25	26	27	28	29	30	31

Junho					1	2	3	4	5	6	7	8	9	10	11	12	13
14	15	16	17	18	19	20	21	22	23	24	25	26	27	28	29	30	

Julho					1	2	3	4	5	6	7	8	9	10	11	12	13
14	15	16	17	18	19	20	21	22	23	24	25	26	27	28	29	30	31

Agosto					1	2	3	4	5	6	7	8	9	10	11	12	13
14	15	16	17	18	19	20	21	22	23	24	25	26	27	28	29	30	31

Setembro					1	2	3	4	5	6	7	8	9	10	11	12	13
14	15	16	17	18	19	20	21	22	23	24	25	26	27	28	29	30	

Outubro					1	2	3	4	5	6	7	8	9	10	11	12	13
14	15	16	17	18	19	20	21	22	23	24	25	26	27	28	29	30	31

Novembro					1	2	3	4	5	6	7	8	9	10	11	12	13
14	15	16	17	18	19	20	21	22	23	24	25	26	27	28	29	30	

Dezembro					1	2	3	4	5	6	7	8	9	10	11	12	13
14	15	16	17	18	19	20	21	22	23	24	25	26	27	28	29	30	31
